# Synthesis and In Vitro Anticancer Evaluation of Novel Chrysin and 7-Aminochrysin Derivatives

**DOI:** 10.3390/molecules25040888

**Published:** 2020-02-17

**Authors:** Szabolcs Mayer, Péter Keglevich, Péter Ábrányi-Balogh, Áron Szigetvári, Miklós Dékány, Csaba Szántay, László Hazai

**Affiliations:** 1 Department of Organic Chemistry and Technology, Faculty of Chemical Technology and Biotechnology, Budapest University of Technology and Economics, Gellért tér 4, H-1111 Budapest, Hungary; mayer.szabolcs@mail.bme.hu (S.M.); hazai@mail.bme.hu (L.H.); 2 Medicinal Chemistry Research Group, Institute of Organic Chemistry, Research Centre for Natural Sciences, Magyar tudósok krt. 2, H-1117 Budapest, Hungary; 3 Spectroscopic Research Department, Gedeon Richter Plc., P.O. Box 27, H-1475 Budapest, Hungary; szigetvaria@richter.hu (Á.S.); m.dekany@richter.hu (M.D.);

**Keywords:** flavonoid, chrysin, aminoflavonoid, anticancer activity, Smiles rearrangement

## Abstract

Chrysin is a naturally occurring flavonoid with mild anticancer activity. In this paper we report the synthesis of new chrysin derivatives alkylated with *N*-phenylchloroacetamides in position 7. A novel method was developed for the preparation of 7-aminochrysin derivatives via the Smiles rearrangement, resulting in diphenylamine-type compounds. In silico studies of the Smiles rearrangement were performed. We also present the in vitro antiproliferative activity of the synthesized compounds against 60 human tumor cell lines (NCI60). The most potent derivative exhibited nanomolar antitumor activity on the MCF7 cell line of breast cancer (GI_50_ = 30 nM) and on the HCT-15 cell line of colon cancer (GI_50_ = 60 nM).

## 1. Introduction

**Figure 1:**
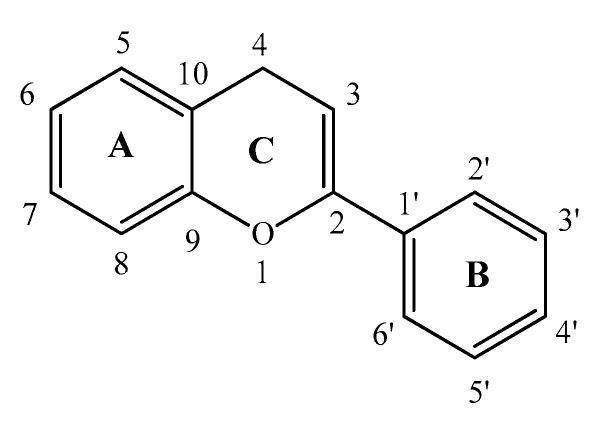
The 15-carbon skeleton of flavonoids.

Flavonoids are secondary plant metabolites containing numerous low-molecular-weight members [1,2]. Their general structure consists of a 15-carbon skeleton with a heterocyclic (pyran) ring (C) between two phenyl rings (A and B) (Figure 1). Flavonoids have a broad-spectrum biological activity including anti-inflammatory [3,4], antibacterial [5], antiviral [4,6,7], antioxidant [8,9], and antitumor [1,4] effects. Chrysin, also known as 5,7-dihydroxyflavone (**1**), is found in *Passiflora caerulea* [10,11], honey, and propolis. Like several flavonoids, it has a number of biological effects, of which it is important to mention its anticancer activity both as a chemopreventive and as a chemotherapeutic agent [12]. Chrysin (**1**), in addition to being used alone, can be given in combination, especially by reducing the resistance of the corresponding drug to increase the efficacy of the chemotherapeutic agent used in combination [13]. The goal of the present study is to modify position 7 of chrysin (**1**) with various aromatic acetamides, and to synthesize 7-aminochrysin derivatives via the Smiles rearrangement, resulting in the formation of diphenylamine-type compounds. We also conduct in silico studies of the reaction mechanisms and investigate the antiproliferative activity of the synthesized compounds.

Aminoflavone derivatives have been described as tyrosine kinase inhibitors [14]. These compounds can be obtained by the reduction of the corresponding nitro derivative [15], total synthesis of the aminoflavone derivative [16], or reduction following the palladium-catalyzed cross-coupling reaction of the corresponding flavone triflate [17]. However, there has been no example of diphenylamine type flavones in the literature yet. 7-Amino-5-hidroxymethylfalvone could be synthesized via a five-step procedure from 3,5-dimethoxyaniline with a yield of 31% [16]. Chrysin (**1**) could be modified to 7-amino-5-hidroxyflavone via a four-step procedure with a 34% yield [17]. In these cases, subsequent arylation is necessary to obtain the corresponding biaryl structure. These lengthy and multistep procedures can be replaced by our reported method. Moreover, a 7-alkylamin derivative of chrysin (**1**) has been reported by Li et al. [6]. Their product was synthesized in six steps via a SNAr reaction from the corresponding tosylate, with a total yield of 45%.

## 2. Results and Discussion

### 2.1. Chemistry

**Scheme 1:**
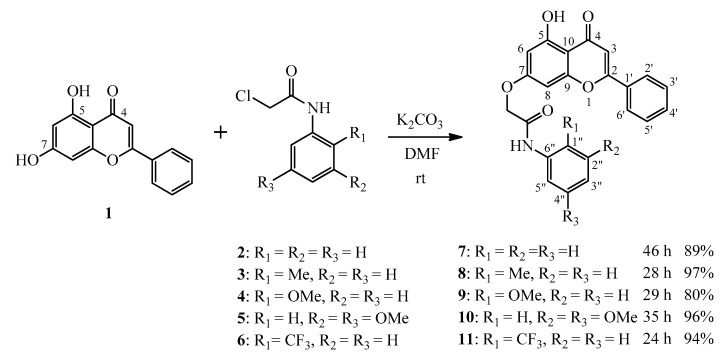
*O*-alkylation reaction of chrysin (**1**) in position 7 with *N*-phenylchloroacetamides (**2**–**6**) at room temperature.

The first task was the selective *O*-alkylation of chrysin (**1**) at position 7 with various *N*-phenylchloroacetamides, such as 2-chloro-*N*-phenylacetamide (**2**), 2-chloro-*N*-*o*-tolylacetamide (**3**), 2-chloro-*N*-(2-methoxyphenyl)acetamide (**4**), 2-chloro-*N*-(3,5-dimethoxyphenyl)acetamide (**5**), as well as 2-chloro-*N*-(2-(trifluoromethyl)phenyl)acetamide (**6**). The “driving force” of this work was that compounds with a similar structure had previously been shown to have antitumor activity on a resistant ABL (T315I) cell line [18]. The alkylation reactions were performed in DMF using potassium carbonate as the base at room temperature (Scheme 1). The targeted acetamide derivatives (**7**–**11**) have been successfully synthesized in high yields (80–97%). The regioselectivity of the alkylation reaction is assumed to be caused by the intramolecular H-bond between the oxo group at position 4 and the hydroxy group at position 5.

**Scheme 2:**
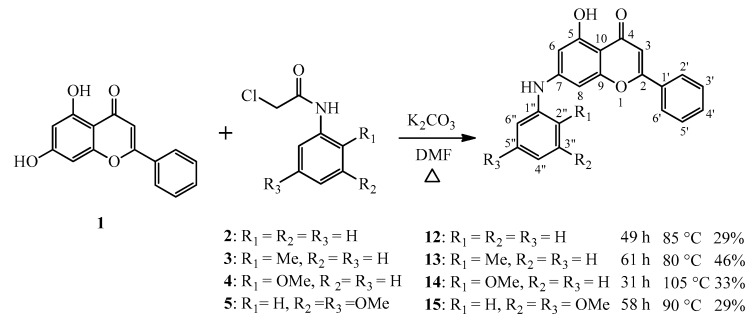
Reaction of chrysin (**1**) with *N*-phenylchloroacetamides (**2**–**5**) at a temperature of 80–105 °C. A new method for the synthesis of 7-aminochrysin derivatives (**12**–**15**).

The reaction of **1** with chloroacetamides **2**–**5** at a temperature of 80–105 °C gave diphenylamine-type 7-aminochrysin derivatives (**12**–**15**) (Scheme 2).

The mechanism for the formation of products **12**–**15** can be explained by a consecutive reaction sequence in which the first step is the *O*-alkylation shown above, followed by a one-pot Smiles rearrangement [19,20,21] at elevated temperature, and a final hydrolysis (see Scheme 5). The procedure produces the corresponding aminoflavone derivatives. Hydrolysis may take place due to the water content of the DMF solvent, or due to the water molecules formed from the thermal decomposition of the formal H_2_CO_3_ molecule that is the side product in the *O*-alkylation reaction.

After the consecutive reaction sequence, the yields of the products (**12**–**15**) were low, as at the applied temperature the alkylation was not fully regioselective, and a bis-alkylated product was also formed that was identified by HPLC-MS. The temperature required depended on the electronic properties of the aromatic system of acetamide; in particular, electron-withdrawing substituents facilitated the deprotonation.

**Scheme 3:**
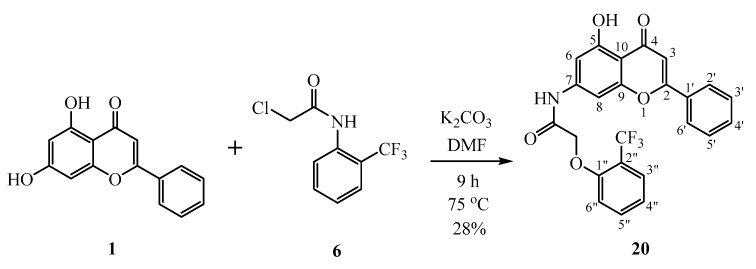
The reaction between chrysin (**1**) and 2-chloro-*N*-(2-(trifluoromethyl)phenyl)acetamide (**6**) at 75 °C, leading to the unexpected product **20**.

When the 2-chloro-*N*-(2-(trifluoromethyl)phenyl)acetamide (**6**) was the alkylating agent, an unexpected compound, a chromenyl phenoxyacetamide derivative (**20**), could be isolated (Scheme 3).

Although the ^1^H NMR spectrum of **20** resembled that of the expected compound **11** (Scheme 1), some protons had significantly different chemical shifts. In the chrysin skeleton, the resonances of the H-6 and H-8 protons of **20** were ca. 0.5 ppm downfield, and the resonances of the H-4″ and H-6″ protons of **20** were ca. 0.3 ppm upfield as compared to the same resonances of **11**.

The structure of **20** was eventually elucidated using ^1^H NMR, ^13^C NMR, COSY (^1^H-^1^H correlation spectroscopy), HSQC (^1^H-^13^C heteronuclear single quantum coherence), HMBC (^1^H-^13^C heteronuclear multiple bond correlation), and NOESY (^1^H-^1^H nuclear Overhauser effect spectroscopy).

**Figure 2:**
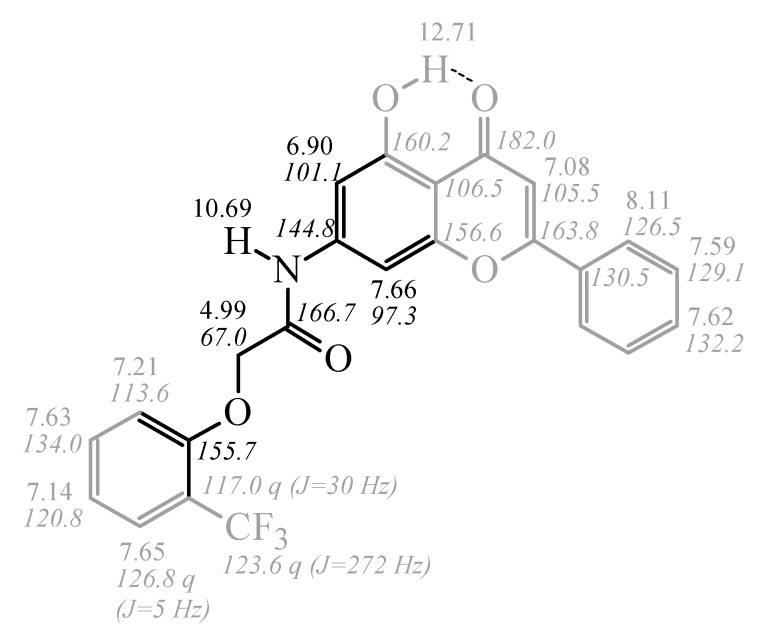
^1^H and ^13^C NMR chemical shift (given in ppm) assignments for compound **20**.

The assignment of the parts of the structure shown in gray (Figure 2) was first achieved using COSY (to establish the constitutional connections between the aromatic CHs), HSQC (to correlate the ^1^H NMR resonances with the ^13^C NMR resonances), and HMBC (to correlate protons and ^13^C atoms that are 2, 3 or 4 bonds apart). In the 2-(trifluoromethyl)phenyl moiety, ^13^C resonances (123.6 ppm, 117.0 ppm, and 126.8 ppm) were assigned on the basis of their multiplicity and the corresponding coupling constants (^1^*J*_F,C_ = 272 Hz, ^2^*J*_F,C_ = 30 Hz, ^3^*J*_F,C_ = 5 Hz) in the ^13^C NMR spectrum.

HMBC correlations ^1^H: 7.63 ppm, ^13^C: 155.7 ppm and ^1^H: 7.21 ppm, ^13^C: 155.7 ppm allow us to assign the sixth carbon atom in the benzene ring. Its chemical shift is more downfield than expected for an NH-aryl function, and the value of 155.7 ppm indicates an *O*-aryl function. The position of the CH_2_ group was identified by the three-bond (strong) HMBC correlation ^1^H: 4.99 ppm, ^13^C: 155.7 ppm, and by the fact that the chemical shift of the CH_2_ carbon atom is 67.0 ppm, indicating an oxygen atom being attached to the carbon. The carbonyl group was identified by a two-bond HMBC correlation (^1^H: 4.99 ppm, ^13^C: 166.7 ppm), the ^1^H NMR multiplicity of the OCH_2_ group (singlet means a quaternary carbon atom at the other side), and the absence of HMBC correlations for the proton signal at 4.99 ppm to any carbon atom of the chrysin moiety. The NH proton at 10.69 ppm gives HMBC correlations to the carbon atoms at 101.1 ppm (strong, three-bond, chrysin C6), 97.3 ppm (strong, three-bond, chrysin C8), 166.7 ppm (strong, two-bond correlation to a carbonyl carbon atom), 144.8 ppm (weak, two-bond, chrysin C7) and 156.6 (weak, four-bond), indicating its direct connection to the chrysin moiety. The structure was supported by the NOESY correlation between the protons resonating at 10.69 ppm and 6.90 ppm, showing their spatial proximity, and the unusual downfield chemical shift of chrysin H8 proton (7.66 ppm, cf. 6.5–7 ppm in most chrysin derivatives), indicating the deshielding effect of the nearby C=O group (Figure 2).

**Scheme 4:**
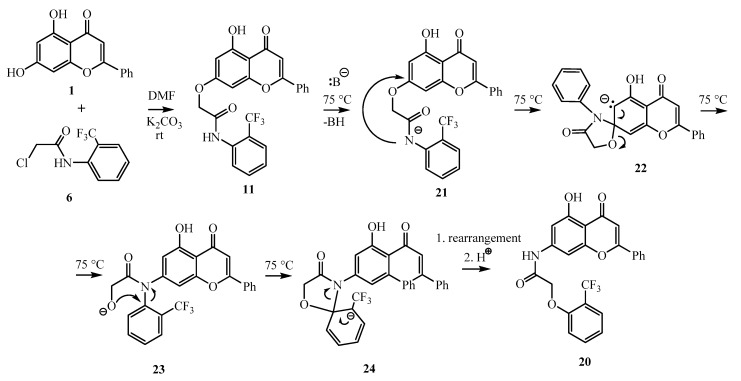
Proposed mechanism for the formation of **20**.

It is assumed that compound **11**, formed by *O*-alkylation, undergoes a Smiles rearrangement. However, presumably due to the electron-withdrawing effect of the trifluoromethyl group, not a hydrolysis, but a second Smiles rearrangement takes place starting from the intermediate **23** (Scheme 4). The resulting **24**, containing a spiro-oxazolidinone derivative, undergoes a ring-opening and a protonation step to produce compound **20**.

### 2.2. Kinetic and Mechanistic Investigation of the Reaction 1 + 4 → 9 + 14

**Figure 3:**
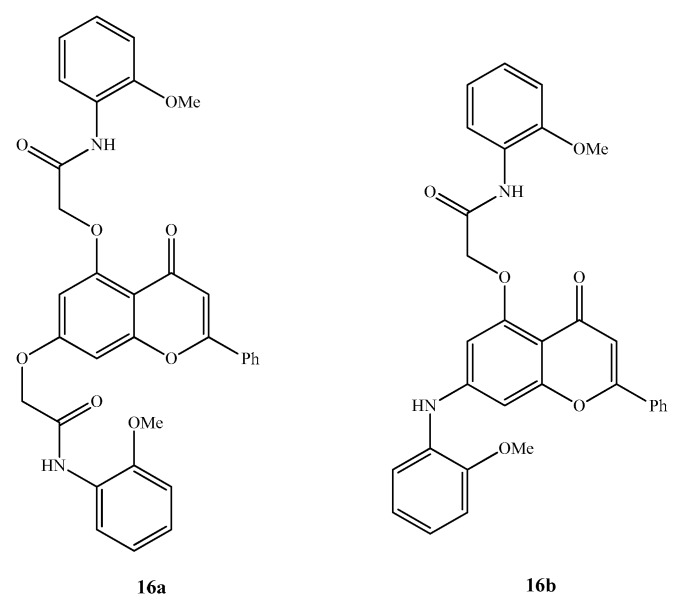
Side products of the reaction between **1** and **4**.

The alkylation of chrysin (**1**) with 2-chloro-*N*-(2-methoxyphenyl)acetamide (**4**) at room temperature resulted in compound **9** with an 80% yield after 29 h. Performing the reaction at 105 °C, diphenylamine **14** could be isolated at 33% after 31 h. The latter reaction was investigated in an HPLC-MS system by taking samples from the reaction after 0.25 h, 0.5 h, 1 h, 2 h, 24 h, and 48 h (Table 1). The corresponding conversions have been calculated from the Total Ion Chromatogram (TIC). It turned out that, after 15 min, 70% of the chrysin (**1**) and **4** chloroacetamide formed intermediate **9** (entry 2), and the reaction showed practically full conversion after 30 min (entry 3). As a side product, bis alkylated chrysin (**16a**) can be detected at 4% after 0.5 h, and the amount of **16a** reaches 10% after 1 h (entries 3 and 4) (Figure 3). Notably, the amount of **9** does not change in the first 2 h of the reaction (entry 5), but after 24 h it starts to decrease to 61% (entry 6) and finally reaches 43% after 48 h (entry 7). Meanwhile, 24 h is necessary for obtaining a 19% conversion for the rearranged product **14** (entry 6), which increases to 31% after 48 h (entry 7). The amount of the doubly alkylated **16a** is somewhat lower after 24 h and 48 h, and during this time period the formation of the Smiles-rearranged bis alkylated **16b** is detected (entries 6 and 7).

The mechanisms of the alkylation and the Smiles rearrangement have been investigated by DFT level (B3LYP/6-31G (d,p), considering the solvent effect of DMF using the IEFPCM solvent model [22,23,24,25]) quantum chemical computations using the Gaussian 09 program package (Wallingford, CT, USA) [26].

**Scheme 5:**
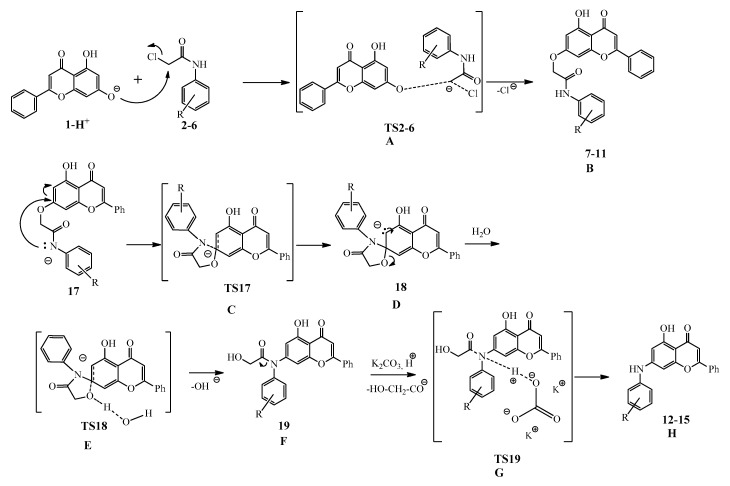
Computed mechanistic steps for the alkylation of chrysin (**1**) and the following Smiles rearrangement.

First, the alkylation reaction of **1** with chloroacetamides **2**–**6** was investigated. Experiments indicated that, for full conversion and high yields, a reaction time of 24–46 h is necessary. The nucleophilic substitution was modeled with the appropriately deprotonated chrysin (**1 − H^+^**) attacking the carbon atom of the chloroacetamide, forcing the chlorine to leave (Scheme 5). The Gibbs free energies for this transformation have been obtained in a range of 67.6–75.4 kJ mol^−1^ for each reagent, suggesting no significant influence of the substituents on the phenyl ring of the chloroacetamides (Table 2, column A). The reactions in all cases had endothermic characteristics, with formation energies ranging from −67.1 kJ mol^−1^ to −73.6 kJ mol^−1^ (Table 2, column B). Although the Smiles rearrangement of these adducts required slightly different experimental conditions—in particular, a temperature range of 75–105 °C for 31–61 h—the corresponding yields did not differ very much. Regarding the computational results, one can see that the first ring-closing step of the rearrangement is more advantageous for the methyl- and methoxy-substituted derivatives (**13**,**14**), and this difference can be seen for the next steps as well. The following deacetylation is exothermic; this is the rate-determining step with a 127.0–155.8 kJ mol^−1^ high transition state energy, which is the highest for the CF_3_-substituted one. Although the formation of the final product is endothermic in all cases, there are endothermic intermediate steps found for every compound except the OMe-substituted one. This observation and the large activation energy for the last step might explain the long reaction time at elevated temperature necessary for the full conversion.

In addition to the transformations presented above, the second Smiles rearrangement explaining the formation of **20** has been investigated theoretically as well. The **23**→**24** transformation (see Scheme 4) requires a Gibbs free energy investment of only 6.4 kJ mol^−1^, and is slightly endothermic (ΔG^0^ = −7.5 kJ mol^−1^). The subsequent ring opening of **24**, resulting in the deprotonated form of **20**, has a very low TS (ΔG^#^ = 3.6 kJ mol^−1^), and the driving force of the rearrangement is explained by the large energy gain from this final step (ΔG^0^ = −87.0 kJ mol^−1^). 

### 2.3. Biological Evaluation

The antiproliferative activities of the 10 synthesized compounds (**7**–**15** and **20**) were examined against 60 human tumor cell lines (NCI60), representing leukemia, non-small-cell lung cancer, colon cancer, CNS cancer, melanoma, ovarian cancer, renal cancer, prostate cancer, and breast cancer, respectively, at the National Cancer Institute (NCI, Bethesda, MD, USA) [27,28,29,30,31].

The screening results are given in Table 3, where the biological activities were determined at a concentration of 10^−5^ M. The percentages of growth show the amount of living cancer cells compared to a reference. The negative numbers indicate a significant decrease in the cell number. A notable antiproliferative effect was not shown by chrysin (**1**) and compounds **7**–**10**. The transformation of *N*-phenylacetamides to the corresponding diphenylamine structures (**13**–**15**) has drastically increased the antitumor activity, except in the case of compound **12**, which was ineffective on the investigated cell lines. This points to the fact that the substitution of the aromatic ring of the *N*-phenylacetamide is important for the anticancer activity. The promising result showed by compound **11** in comparison to the derivatives with the same structure (**7**–**10**) is surprising. Inverting the spacer between the two aromatic rings (see compound **20**) interfered with its antineoplastic effect, which might be explained by the displacement of hydrogen bond acceptors and donors.

Since compounds **11**, **13**, **14**, and **15** had shown significant antiproliferative effect on several cancer cell lines during the one-dose test, they were subjected to a five-dose screening. The GI_50_ (50% growth inhibition), TGI (total growth inhibition), and LC_50_ (50% loss of cells) values are given in Table 4. The results show that the antineoplastic effect of the latter four compounds (**11**, **13**, **14**, and **15**) is significantly increased as compared to the ineffective chrysin (**1**). Micromolar GI_50_ values were determined in the case of compounds **11**, **13**, and **14**. The most effective species, compound 15, gave submicromolar GI_50_ values. This experience is greatly promising for the further development of the presented biphenylamine structure. Compound **15** showed the highest cytotoxic activity on the MCF7 cell line of breast cancer (GI_50_ = 30 nM) and on the HCT-15 cell line of colon cancer (GI_50_ = 60 nM).

The results obtained in this study are encouraging for the future optimization of the compounds; in particular, with the involvement of other chloroacetamides, a more complete SAR could be obtained. It is noted that during this research the starting compounds for the hit molecules could be prepared from easily and cheaply available starting materials, thus further optimization studies would be feasible. In addition, we would like to emphasize that this study describing the anticancer effect of the set of compounds may be the starting point for more systematic and detailed research that could result in a larger number of candidates.

## 3. Materials and Methods

### 3.1. General Materials and Methods

All chemicals were purchased from Sigma-Aldrich (Budapest, Hungary) and were used as received. Melting points were measured on a VEB Analytik Dresden PHMK-77/1328 apparatus (Dresden, Germany) and are uncorrected. IR spectra were recorded on Zeiss IR 75 and 80 instruments (Thornwood, NY, USA). NMR measurements were performed on a Bruker Avance III HDX 400 MHz NMR spectrometer equipped with a ^31^P–^15^N{^1^H–^19^F} 5 mm CryoProbe Prodigy BBO probe, a Bruker Avance III HDX 500 MHz NMR spectrometer equipped with a ^1^H{^13^C/^15^N} 5 mm TCI CryoProbe, and a Bruker Avance III HDX 800 MHz NMR spectrometer equipped with a ^1^H–^19^F{^13^C/^15^N} 5 mm TCI CryoProbe (Bruker Corporation, Billerica, MA, USA). ^1^H And ^13^C chemical shifts are given on the delta scale as parts per million (ppm) relative to tetramethylsilane. One-dimensional ^1^H, ^13^C, and ^19^F spectra and two-dimensional ^1^H–^1^H COSY, ^1^H–^1^H NOESY, ^1^H–^13^C HSQC, and ^1^H–^13^C HMBC spectra were acquired using pulse sequences included in the standard spectrometer software package (Bruker TopSpin 3.5, Bruker Corporation). ESI-HRMS and MS-MS analyses were performed on a Thermo Velos Pro Orbitrap Elite (Thermo Fisher Scientific, Bremen, Germany) system. The ionization method was ESI, operated in positive ion mode. The protonated molecular ion peaks were fragmented by CID (collision-induced dissociation) at a normalized collision energy of 35–65%. For the CID experiment, helium was used as the collision gas. The samples were dissolved in methanol. Data acquisition and analysis were accomplished with Xcalibur software version 2.0 (Thermo Fisher Scientific). EI-HRMS analyses were performed on a Thermo Q Exactive GC Orbitrap (Thermo Fisher Scientific, Bremen, Germany) system. The ionization method was EI and operated in positive ion mode. Electron energy was 70 eV and the source temperature was set at 250 °C. Data acquisition and analysis were accomplished with Xcalibur software version 4.0 (Thermo Fisher Scientific). TLC was carried out using DC-Alufolien Kieselgel 60 F_254_ (Merck, Budapest, Hungary) plates. Preparative TLC analyses were performed on silica gel 60 PF_254+366_ (Merck) glass plates.

### 3.2. In Silico Studies on the Smiles Rearrangement

The DFT level computations at the B3LYP/6-31G (d,p) level were performed considering the solvent effect of DMF using the IEFPCM solvent model [22,23,24,25] with the Gaussian 09 program package [26]. The geometries of the molecules were optimized in all cases, and frequency calculations were also performed that resulted in the energy values presented in Table S1 and used in the figures of the manuscript. The solution phase Gibbs free energies were obtained by frequency calculations as well. The H, G, and S values obtained are given at standard conditions, and the corrected total energies of the molecules were taken into account. Entropic and thermal corrections are evaluated for isolated molecules using standard rigid rotor harmonic oscillator approximations. (Put another way, the Gibbs free energy is taken as the “sum of electronic and thermal free energies” printed in a Gaussian 09 vibrational frequency calculation). Standard state correction was taken into account. The transition states were optimized with the QST3 or the TS (Berny) method. Transition states were identified by having one imaginary frequency in the Hessian matrix, and IRC calculations [32] were performed in order to prove that the transition states connect two corresponding minima. All the geometries and transition states were optimized, and frequency calculations were made to assure that the structures are in a local minimum or in a saddle point, respectively.

### 3.3. Biological Evaluation

The in vitro antiproliferative activity of the synthesized compounds (**7**–**16**) was assessed by the protocols of the NCI (USA), as described in the references [27,28,29,30,31].

#### 3.3.1. One-Dose Screen

All compounds were tested initially at a single high dose (10^−5^ M) in the full NCI60 cell panel. The number reported for the one-dose assay is growth relative to the no-drug control, and relative to the time zero number of cells. This allowed the detection of both growth inhibition (values between 0 and 100) and lethality (values less than 0). For example, a value of 100 means no growth inhibition. A value of 40 would mean 60% growth inhibition. A value of 0 means no net growth over the course of the experiment. A value of −40 would mean 40% lethality. A value of −100 means all cells are dead.

#### 3.3.2. Five-Dose Screen

Compounds that exhibited significant growth inhibition in the one-dose screen were evaluated against the 60-cell panel at five concentration levels. The human tumor cell lines of the cancer screening panel were grown in RPMI 1640 medium containing 5% fetal bovine serum and 2 mM l-glutamine. Typically, cells were inoculated in 96-well microtiter plates in 100 μL at plating densities ranging from 5000 to 40,000 cells/well depending on the doubling time of individual cell lines. After cell inoculation, the microtiter plates were incubated at 37 °C, 5% CO_2_, 95% air, and 100% relative humidity for 24 h prior to the addition of experimental drugs. After 24 h, two plates of each cell line were fixed in situ with trichloroacetic acid (TCA), to represent a measurement of the cell population for each cell line at the time of drug addition (*t_z_*). Experimental drugs were solubilized in dimethyl sulfoxide at 400-fold the desired final maximum test concentration and stored frozen prior to use. At the time of drug addition, an aliquot of frozen concentrate was thawed and diluted to twice the desired final maximum test concentration with complete medium containing 50 μg ml^−1^ gentamicin. Additional four, 10-fold or ½ log serial dilutions were made to provide a total of five drug concentrations plus control. Aliquots of 100 μL of these different drug dilutions were added to the appropriate microtiter wells already containing 100 μL of medium, resulting in the required final drug concentrations.

Following drug addition, the plates were incubated at 37 °C, 5% CO_2_, 95% air, and 100% relative humidity for an additional 48 h. For adherent cells, the assay was terminated by the addition of cold TCA. Cells were fixed in situ by addition of 50 μL of cold 50% (*w*/*v*) TCA, and incubated at 4 °C for 60 min. The supernatant was discarded, and the plates were washed with water (5×) and dried in air. Sulforhodamine B (SRB) solution (100 μL) at 0.4% (*w*/*v*) in 1% acetic acid was added to each well, and plates are incubated at room temperature for 10 min. After staining, unbound dye is removed by washing five times with 1% acetic acid and the plates were dried in air. Bound stain is subsequently solubilized with 10 mM trizma base, and the absorbance is read on an automated plate reader at λ = 515 nm. Using the seven absorbance measurements [time zero (*t_z_*), control growth (*c*), and test growth in the presence of drug at the five concentration levels (*t_i_*)], the percentage growth was calculated at each of the drug concentrations levels. Growth inhibition (%) was calculated as:

[(*t_i_* − *t_z_*)/(*c* − *t_z_*)] × 100, for concentrations where *t_i_* ≥ *t_z_* (1)

[(*t_i_* − *t_z_*)/(*t_z_*)] × 100, for concentrations where *t_i_* < *t_z_.* (2)

Three dose‒response parameters were calculated as follows. GI_50_ (growth inhibition of 50%) was calculated from Equation (3), which is the drug concentration resulting in a 50% reduction in the net protein increase (as measured by SRB staining) in control cells during the drug incubation. The drug concentration resulting in total growth inhibition (TGI) was calculated from Equation (4), where *t_i_* = *t_z_*. The LC_50_ indicating a 50% net loss of cells following treatment was calculated from Equation (5):

[(*t_i_* − *t_z_*)/(*c* − *t_z_*)] × 100 = 50 (3)

[(*t_i_* − *t_z_*)/(*c* − *t_z_*)] × 100 = 0 (4)

[(*t_i_* − *t_z_*)/(*t_z_*)] × 100 = −50. (5)

## 4. Conclusions

Several new compounds were synthesized from the naturally occurring chrysin (**1**). In the first step, chrysin (**1**) was modified at position 7 via a regioselective alkylation then, a novel method was applied to form the desired 7-aminochrysin derivatives. This protocol might be applicable to the synthesis of other aminoflavone derivatives. An in silico investigation was performed to evaluate the mechanism of the transformation. It was found that the primary alkylation is followed by a Smiles rearrangement, and a hydrolysis. However, when the acetamide derivative used for alkylation contains an electron-withdrawing substituent (as in compound **6**), the Smiles rearrangement is followed by a second nucleophilic aromatic substitution. During the synthetic work we also came across an unexpected product (**20**) whose structure was elucidated with high confidence by a comprehensive, nonroutine NMR analysis. The antiproliferative activity of the synthesized compounds was investigated at the National Cancer Institute (USA) in vitro. A few compounds showed encouraging anticancer effect as compared to chrysin (**1**). The most potent derivative (**15**) exhibited nanomolar antitumor activity on the MCF7 cell line of breast cancer (GI_50_ = 30 nM) and on the HCT-15 cell line of colon cancer (GI_50_ = 60 nM).

## Figures and Tables

**Table 1:** Conversion values measured by HPLC-MS based on the TIC.

Entry	Time (h)	Conversion (MS, %) of Compounds with the Corresponding M + H^+^ in Brackets					
		1 (255)	4 (200)	9 (418)	14 (360)	16a (581)	16b (523)
1	0	100	100	n.d.	n.d.	n.d.	n.d.
2	0.25	29	27	61	n.d.	n.d.	n.d.
3	0.5	5	10	100	n.d.	4	n.d.
4	1	n.d.	7	96	n.d.	10	n.d.
5	2	n.d.	7	99	n.d.	10	n.d.
6	24	n.d.	5	61	19	9	2
7	48	n.d.	2	43	31	8	4

n.d.—not detected in the chromatogram.

**Table 2:** Energetics for the alkylation of chrysin (**1**) and the following Smiles rearrangement. ΔG^#^ [kJ mol^−1^] refers for the activation Gibbs free energy computed from the differences of the transition states (**TS2**–**6**) and the single reactants. ΔG^0^ [kJ mol^−1^] refers to the formation Gibbs free energy of products **7**–**11** from the single reactants.

Column				A	B	C	B	E	F	G	H
Entry	R_1_	R_2_	R_3_	ΔG^#^ TS2–6	ΔG^0^ 7–11	ΔG^#^ TS17	ΔG^0^ 18	ΔG^#^ TS18	ΔG^0^ 19	ΔG^#^ TS19	ΔG^0^ 12–16
1	H	H	H	73.3	−67.2	31.0	16.1	34.9	9.9	140.7	−7.0
2	Me	H	H	67.6	−73.0	19.8	2.1	20.6	−6.5	137.0	−13.3
3	OMe	H	H	69.3	−73.6	16.0	−4.1	20.4	−7.4	127.0	−28.6
4	H	OMe	OMe	75.4	−67.1	33.5	19.8	35.6	17.1	143.6	−4.2
5	CF_3_	H	H	73.3	−71.0	32.7	25.6	42.9	17.6	155.8	−9.0

**Table 3:** Antiproliferative activities of chrysin (**1**) and compounds **7**–**15** and **20** against 60 human cancer cell lines in vitro. The negative numbers causing cell death are highlighted in bold.

	1	7	8	9	10	11	12	13	14	15	20
Leukemia			Living Cells, %								
CCRF-CEM	102.24	94.38	88.46	103.61	100.26	58.61	99.72	29.51	7.29	10.80	93.43
HL-60(TB)	116.20	74.53	80.72	96.19	86.01	55.03	95.29	8.58	**−6.07**	**−35.66**	96.64
K-562	96.80	95.30	84.14	102.18	95.93	49.21	70.67	10.23	11.56	13.10	93.28
MOLT-4	105.93	91.11	86.70	101.33	84.14	52.14	102.90	25.82	25.94	24.60	98.42
RPMI-8226	101.04	93.25	100.27	103.24	101.95	54.84	103.51	29.70	16.42	17.06	90.20
SR	77.87	n.d.	n.d.	85.32	n.d.	n.d.	40.93	7.93	5.75	17.29	n.d.
Non-Small-Cell Lung Cancer											
A549/ATCC	98.46	84.59	89.91	99.07	93.60	0.21	88.14	35.17	33.70	25.89	86.93
EKVX	89.35	102.66	95.38	100.69	104.69	51.40	95.16	35.54	57.37	38.16	100.07
HOP-62	113.09	100.81	96.03	100.66	94.26	16.24	102.52	49.44	35.64	38.50	96.92
HOP-92	77.93	89.87	94.70	122.42	90.77	51.61	97.26	86.73	62.17	79.55	92.47
NCI-H226	86.84	n.d.	n.d.	96.59	n.d.	n.d.	82.23	57.82	76.84	67.67	n.d.
NCI-H23	92.57	83.67	98.22	99.50	94.72	46.10	96.33	45.45	51.50	35.95	88.56
NCI-H233M	98.30	85.60	95.63	100.67	98.57	62.22	98.48	70.48	80.08	44.07	93.82
NCI-H460	98.34	61.56	79.30	83.22	97.66	**−42.49**	89.31	8.06	11.44	7.62	91.59
NCI-H552	88.95	81.54	84.78	95.78	88.34	46.64	87.19	35.99	7.64	**−35.25**	82.91
Colon Cancer											
COLO 205	104.94	108.40	108.85	117.93	109.55	60.11	117.09	61.77	76.85	48.45	106.47
HCC-2998	102.88	99.66	104.54	102.54	97.53	89.85	98.67	76.72	71.93	32.39	106.49
HCT-116	82.69	57.38	66.46	71.54	97.57	5.84	94.22	15.22	14.09	12.96	84.06
HCT-15	90.99	96.02	97.82	110.39	101.89	53.37	88.34	17.62	29.25	25.00	102.90
HT29	102.89	104.70	109.72	104.51	98.93	70.62	102.62	69.86	16.96	3.58	108.92
KM12	92.93	86.55	100.16	104.94	103.34	68.50	98.53	36.41	36.18	20.97	93.03
SW-620	101.60	87.53	91.85	101.53	96.14	21.80	92.66	21.04	20.80	24.89	92.57
CNS Cancer											
SF-268	101.55	90.96	92.88	103.44	95.94	63.49	108.82	61.47	44.88	48.20	93.54
SF-295	99.86	100.68	105.53	107.28	101.39	34.55	94.93	27.12	25.87	33.51	99.41
SF-539	92.17	90.97	95.00	94.46	95.80	35.24	88.56	23.08	28.35	**−35.20**	101.29
SNB-19	86.04	87.38	97.95	98.29	94.84	45.17	80.85	32.62	43.92	43.08	93.88
SNB-75	88.98	88.03	89.86	98.11	85.65	**−4.06**	85.97	**−5.66**	**−4.79**	**−9.05**	84.74
U251	80.67	93.73	99.75	95.07	99.90	4.09	103.19	36.19	22.12	22.78	105.43
Melanoma											
LOX IMVI	85.08	89.59	91.33	97.12	94.23	51.99	98.19	30.95	39.93	41.67	91.07
MALME-3M	101.76	97.72	99.19	106.86	96.36	54.32	99.49	47.94	44.12	73.59	96.86
M14	106.78	85.43	95.69	107.85	97.45	70.56	102.80	24.59	20.80	28.37	89.53
MDA-MB-435	99.53	95.35	100.50	101.33	100.05	78.05	91.47	1.90	**−24.29**	4.04	97.39
SK-MEL-2	109.90	96.58	106.20	110.49	101.95	92.04	118.37	65.66	13.67	13.19	102.47
SK-MEL-28	101.70	105.88	103.62	106.04	103.28	79.12	101.17	51.11	56.53	68.55	103.95
SK-MEL-5	92.85	98.99	97.12	96.32	n.d.	n.d.	98.19	23.46	24.55	28.62	n.d.
UACC-257	118.94	97.25	97.09	100.73	95.08	73.38	113.80	80.55	54.00	77.94	92.88
UACC-62	82.24	93.67	94.23	100.84	93.14	77.53	94.55	43.38	35.81	42.50	90.77
Ovarian Cancer											
IGROV1	95.22	99.14	94.90	99.66	97.11	40.29	101.69	45.33	51.38	42.09	99.29
OVCAR-3	97.60	105.55	112.68	117.24	116.11	**−2.06**	119.29	**−4.27**	5.90	0.37	107.74
OVCAR-4	112.07	78.90	92.03	108.84	101.20	**−50.15**	95.50	53.09	65.63	59.30	97.96
OVCAR-5	99.07	98.07	98.20	106.13	98.13	62.88	107.85	89.20	78.14	45.48	101.59
OVCAR-8	95.19	87.16	96.80	93.95	101.69	29.02	76.29	52.50	32.37	26.14	95.43
NCI/ADR-RES	92.84	88.88	98.42	98.91	96.78	51.21	84.49	4.90	11.48	22.21	93.45
SK-OV-3	128.15	106.68	112.10	99.01	106.94	44.14	91.61	59.29	50.68	35.94	101.19
Renal Cancer											
786-0	99.20	81.89	102.88	103.24	98.94	7.11	99.45	43.46	49.17	45.40	102.34
A498	86.62	98.75	107.57	114.05	103.91	55.93	86.89	31.26	11.62	**−4.73**	89.70
ACHN	85.03	57.89	89.02	95.66	100.57	17.85	88.95	34.50	50.84	48.27	90.18
CAKI-1	83.56	77.85	85.07	94.67	83.96	22.26	78.85	39.00	41.29	47.20	84.52
RXF 393	91.19	98.26	105.07	114.29	103.82	63.39	87.18	27.97	n.d.	n.d.	96.03
SN12C	85.77	89.89	100.73	93.53	96.93	27.73	89.88	31.78	36.59	44.31	94.21
TK-10	107.21	102.52	101.40	107.48	96.05	**−13.91**	115.18	110.08	66.29	43.97	104.00
UO-31	89.42	80.20	80.65	81.08	84.04	17.07	113.09	42.28	46.60	40.33	88.09
Prostate Cancer											
PC-3	93.17	86.21	86.03	102.30	96.04	64.61	91.31	40.61	54.48	27.81	90.42
DU-145	92.00	81.47	83.97	95.26	97.68	52.73	93.71	26.85	45.58	19.97	99.56
Breast Cancer											
MCF7	103.05	85.34	84.36	89.90	93.57	49.76	88.89	15.46	17.34	23.46	89.40
MDA-MB-231/ATCC	82.64	72.92	87.92	95.07	99.54	9.62	97.20	50.66	39.19	15.88	83.19
HS 578T	92.51	80.27	94.59	97.60	98.24	15.48	86.96	44.73	45.59	12.36	95.30
BT-549	91.01	n.d.	n.d.	103.53	n.d.	n.d.	90.69	47.08	40.89	80.16	n.d.
T-47D	101.44	94.33	85.80	86.66	97.63	14.26	91.42	36.46	31.43	55.24	95.24
MDA-MB-468	91.09	102.86	100.21	114.94	101.67	67.58	86.66	**−2.12**	**−1.72**	11.66	99.78
Mean:	96.10	90.32	95.08	100.69	97.53	40.61	94.75	38.33	34.57	29.48	95.38
Delta:	18.23	32.94	28.62	29.15	13.57	90.76	53.82	43.99	58.86	65.14	12.47
Range:	50.28	51.02	46.22	50.88	32.15	142.19	78.36	115.74	104.37	115.82	26.01

**Table 4:** Cytotoxic activities (GI_50_, TGI and LC_50_) of compounds **11**, **13**, **14**, and **15** against 60 human cancer cell lines in vitro. Values where GI_50_ < 100 nM or TGI/LC_50_ < 10 μM are highlighted in bold.

	11			13			14			15		
Leukemia	GI_50_ (μM)	TGI (μM)	LC_50_ (μM)	GI_50_ (μM)	TGI (μM)	LC_50_ (μM)	GI_50_ (μM)	TGI (μM)	LC_50_ (μM)	GI_50_ (μM)	TGI (μM)	LC_50_ (μM)
CCRF-CEM	19.6	64.1	>100	2.25	>100	>100	3.57	>100	>100	0.4	>100	>100
HL-60 (TB)	>100	>100	>100	2.16	>100	>100	2.79	34.9	>100	0.32	>100	>100
K-562	5.03	>100	>100	0.47	>100	>100	0.59	>100	>100	0.28	>100	>100
MOLT-4	5.46	>100	>100	2.66	>100	>100	4.08	>100	>100	0.56	>100	>100
RPMI-8226	7.03	>100	>100	2.87	>100	>100	4.51	>100	>100	0.39	>100	>100
SR	4.44	>100	>100	0.40	>100	>100	0.53	>100	>100	0.25	>100	>100
Non-Small-Cell Lung Cancer												
A549/ATCC	1.71	**3.75**	**8.23**	2.11	>100	>100	5.16	>100	>100	0.72	94.7	>100
EKVX	9.37	62.4	>100	3.35	>100	>100	4.83	**5.6**	>100	0.64	>100	>100
HOP-62	2.62	**8.19**	35.7	3.23	>100	>100	3.55	>100	>100	0.56	21.7	>100
HOP-92	2.13	**6.71**	90.1	3.51	>100	>100	3.76	23.7	>100	1.95	21.3	>100
NCI-H226	2.28	**6.48**	>100	4.11	>100	>100	4.38	28.8	>100	1.25	71.2	>100
NCI-H23	3.07	11.6	49.6	4.75	>100	>100	4.22	52.6	>100	0.93	28	>100
NCI-H233M	>100	>100	>100	n.d.	>100	>100	5.98	27.4	>100	0.52	49.2	>100
NCI-H460	2.59	6.68	>100	2.89	>100	>100	3.37	12	>100	0.36	12.5	93.6
NCI-H552	6.77	>100	>100	1.79	>100	>100	2.67	27.1	>100	0.24	21.5	>100
Colon Cancer												
COLO 205	8.04	>100	>100	n.d.	>100	>100	9.92	>100	>100	5.13	>100	>100
HCC-2998	>100	>100	>100	n.d.	>100	>100	6.07	>100	>100	1.94	29.1	>100
HCT-116	1.83	**4.06**	**8.99**	3.36	>100	>100	3.31	>100	>100	0.42	>100	>100
HCT-15	3.28	21.1	>100	0.68	>100	>100	1.65	>100	>100	**0.06**	>100	>100
HT29	6.48	>100	>100	3.25	>100	>100	3.64	>100	>100	3.18	13.6	>100
KM12	8.17	>100	>100	2.38	>100	>100	2.76	55.2	>100	0.2	15.3	>100
SW-620	3.56	>100	>100	0.73	>100	>100	2.5	>100	>100	0.35	>100	>100
CNS Cancer												
SF-268	4.94	26.1	>100	4.49	>100	>100	4.71	48.2	>100	0.6	31	>100
SF-295	2.94	**9.13**	32.6	2.36	>100	>100	2.98	13.2	43.2	0.3	**3.79**	99.3
SF-539	2.21	**5.50**	20.5	3.10	>100	>100	2.73	10.4	36.3	0.28	**1.25**	**18.2**
SNB-19	4.17	21.7	>100	2.98	>100	>100	2.99	15.7	>100	0.48	20.5	>100
SNB-75	1.65	**4.41**	14.9	n.d.	n.d.	n.d.	2.39	**8.5**	59.6	0.17	**0.64**	44.2
U251	2.01	**4.45**	**9.84**	3.04	>100	>100	3.63	>100	>100	0.41	16	>100
Melanoma												
LOX IMVI	4.74	22.6	>100	2.75	>100	>100	3.41	>100	>100	0.51	>100	>100
MALME-3M	3.17	>100	>100	2.43	>100	>100	2.13	11.9	57.6	0.63	>100	>100
M14	27.6	>100	>100	2.38	>100	>100	n.d.	n.d.	n.d.	0.5	>100	>100
MDA-MB-435	6.03	>100	>100	0.31	>100	>100	0.36	2.03	>100	0.1	**0.33**	>100
SK-MEL-2	15.1	38.0	95.7	2.44	>100	>100	5.62	34.2	>100	0.79	78.5	>100
SK-MEL-28	n.d.	>100	>100	n.d.	>100	>100	3.64	25.8	>100	2.78	>100	>100
SK-MEL-5	4.10	25.4	>100	2.39	>100	>100	3	**9.58**	42.4	0.17	3.25	>100
UACC-257	>100	>100	>100	n.d.	>100	>100	6.72	63.5	>100	>100	>100	>100
UACC-62	6.58	>100	>100	2.89	>100	>100	2.39	12.8	61.1	0.3	14	>100
Ovarian Cancer												
IGROV1	3.61	15.9	86.3	3.20	>100	>100	2.98	18.5	94.5	0.23	25.5	>100
OVCAR-3	2.12	**4.12**	**7.99**	1.72	**4.91**	>100	2.05	**5.2**	21.5	0.22	**1.59**	>100
OVCAR-4	2.45	5.94	>100	3.38	>100	>100	4.49	47.8	>100	8.9	41.5	>100
OVCAR-5	7.18	>100	>100	>100	>100	>100	4.82	27.3	>100	0.55	22.6	>100
OVCAR-8	3.67	3.21	>100	3.61	>100	>100	4.65	>100	>100	0.54	17.1	>100
NCI/ADR-RES	3.45	22.5	>100	1.28	>100	>100	1.99	18	>100	0.45	26.7	>100
SK-OV-3	14.9	50.7	>100	5.56	>100	>100	3.57	17.9	96.4	0.4	14.1	74.3
Renal Cancer												
786-0	2.01	**4.15**	**8.56**	4.42	>100	>100	5.15	27	>100	1.04	20.7	91.4
A498	4.05	17.8	65.2	2.48	n.d.	>100	3.08	13.9	65.8	0.34	14	75.9
ACHN	2.91	**9.63**	60.5	4.25	>100	>100	3.38	13	41.5	0.59	>100	>100
CAKI-1	3.74	18.7	78.2	2.77	>100	>100	4.24	20.3	73.5	0.28	15	80.3
RXF 393	2.21	**6.34**	30.1	2.45	**9.56**	>100	1.74	**5.34**	28.1	0.21	**0.79**	38.1
SN12C	4.02	>100	>100	4.05	>100	>100	3.5	17.6	94.5	0.79	60.4	>100
TK-10	1.88	**3.71**	**7.30**	n.d.	>100	>100	10.4	28.6	78.5	10.7	79.4	>100
UO-31	2.44	n.d.	>100	n.d.	>100	>100	3.57	21.5	>100	0.62	22.9	98.3
Prostate Cancer												
PC-3	5.75	>100	>100	3.30	>100	>100	5.13	41.6	>100	0.52	27.2	>100
DU-145	7.97	>100	>100	3.78	>100	>100	4.35	16.4	51.7	0.4	**2.14**	>100
Breast Cancer												
MCF7	7.40	>100	>100	0.82	>100	>100	1.63	>100	>100	0.03	>100	>100
MDA-MB-231/ATCC	2.04	**5.26**	22.6	3.58	>100	>100	3.96	18.4	68.2	1.05	22.4	>100
HS 578T	2.60	7.80	>100	2.46	>100	>100	3.61	32.6	>100	0.3	**2.45**	>100
BT-549	5.19	38.9	>100	4.03	>100	>100	4.96	37.2	>100	0.77	>100	>100
T-47D	2.54	n.d.	>100	2.96	>100	>100	4.36	>100	>100	0.21	>100	>100
MDA-MB-468	4.95	35.8	>100	0.38	3.03	>100	1.5	**6.42**	>100	0.13	**0.64**	>100

## Supplementary Materials

The following are available online at https://www.mdpi.com/1420-3049/25/4/888/s1, Table S1: Energy values obtained for the computation of the 1→2 transformation and the related dimerization. The E, ZPE, U, H and G values were computed using the B3LYP/6-31 (d,p) method and are given in Hartree.
