# Supplementary material for: Synthesis and In Vitro Anticancer Evaluation of Novel Chrysin and 7-Aminochrysin Derivatives
Source: Molecules. 2020 Feb 17;25(4):888. doi: 10.3390/molecules25040888 (PMC7070641; doi:10.3390/molecules25040888)
Supplement: Supplementary file 1 [file molecules-25-00888-s001.pdf]

*molecules*

MDPI

*Article*

# Synthesis and *In Vitro* Anticancer Evaluation of Novel Chrysin and 7-aminochrysin Derivatives

**Szabolcs Mayer[1], Péter Keglevich[1]*, Péter Ábrányi-Balogh[2]*, Áron Szigetvári[3], Miklós Dékány[3], Csaba Szántay Jr.[3] and László Hazai[1]**

1 Department of Organic Chemistry and Technology, Faculty of Chemical Technology and Biotechnology, Budapest University of Technology and Economics, H-1111 Budapest, Gellért tér 4., Hungary; mayer.szabolcs@mail.bme.hu (S. M.); hazai@mail.bme.hu (L.H)

2 Medicinal Chemistry Research Group, Institute of Organic Chemistry, Research Centre for Natural Sciences, H-1117 Budapest, Magyar tudósok krt. 2., Hungary

3 Spectroscopic Research Department, Gedeon Richter Plc., H-1475 Budapest 10, P. O. Box 27, Hungary; szigetvaria@richter.hu (Á. S.); m.dekany@richter.hu (M. D.) cs.szantay@richter.hu (C.S.J)

* Correspondence: pkeglevich@mail.bme.hu (P.K); abranyi-balogh.peter@ttk.hu (P.Á.-B.)

## Supplementary materials:

### S.1. Chemistry

#### S.1.1. 2-((5-Hydroxy-4-oxo-2-phenyl-4*H*-chromen-7-yl)oxy)-*N*-phenylacetamide (**7**)

Chrysin (**1**) (100 mg, 0.39 mmol), 2-chloro-*N*-phenylacetamide (**2**) (71 mg, 0.42 mmol) and anhydrous potassium carbonate (56 mg, 0.41 mmol) were dissolved in DMF (4 mL). The reaction mixture was stirred at room temperature for 46 h, then, it was poured into water (30 mL). After filtering off the precipitate, the filtrate was extracted with DCM (3 x 30 mL). The combined organic layer was dried over $MgSO_4$ and concentrated *in vacuo*. The filtered precipitation and the crude product obtained by extraction were purified separately with preparative TLC (DCM : MeOH = 60 : 1). Combining the pure products 135 mg (89 %) of compound **7** was isolated as a white solid. M.p.: 227-228 °C. TLC (DCM : MeOH = 60 : 1); $R_f$ = 0.41. IR (KBr) 3421, 3401, 1693, 1661, 1532, 1170, 831 cm$^{-1}$. $^{1}$H NMR (499,9 MHz; DMSO-*d*$_6$) δ (ppm) 4.88 (s; 2H; C(7)-OC<u>H</u>$_2$); 6.51 (d; *J* = 2.3 Hz; 1H; H-6); 6.90 (d; *J* = 2.3 Hz; 1H; H-8); 7.07 (s; 1H; H-3); 7.07–7.10 (m; 1H; H-4′′); 7.31–7.36 (m; 2H; H-3′′, H-5′′); 7.57–7.61 (m; 2H; H-3′, H-5′); 7.61–7.66 (m; 3H; H-4′, H-2′′, H-6′′); 8.09–8.13 (m; 2H; H-2′, H-6′); 10.16 (s; 1H; C(1′′)-N<u>H</u>); 12.84 (s; 1H; C(5)-O<u>H</u>). $^{13}$C NMR (125.7 MHz; DMSO-*d*$_6$) δ (ppm) 67.2 (C(7)-O<u>C</u>H$_2$); 93.6 (C-8); 98.6 (C-6); 105.2 (C-10); 105.3 (C-3); 119.5 (C-2′′, C-6′′); 123.7 (C-4′′); 126.4 (C-2′, C-6′); 128.7 (C-3′′, C-5′′); 129.1 (C-3′, C-5′); 130.5 (C-1′); 132.1 (C-4′); 138.2 (C-1′′); 157.1 (C-9); 161.1 (C-5); 163.5 (C-2); 163.8 (C-7); 165.5 (C(1′′)-NH-<u>C</u>O); 182.0 (C-4). EI-HRMS: M=387.11020 (delta=0.2 ppm; $C_{23}H_{17}O_5N$).

#### S.1.2. 2-((5-Hydroxy-4-oxo-2-phenyl-4*H*-chromen-7-yl)oxy)-*N*-(*o*-tolyl)acetamide (**8**)

Chrysin (**1**) (100 mg, 0.39 mmol), 2-chloro-*N*-(*o*-tolyl)acetamide (**3**) (76 mg, 0.41 mmol) and anhydrous potassium carbonate (56 mg, 0.41 mmol) were dissolved in DMF (4 mL). The reaction mixture was stirred at room temperature for 28 h, then, it was poured into water (30 mL). After filtering off the precipitate, the filtrate was extracted with DCM (3 x 30 mL). The combined organic layer was dried over $MgSO_4$ and concentrated *in vacuo*. The filtered precipitation and the crude product obtained by extraction were purified separately with preparative TLC (DCM : MeOH = 40 : 1). Combining the pure products 155 mg (98 %) of compound **8** was isolated as a white solid. M.p.: 236-237 °C. TLC (DCM : MeOH = 40 : 1); $R_f$ = 0.51. IR (KBr) 3422, 3059, 1690, 1661, 1358, 1171, 1044, 753 cm$^{-1}$. $^{1}$H NMR (499.9 MHz; DMSO-*d*$_6$) δ (ppm) 2.22 (s; 3H; C(2′′)-C<u>H</u>$_3$); 4.91 (s; 2H; C(7)-OC<u>H</u>$_2$); 6.53 (d; *J* = 2.1 Hz; 1H; H-6); 6.91 (d; *J* = 2.1 Hz; 1H; H-8); 7.08 (s; 1H; H-3); 7.10–7.15 (m; 1H; H-4′′); 7.17–7.21 (m; 1H; H-5′′); 7.22–7.26 (m; 1H; H-3′′); 7.40–7.43 (m; 1H; H-6′′); 7.58–7.62 (m; 2H; H-3′, H-

5′); 7.62–7.66 (m; 1H; H-4′); 8.09–8.13 (m; 2H; H-2′, H-6′); 9.60 (br s; 1H; C(1′′)-NH); 12.83 (br s; 1H; C(5)-OH). $^{13}$C NMR (125.7 MHz; DMSO-$d_6$) δ (ppm) 17.6 (C(2′′)-$CH_3$); 67.2 (C(7)-$OCH_2$); 93.6 (C-8); 98.7 (C-6); 105.2 (C-10); 105.4 (C-3); 125.2 (C-6′′); 125.6 (C-4′′); 126.0 (C-5′′); 126.4 (C-2′, C-6′); 129.1 (C-3′, C-5′); 130.3 (C-3′′); 130.5 (C-1′); 132.1 (C-4′, C-2′′); 135.3 (C-1′′); 157.1 (C-9); 161.1 (C-5); 163.5 (C-2); 163.7 (C-7); 165.7 (C(1′′)-NH-CO); 182.0 (C-4). HRMS: M-H=400.11924 (delta=0.49 ppm; $C_{24}H_{18}O_5N$). HR-ESI-MS-MS (CID=35%; rel. int. %): 342(61); 293(19); 253(100).

S.1.3. 2-((5-Hydroxy-4-oxo-2-phenyl-4*H*-chromen-7-yl)oxy)-*N*-(2-methoxyphenyl)acetamide (**9**)

Chrysin (**1**) (50 mg, 0.20 mmol), 2-chloro-*N*-(2-methoxyphenyl)acetamide (**4**) (41 mg, 0.21 mmol) and anhydrous potassium carbonate (28 mg, 0.21 mmol) were dissolved in DMF (2 mL). The reaction mixture was stirred at room temperature for 29 h, then, it was poured into water (15 mL). After filtering off the precipitate, the filtrate was extracted with DCM (3 x 15 mL). The combined organic layer was dried over $MgSO_4$ and concentrated *in vacuo*. The filtered precipitation and the crude product obtained by extraction were purified separately with preparative TLC (DCM : MeOH = 40 : 1). Combining the pure products 66 mg (80 %) of compound **9** was isolated as a white solid. M.p.: 214-215 °C. TLC (DCM : MeOH = 40 : 1); $R_f$ = 0.43. IR (KBr) 3403, 3075, 1660, 1450, 1338, 1171, 832, 498 cm$^{-1}$. $^1$H NMR (799.7 MHz; $CDCl_3$) δ (ppm) 3.91 (s; 3H; C(2′′)-$OCH_3$); 4.71 (s; 2H; C(7)-$OCH_2$); 6.51 (d; *J* = 2.3 Hz; 1H; H-6); 6.60 (d; *J* = 2.3 Hz; 1H; H-8); 6.71 (s; 1H; H-3); 6.91 (dd; *J* = 8.2, 1.2 Hz; 1H; H-3′′); 6.99 (ddd; *J* = 8.0, 7.5, 1.2 Hz; 1H; H-5′′); 7.10 (ddd; *J* = 8.2, 7.5, 1.6 Hz; 1H; H-4′′); 7.53–7.56 (m; 2H; H-3′, H-5′); 7.56–7.59 (m; 1H; H-4′); 7.89–7.92 (m; 2H; H-2′, H-6′); 8.40 (dd; *J* = 8.0, 1.6 Hz; 1H; H-6′′); 8.87 (br s; C(1′′)-NH); 12.79 (s; 1H; C(5)-OH). $^{13}$C NMR (201.1 MHz; $CDCl_3$) δ (ppm) 55.9 (C(2′′)-$OCH_3$); 67.8 (C(7)-$OCH_2$); 92.9 (C-8); 99.4 (C-6); 106.1 (C-3); 106.7 (C-10); 110.1 (C-3′′); 119.9 (C-6′′); 121.1 (C-5′′); 124.6 (C-4′′); 126.4 (C-2′, C-6′); 126.5 (C-1′′); 129.2 (C-3′, C-5′); 131.1 (C-1′); 132.1 (C-4′); 148.2 (C-2′′); 157.9 (C-9); 162.4 (C-5); 162.6 (C-7); 164.4 (C-2); 164.7 (C(1′′)-NH-CO); 182.5 (C-4). HRMS: M+H=418.12807 (delta=-1.1ppm; $C_{24}H_{20}O_6N$). HR-ESI-MS-MS (CID=35%; rel. int. %): 400(11); 295(100); 267(57); 255(38); 237(5); 136(9).

S.1.4. *N*-(3,5-Dimethoxyphenyl)-2-((5-hydroxy-4-oxo-2-phenyl-4*H*-chromen-7-yl)oxy)acetamide (**10**)

Chrysin (**1**) (100 mg, 0.39 mmol), 2-chloro-*N*-(3,5-dimethoxyphenyl)acetamide (**5**) (95 mg, 0.41 mmol) and anhydrous potassium carbonate (56 mg, 0.41 mmol) were dissolved in DMF (4 mL). The reaction mixture was stirred at room temperature for 35 h, then, it was poured into water (30 mL). The precipitate was filtered off to afford 169 mg (96 %) of compound **10** as a white solid. M.p.: 237-238 °C. TLC (DCM : MeOH = 40 : 1); $R_f$ = 0.39. IR (KBr) 3288, 1665, 1613, 1376, 1159, 825 cm$^{-1}$. $^1$H NMR (499.9 MHz; DMSO-$d_6$) δ (ppm) 3.72 (s; 6H; C(3′′)-$OCH_3$, C(5′′)-$OCH_3$); 4.86 (s; 2H; C(7)-$OCH_2$); 6.26 (t; *J* = 2.3 Hz; 1H; H-4′′); 6.50 (d; *J* = 2.3 Hz; 1H; H-6); 6.89 (d; *J* = 2.3 Hz; 1H; H-8); 6.91 (d; *J* = 2.3 Hz; 2H; H-2′′, H-6′′); 7.07 (s; 1H; H-3); 7.57–7.62 (m; 2H; H-3′, H-5′); 7.62–7.66 (m; 1H; H-4′); 8.09–8.12 (m; 2H; H-2′, H-6′); 10.10 (s; 1H; C(1′′)-NH); 12.79 (br s; 1H; C(5)-OH). $^{13}$C NMR (125.7 MHz; DMSO-$d_6$) δ (ppm) 55.0 (C(3′′)-$OCH_3$, C(5′′)-$OCH_3$); 67.1 (C(7)-$OCH_2$); 93.6 (C-8); 95.6 (C-4′′); 97.8 (C-2′′, C-6′′); 98.6 (C-6); 105.2 (C-10); 105.3 (C-3); 126.4 (C-2′, C-6′); 129.1 (C-3′, C-5′); 130.5 (C-1′); 132.1 (C-4′); 139.9 (C-1′′); 157.1 (C-9); 160.4 (C-3′′, C-5′′); 161.1 (C-5); 163.5 (C-2); 163.8 (C-7); 165.6 (C(1′′)-NH-CO); 182.0 (C-4). HRMS: M+H=448.13910 (delta=0.05 ppm; $C_{25}H_{22}O_7N$). HR-ESI-MS-MS (CID=55%; rel. int. %): 430(6); 412(3); 295(100); 267(5); 255(3).

S.1.5. 2-((5-Hydroxy-4-oxo-2-phenyl-4*H*-chromen-7-yl)oxy)-*N*-(2-(trifluoromethyl)phenyl)-acetamide (**11**)

Chrysin (**1**) (50 mg, 0.20 mmol), 2-chloro-*N*-(2-(trifluoromethyl)phenyl)acetamide (**6**) (49 mg, 0.21 mmol) and anhydrous potassium carbonate (28 mg, 0.20 mmol) were dissolved in DMF (2 mL). The reaction mixture was stirred at room temperature for 24 h, then, it was poured into water (30 mL). After filtering off the precipitate, the filtrate was extracted with DCM (3 x 30 mL). The

combined organic layer was dried over $MgSO_4$ and concentrated *in vacuo*. The filtered precipitation and the crude product obtained by extraction were purified separately with preparative TLC (DCM : MeOH = 40 : 1). Combining the pure products 84 mg (94 %) of compound **11** was isolated as a white solid. M.p.: 263 °C (decomp.). TLC (DCM : MeOH = 40 : 1); $R_f$ = 0.64. IR (KBr) 3425, 3076, 1707, 1663, 1170, 1117, 766, 758 $cm^{-1}$. $^1H$ NMR (399.8 MHz; DMSO-$d_6$) δ (ppm) 4.92 (s; 2H; C(7)-OC$\underline{H}_2$); 6.47 (br d; *J* = 1.4 Hz; 1H; H-6); 6.84 (br d; *J* = 1.4 Hz; 1H; H-8); 7.07 (s; 1H; H-3); 7.46–7.52 (m; 1H; H-4′′); 7.57–7.67 (m; 4H; H-3′, H-5′, H-6′′, H-4′); 7.69–7.75 (m; 1H; H-5′′); 7.75–7.80 (m; 1H; H-3′′); 8.08–8.13 (m; 2H; H-2′, H-6′); 9.9 (br; C(1′′)-N$\underline{H}$); 12.8 (br; C(5)-O$\underline{H}$). $^{13}C$ NMR (101.5 MHz; DMSO-$d_6$) δ (ppm) 67.0 (C(7)-O$\underline{C}H_2$); 93.3 (br; C-8); 98.7 (C-6); 105.4 (C-3, C-10); 123.5 (q; $^1J_{FC}$ = 273.6 Hz; C(2′′)-$\underline{C}F_3$); 124.5 (q; $^2J_{FC}$ = 29.3 Hz; C-2′′); 126.2–126.4 (m; C-3′′, C-2′, C-6′); 126.9 (C-4′′); 129.1 (C-3′, C-5′); 129.5 (C-6′′); 130.5 (C-1′); 132.1 (C-4′); 133.1 (C-5′′); 134.6 (C-1′′); 157.2 (C-9); 161.3 (C-5); 163.4 (C-7, C-2); 166.8 (C(1′′)-NH-$\underline{C}$O); 182.0 (C-4). $^{19}F$ NMR (376.2 MHz; DMSO-$d_6$) δ (ppm; ref.: $CFCl_3$) -59.3. HRMS: M+H=456.10417 (delta=-2.6ppm; $C_{24}H_{17}O_5NF_3$). HR-ESI-MS-MS (CID=35%; rel. int. %): 416(4); 396(100); 368(3); 358(5); 352(20); 295(3); 267(7); 255(15); 237(2).

S.1.6. 5-Hydroxy-2-phenyl-7-(phenylamino)-4*H*-chromen-4-one (**12**)

Chrysin (**1**) (100 mg, 0.39 mmol), 2-chloro-*N*-phenylacetamide (**2**) (71 mg, 0.42 mmol) and anhydrous potassium carbonate (112 mg, 0.79 mmol) were dissolved in DMF (4 mL). The reaction mixture was stirred at 85 °C for 49 h, then, it was poured into water (30 mL). After filtering off the precipitate, the filtrate was diluted with saturated NaCl solution (10 mL) and extracted with DCM (3 x 30 mL). The combined organic layer was washed with water (20 mL), dried over $MgSO_4$ and concentrated *in vacuo*. The filtered precipitation and the crude product obtained by extraction were purified separately with preparative TLC (DCM : MeOH = 60 : 1). Combining the pure products 37 mg (29 %) of compound **12** was isolated as a yellow solid. M.p.: 233-236 °C. TLC (DCM : MeOH = 60 : 1); $R_f$ = 0.70. IR (KBr) 3307, 3202, 1654, 1619, 1406, 1171, 744, 504 $cm^{-1}$. $^1H$ NMR (499.9 MHz; DMSO-$d_6$) δ (ppm) 6.32 (d; *J* = 1.9 Hz; 1H; H-6); 6.66 (d; *J* = 1.9 Hz; 1H; H-8); 6.94 (s; 1H; H-3); 7.09–7.14 (m; 1H; H-4′′); 7.25–7.29 (m; 2H; H-2′′, H-6′′); 7.38–7.43 (m; 2H; H-3′′, H-5′′); 7.54–7.62 (m; 3H; H-3′, H-5′, H-4′); 8.05–8.09 (m; 2H; H-2′, H-6′); 9.10 (s; 1H; C(7)-N$\underline{H}$); 12.80 (s; 1H; C(5)-O$\underline{H}$). $^{13}C$ NMR (125.7 MHz; DMSO-$d_6$) δ (ppm) 90.9 (C-8); 97.0 (C-6); 103.2 (C-10); 105.1 (C-3); 121.0 (C-2′′, C-6′′); 123.2 (C-4′′); 126.3 (C-2′, C-6′); 129.0 (C-3′, C-5′); 129.4 (C-3′′, C-5′′); 130.7 (C-1′); 131.8 (C-4′); 140.1 (C-1′′); 151.3 (C-7); 157.6 (C-9); 160.9 (C-5); 162.7 (C-2); 181.0 (C-4). EI-HRMS: M=329.10387 (delta=-2.4 ppm; $C_{21}H_{15}O_3N$).

S.1.7. 5-Hydroxy-2-phenyl-7-(*o*-tolylamino)-4*H*-chromen-4-one (**13**)

Chrysin (**1**) (50 mg, 0.20 mmol), 2-chloro-*N*-(*o*-tolyl)acetamide (**3**) (38 mg, 0.21 mmol) and anhydrous potassium carbonate (56 mg, 0.41 mmol) were dissolved in DMF (2 mL). The reaction mixture was stirred at 80 °C for 61 h, then, it was evaporated under reduced pressure. Water (30 mL) was added to the residue and it was extracted with DCM (30 mL). Then, saturated NaCl solution (20 mL) was added to the aqueous phase and it was extracted with DCM (2 x 20 mL). The combined organic layer was dried over $MgSO_4$ and concentrated *in vacuo*. The crude product was purified with preparative TLC (DCM : MeOH = 40 : 1) to give 31 mg (46 %) of compound **13** as a yellow solid. M.p.: 220-221 °C. TLC (DCM : MeOH = 40 : 1); $R_f$ = 0.69. IR (KBr) 3417, 3059, 1660, 1450, 1172, 1045, 766 $cm^{-1}$. $^1H$ NMR (499.9 MHz; DMSO-$d_6$) δ (ppm) 2.22 (s; 3H; C(2′′)-C$\underline{H}_3$); 6,10 (d; *J* = 2.0 Hz; 1H; H-6); 6.24 (d; *J* = 2.0 Hz; 1H; H-8); 6.89 (s; 1H; H-3); 7.16–7.21 (m; 1H; H-4′′); 7.25–7.30 (m; 2H; H-5′′, H-6′′); 7.33–7.36 (m; 1H; H-3′′); 7.52–7.56 (m; 2H; H-3′, H-5′); 7.57–7.61 (m; 1H; H-4′); 8.01–8.04 (m; 2H; H-2′, H-6′); 8.61 (s; 1H; C(7)-N$\underline{H}$); 12.81 (s; 1H; C(5)-O$\underline{H}$). $^{13}C$ NMR (125.7 MHz; DMSO-$d_6$) δ (ppm) 17.6 (C(2′′)-$\underline{C}H_3$); 90.1 (C-8); 96.0 (C-6); 102.5 (C-10); 105.0 (C-3); 125.5 (C-6′′); 125.6 (C-4′′); 126.2 (C-2′, C-6′); 126.8 (C-5′′); 129.0 (C-3′, C-5′); 130.7 (C-1′); 131.1 (C-3′′); 131.7 (C-4′); 133.4 (C-2′′); 137.9 (C-1′′); 153.2 (C-7); 157.6 (C-9); 161.0 (C-5); 162.5 (C-2); 180.9 (C-4). HRMS: M+H=344.12840 (delta=0.81ppm; $C_{22}H_{18}O_3N$).

HR-ESI-MS-MS (CID=65%; rel. int. %): 326(32); 311(3); 288(4); 270(2); 242(100); 225(13); 209(8); 172(7); 158(3); 129(5).

S.1.8. 5-Hydroxy-7-((2-methoxyphenyl)amino)-2-phenyl-4*H*-chromen-4-one (**14**)

Chrysin (**1**) (150 mg, 0.59 mmol), 2-chloro-*N*-(2-methoxyphenyl)acetamide (**4**) (126 mg, 0.63 mmol) and anhydrous potassium carbonate (168 mg, 1.2 mmol) were dissolved in DMF (10 mL). The reaction mixture was stirred at 105 °C for 31 h, then, it was evaporated under reduced pressure. Water (30 mL) was added to the residue and it was extracted with DCM (4x 40 mL). The combined organic layer was dried over $MgSO_4$ and concentrated *in vacuo*. The crude product was purified with preparative TLC (DCM : MeOH = 40 : 1) to give 71 mg (33 %) of compound **14** as a yellow solid. M.p.: 156-157 °C. TLC (DCM : MeOH = 40 : 1); $R_f$ = 0.90. IR (KBr) 3348, 2963, 1668, 1586, 1512, 1257, 1227, 1024, 736 $cm^{-1}$. $^1H$ NMR (499.9 MHz; DMSO-$d_6$) δ (ppm) 3.82 (s; 3H; C(2′′)-OC<u>H</u>$_3$); 6.23 (d; *J* = 2.0 Hz; 1H; H-6); 6.42 (d; *J* = 2.0 Hz; 1H; H-8); 6.89 (s; 1H; H-3); 7.00 (ddd; *J* = 7.8, 7.3, 1.3 Hz; 1H; H-5′′); 7.14 (dd; *J* = 8.3, 1.3 Hz; 1H; H-3′′); 7.20 (ddd; *J* = 8.3, 7.3, 1.5 Hz; 1H; H-4′′); 7.33 (dd; *J* = 7.8, 1.5 Hz; 1H; H-6′′); 7.53–7.57 (m; 2H; H-3′, H-5′); 7.57–7.61 (m; 1H; H-4′); 8.01–8.05 (m; 2H; H-2′, H-6′); 8.53 (s; 1H; C(7)-N<u>H</u>); 12.77 (s; 1H; C(5)-O<u>H</u>). $^{13}C$ NMR (125.7 MHz; DMSO-$d_6$) δ (ppm) 55.4 (C(2′′)-O<u>C</u>H$_3$); 90.6 (C-8); 96.6 (C-6); 102.5 (C-10); 105.0 (C-3); 112.2 (C-3′′); 120.6 (C-5′′); 124.3 (C-6′′); 125.6 (C-4′′); 126.2 (C-2′, C-6′); 128.1 (C-1′′); 129.0 (C-3′, C-5′); 130.8 (C-1′); 131.7 (C-4′); 152.6, 152.7 (C-9, C-2′′); 157.5 (C-7); 160.8 (C-5); 162.5 (C-2); 180.9 (C-4). HRMS: M+H=360.12216 (delta=-2.4 ppm; $C_{22}H_{18}O_4N$). HR-ESI-MS-MS (CID=55%; rel. int. %): 345(100); 344(25); 330(2); 328(48); 327(2); 240(2).

S.1.9. 7-((3,5-Dimethoxyphenyl)amino)-5-hydroxy-2-phenyl-4*H*-chromen-4-one (**15**)

Chrysin (**1**) (50 mg, 0.20 mmol), 2-chloro-*N*-(3,5-dimethoxyphenyl)acetamide (**5**) (48 mg, 0.21 mmol) and anhydrous potassium carbonate (56 mg, 0.41 mmol) were dissolved in DMF (4 mL). The reaction mixture was stirred at 90 °C for 58 h, then, it was evaporated under reduced pressure. Water (20 mL) was added to the residue and it was extracted with DCM (4 x 20 mL). The combined organic layer was washed with water (20 mL), dried over $MgSO_4$ and concentrated *in vacuo*. The crude product was purified with preparative TLC (DCM : MeOH = 40 : 1) to give 22 mg (29 %) of compound **15** as a yellow solid. M.p.: 202-203 °C. TLC (DCM : MeOH = 40 : 1); $R_f$ = 0.68. IR (KBr) 3276, 2998, 2832, 1660, 1615, 1450, 1208, 1154, 673 $cm^{-1}$. $^1H$ NMR (799.7 MHz; DMSO-$d_6$) δ (ppm) 3.76 (s; 6H; C(3′′)-OC<u>H</u>$_3$, C(5′′)-OC<u>H</u>$_3$); 6.27 (t; *J* = 2.2 Hz; 1H; H-4′′); 6.34 (d; *J* = 1.9 Hz; 1H; H-6); 6.39 (d; *J* = 2.2 Hz; 2H; H-2′′, H-6′′); 6.70 (d; *J* = 1.9 Hz; 1H; H-8); 6.94 (s; 1H; H-3); 7.55–7.58 (m; 2H; H-3′, H-5′); 7.59–7.62 (m; 1H; H-4′); 8.06–8.09 (m; 2H; H-2′, H-6′); 9.07 (s; 1H; C(7)-N<u>H</u>); 12.80 (s; 1H; C(5)-O<u>H</u>). $^{13}C$ NMR (201.1 MHz; DMSO-$d_6$) δ (ppm) 55.1 (C(3′′)-O<u>C</u>H$_3$, C(5′′)-O<u>C</u>H$_3$); 91.6 (C-8); 95.0 (C-4′′); 97.5 (C-6); 98.9 (C-2′′, C-6′′); 103.3 (C-10); 105.1 (C-3); 126.3 (C-2′, C-6′); 129.0 (C-3′, C-5′); 130.7 (C-1′); 131.8 (C-4′); 141.9 (C-1′′); 150.9 (C-7); 157.5 (C-9); 160.8 (C-5); 161.0 (C-3′′, C-5′′); 162.8 (C-2); 181.0 (C-4). HRMS: M+H=390.13374 (delta=0.4ppm; $C_{23}H_{20}O_5N$). HR-ESI-MS-MS (CID=65%; rel. int. %): 374(100); 357(7); 343(2).

S.2.10. *N*-(5-Hydroxy-4-oxo-2-phenyl-4*H*-chromen-7-yl)-2-(2-(trifluoromethyl)phenoxy)-acetamide (**20**)

Chrysin (**1**) (100 mg, 0.39 mmol), 2-chloro-*N*-(2-(trifluoromethyl)phenyl)acetamide (**6**) (98 mg, 0.41 mmol) and anhydrous potassium carbonate (112 mg, 0.81 mmol) were dissolved in DMF (4 mL). The reaction mixture was stirred at 75 °C for 9 h, then, it was poured into water (30 mL). After filtering off the precipitate, the filtrate was diluted with saturated NaCl solution (30 mL) and extracted with DCM (4 x 30 mL). The combined organic layer was washed with water (30 mL), dried over $MgSO_4$ and concentrated *in vacuo*. The filtered precipitation and the crude product obtained by extraction were purified separately with preparative TLC (DCM : MeOH = 60 : 1). Combining the pure products 50 mg (28 %) of compound **20** was isolated as a white solid. M.p.: 210-212 °C. TLC

(DCM : MeOH = 60 : 1); $R_f$ = 0.71. IR (KBr) 3398, 3057, 1662, 1524, 1323, 1132, 759, 674 $cm^{-1}$. $^1$H NMR (499.9 MHz; DMSO-$d_6$) $\delta$ (ppm) 4.99 (s; 2H; C(1'')-OC<u>H</u>2); 6.90 (d; $J$ = 1.7 Hz; 1H; H-6); 7.08 (s; 1H; H-3); 7.14 (t; $J$ = 7.5 Hz; 1H; H-4''); 7.21 (d; $J$ = 8.5 Hz; 1H; H-6''); 7.56–7.67 (m; 6H; H-3', H-5', H-4', H-5'', H-3'', H-8); 8.09–8.13 (m; 2H; H-2', H-6'); 10.69 (s; 1H; C(7)-N<u>H</u>); 12.71 (s; 1H; C(5)-O<u>H</u>). $^{13}$C NMR (125.7 MHz; DMSO-$d_6$) $\delta$ (ppm) 67.0 (C(1'')-O<u>C</u>H2); 97.3 (C-8); 101.1 (C-6); 105.5 (C-3); 106.5 (C-10); 113.6 (C-6''); 117.0 (q; $^2J_{FC}$ = 30.2 Hz; C-2''); 120.8 (C-4''); 123.6 (q; $^1J_{FC}$ = 272.1 Hz; C(2'')-<u>C</u>F3); 126.5 (C-2', C-6'); 126.8 (q; $^3J_{FC}$ = 4.9 Hz; C-3''); 129.1 (C-3', C-5'); 130.5 (C-1'); 132.2 (C-4'); 134.0 (C-5''); 144.8 (C-7); 155.7 (C-1''); 156.6 (C-9); 160.2 (C-5); 163.8 (C-2); 166.7 (C(7)-NH-<u>C</u>O); 182.0 (C-4). HRMS: M+H=456.10404 (delta=-2.8ppm; $C_{24}H_{17}O_5NF_3$). HR-ESI-MS-MS (CID=35%; rel. int. %): 436(82); 416(59); 396(22); 388(100); 358(69); 314(25); 294(59); 266(76).

**Table S1.** Energy values obtained for the computation of the 1→2 transformation and the related dimerization. The E, ZPE, U, H and G values were computed using the B3LYP/6-31 (d,p) method and are given in Hartree.

| ID | E | ZPE | U | H | G |
|---|---|---|---|---|---|
| **1-H+** | -686.329374 | -686.178589 | -686.16757 | -686.166626 | -686.215244 |
| | | | | | |
| **2** | -899.880062 | -899.733174 | -899.723225 | -899.722281 | -899.771061 |
| **TS2** | -1586.200932 | -1585.902769 | -1585.880373 | -1585.879429 | -1585.958358 |
| **7** | -1586.256283 | -1585.954988 | -1585.932413 | -1585.931469 | -1586.011925 |
| **TS17a** | -1125.349621 | -1125.064369 | -1125.045192 | -1125.044248 | -1125.112469 |
| **18a** | -1125.35433 | -1125.068375 | -1125.048709 | -1125.047765 | -1125.118161 |
| **TS18a** | -1201.789305 | -1201.479757 | -1201.457341 | -1201.456397 | -1201.532498 |
| **19a** | -1201.797901 | -1201.48845 | -1201.465642 | -1201.464698 | -1201.54203 |
| **TS19a** | -2590.113717 | -2589.788308 | -2589.759818 | -2589.758874 | -2589.851482 |
| **12** | -2590.165232 | -2589.837609 | -2589.806734 | -2589.80579 | -2589.907816 |
| | | | | | |
| **3** | -939.196944 | -939.021998 | -939.010525 | -939.009581 | -939.061674 |
| **TS4** | -1625.52009 | -1625.193985 | -1625.170077 | -1625.169133 | -1625.251148 |
| **8** | -1625.575712 | -1625.24654 | -1625.222444 | -1625.2215 | -1625.304732 |
| **TS17b** | -1164.667656 | -1164.354786 | -1164.333898 | -1164.332954 | -1164.404827 |
| **18b** | -1164.674747 | -1164.361023 | -1164.339799 | -1164.338855 | -1164.411551 |
| **TS18b** | -1241.110539 | -1240.772878 | -1240.749141 | -1240.748197 | -1240.825996 |
| **19b** | -1241.118663 | -1240.781652 | -1240.757325 | -1240.756381 | -1240.836324 |
| **TS19b** | -2629.43213 | -2629.079031 | -2629.048855 | -2629.047911 | -2629.143327 |
| **13** | -2629.485447 | -2629.129779 | -2629.097511 | -2629.096567 | -2629.200609 |
| | | | | | |
| **4** | -1014.404738 | -1014.224854 | -1014.212336 | -1014.211391 | -1014.266518 |
| **TS4** | -1700.727676 | -1700.396788 | -1700.371816 | -1700.370872 | -1700.45534 |
| **9** | -1700.782981 | -1700.449208 | -1700.423959 | -1700.423015 | -1700.509838 |
| **TS17c** | -1239.871867 | -1239.554095 | -1239.532245 | -1239.531301 | -1239.60573 |
| **18c** | -1239.879217 | -1239.560714 | -1239.538414 | -1239.53747 | -1239.613382 |
| **TS18c** | -1316.307867 | -1315.967261 | -1315.941811 | -1315.940867 | -1316.025548 |
| **19c** | -1316.322218 | -1315.9798 | -1315.954459 | -1315.953515 | -1316.03614 |
| **TS19c** | -2704.685705 | -2704.324267 | -2704.291312 | -2704.290368 | -2704.393983 |
| **14** | -2704.636492 | -2704.276809 | -2704.245624 | -2704.24468 | -2704.342729 |
| | | | | | |
| **5** | -1128.933309 | -1128.721617 | -1128.706345 | -1128.705401 | -1128.76654 |
| **TS5** | -1815.254057 | -1814.891005 | -1814.863317 | -1814.862373 | -1814.953056 |
| **10** | -1815.309407 | -1814.943366 | -1814.915457 | -1814.914513 | -1815.007363 |
| **TS17d** | -1354.402003 | -1354.051998 | -1354.027451 | -1354.026507 | -1354.107248 |
| **18d** | -1354.406686 | -1354.055926 | -1354.030949 | -1354.030004 | -1354.112475 |
| **TS18d** | -1430.842 | -1430.467912 | -1430.44005 | -1430.439106 | -1430.527921 |
| **19d** | -1430.84801 | -1430.474038 | -1430.445694 | -1430.444749 | -1430.534964 |
| **TS19d** | -2819.166854 | -2818.776692 | -2818.742856 | -2818.741912 | -2818.846318 |
| **15** | -2819.219063 | -2818.826551 | -2818.790391 | -2818.789447 | -2818.902673 |
| | | | | | |

| | | | | | |
|---|---|---|---|---|---|
| **6** | -1236.912454 | -1236.760834 | -1236.747375 | -1236.746431 | -1236.803828 |
| **TS6** | -1923.233964 | -1922.930973 | -1922.905091 | -1922.904147 | -1922.991113 |
| **11** | -1923.289504 | -1922.983649 | -1922.957479 | -1922.956535 | -1923.046153 |
| **TS17e** | -1462.383761 | -1462.093956 | -1462.071161 | -1462.070217 | -1462.1469 |
| **18e** | -1462.386967 | -1462.096214 | -1462.073139 | -1462.072195 | -1462.14961 |
| **TS18e** | -1538.823451 | -1538.508691 | -1538.483047 | -1538.482103 | -1538.564499 |
| **19e** | -1538.830415 | -1538.516366 | -1538.489914 | -1538.48897 | -1538.574152 |
| **TS19e** | -2927.14196 | -2926.812256 | -2926.780573 | -2926.779628 | -2926.880775 |
| **16** | -2927.200824 | -2926.869055 | -2926.834593 | -2926.833648 | -2926.943602 |
| | | | | | |
| **23-24 TS** | -1462.375226 | -1462.086156 | -1462.063323 | -1462.062379 | -1462.13876 |
| **24** | -1462.381425 | -1462.091145 | -1462.068118 | -1462.067173 | -1462.144064 |
| **24-20-H⁺ TS** | -1462.380416 | -1462.090589 | -1462.068037 | -1462.067093 | -1462.142715 |
| **20-H⁺** | -1462.413081 | -1462.121694 | -1462.098254 | -1462.09731 | -1462.17723 |

# Coordinates of the Computed Structures

**1-H⁺**

| Center Number | Atomic Number | Atomic Type | X | Y | Z |
|---|---|---|---|---|---|
| 1 | 6 | 0 | -2.425475 | -1.144505 | -0.000236 |
| 2 | 6 | 0 | -1.080869 | -1.687854 | 0.000051 |
| 3 | 6 | 0 | 0.009834 | -0.853513 | 0.000016 |
| 4 | 6 | 0 | -0.088820 | 0.563282 | -0.000124 |
| 5 | 6 | 0 | -1.412986 | 1.117833 | 0.000029 |
| 6 | 6 | 0 | -2.523516 | 0.303661 | -0.000065 |
| 7 | 1 | 0 | -0.946351 | -2.764562 | 0.000209 |
| 8 | 6 | 0 | 1.080570 | 1.384647 | -0.000409 |
| 9 | 1 | 0 | -3.514193 | 0.749691 | 0.000021 |
| 10 | 6 | 0 | 2.348622 | 0.670031 | -0.000205 |
| 11 | 6 | 0 | 2.389380 | -0.682547 | -0.000376 |
| 12 | 1 | 0 | 3.271015 | 1.238628 | 0.000075 |
| 13 | 6 | 0 | 3.623649 | -1.525648 | 0.000313 |
| 14 | 1 | 0 | 4.517305 | -0.900239 | -0.000357 |
| 15 | 1 | 0 | 3.644034 | -2.173625 | 0.883237 |
| 16 | 1 | 0 | 3.643872 | -2.175385 | -0.881324 |
| 17 | 8 | 0 | 1.034492 | 2.654911 | 0.000095 |
| 18 | 8 | 0 | -1.541859 | 2.470139 | 0.000224 |
| 19 | 8 | 0 | -3.448771 | -1.887131 | 0.000096 |
| 20 | 8 | 0 | 1.264249 | -1.446320 | -0.000007 |
| 21 | 1 | 0 | -0.602910 | 2.820365 | 0.000901 |

**2**

| Center Number | Atomic Number | Atomic Type | X | Y | Z |
|---|---|---|---|---|---|
| 1 | 6 | 0 | -2.696133 | -0.004752 | 0.790177 |
| 2 | 1 | 0 | -3.311200 | 0.783515 | 1.218148 |
| 3 | 1 | 0 | -2.667172 | -0.868648 | 1.453702 |
| 4 | 6 | 0 | -1.316435 | 0.567935 | 0.466841 |
| 5 | 8 | 0 | -1.176091 | 1.760711 | 0.215414 |
| 6 | 7 | 0 | -0.315732 | -0.360419 | 0.482098 |
| 7 | 17 | 0 | -3.523904 | -0.545324 | -0.741398 |
| 8 | 1 | 0 | -0.587741 | -1.307072 | 0.711492 |
| 9 | 6 | 0 | 1.058391 | -0.192377 | 0.196187 |
| 10 | 6 | 0 | 1.868390 | -1.337719 | 0.264594 |
| 11 | 6 | 0 | 1.630891 | 1.043323 | -0.143017 |
| 12 | 6 | 0 | 3.231962 | -1.250170 | -0.001793 |
| 13 | 6 | 0 | 2.999828 | 1.112984 | -0.407473 |
| 14 | 1 | 0 | 1.008185 | 1.924294 | -0.195205 |
| 15 | 6 | 0 | 3.807625 | -0.022872 | -0.339993 |
| 16 | 1 | 0 | 3.843774 | -2.145287 | 0.055481 |
| 17 | 1 | 0 | 3.434736 | 2.073143 | -0.669254 |
| 18 | 1 | 0 | 4.870742 | 0.045596 | -0.547716 |
| 19 | 1 | 0 | 1.426784 | -2.295907 | 0.525978 |

**TS2**

| Center Number | Atomic Number | Atomic Type | X | Y | Z |
|---|---|---|---|---|---|
| 1 | 6 | 0 | -0.835951 | 0.528634 | 1.129791 |
| 2 | 6 | 0 | -1.312136 | -0.297221 | 0.066892 |
| 3 | 6 | 0 | -2.633759 | -0.201447 | -0.322799 |
| 4 | 6 | 0 | -3.559070 | 0.681459 | 0.275660 |
| 5 | 6 | 0 | -3.076484 | 1.511405 | 1.334913 |
| 6 | 6 | 0 | -1.759414 | 1.430515 | 1.748294 |
| 7 | 1 | 0 | -0.634386 | -0.959980 | -0.456978 |
| 8 | 6 | 0 | -4.923822 | 0.739753 | -0.178188 |
| 9 | 1 | 0 | -1.409391 | 2.060420 | 2.559373 |
| 10 | 6 | 0 | -5.254227 | -0.155878 | -1.270949 |
| 11 | 6 | 0 | -4.321766 | -0.979183 | -1.808146 |
| 12 | 1 | 0 | -6.265355 | -0.159181 | -1.659551 |
| 13 | 6 | 0 | -4.540049 | -1.939459 | -2.931322 |
| 14 | 1 | 0 | -5.573009 | -1.896863 | -3.278373 |
| 15 | 1 | 0 | -3.872742 | -1.705699 | -3.767616 |
| 16 | 1 | 0 | -4.314139 | -2.961188 | -2.608225 |
| 17 | 8 | 0 | -5.778611 | 1.517737 | 0.335690 |
| 18 | 8 | 0 | -3.932097 | 2.371048 | 1.933766 |
| 19 | 8 | 0 | 0.395115 | 0.500841 | 1.548906 |
| 20 | 8 | 0 | -3.039547 | -1.017698 | -1.360271 |
| 21 | 6 | 0 | 1.429104 | -1.318154 | 1.556059 |
| 22 | 1 | 0 | 0.497357 | -1.833244 | 1.401156 |
| 23 | 1 | 0 | 1.683734 | -0.979575 | 2.545171 |
| 24 | 1 | 0 | -4.808008 | 2.237048 | 1.468726 |
| 25 | 6 | 0 | 2.168196 | -0.854112 | 0.336246 |
| 26 | 8 | 0 | 1.806750 | -1.175264 | -0.794588 |
| 27 | 7 | 0 | 3.256232 | -0.070699 | 0.613935 |
| 28 | 17 | 0 | 2.546626 | -3.242111 | 1.842628 |
| 29 | 1 | 0 | 3.423749 | 0.122956 | 1.591558 |
| 30 | 6 | 0 | 4.164134 | 0.536393 | -0.275882 |
| 31 | 6 | 0 | 5.183978 | 1.322346 | 0.288300 |
| 32 | 6 | 0 | 4.099024 | 0.391168 | -1.671555 |
| 33 | 6 | 0 | 6.121379 | 1.951710 | -0.525679 |
| 34 | 6 | 0 | 5.047233 | 1.029202 | -2.473032 |
| 35 | 1 | 0 | 3.316201 | -0.212030 | -2.107235 |
| 36 | 6 | 0 | 6.060162 | 1.809934 | -1.914439 |
| 37 | 1 | 0 | 6.901376 | 2.554692 | -0.070397 |
| 38 | 1 | 0 | 4.986996 | 0.909823 | -3.551069 |
| 39 | 1 | 0 | 6.790909 | 2.300739 | -2.549544 |
| 40 | 1 | 0 | 5.238355 | 1.437162 | 1.367776 |

**7**

| Center Number | Atomic Number | Atomic Type | X | Y | Z |
|---|---|---|---|---|---|
| 1 | 6 | 0 | -0.577162 | 1.391273 | -0.247225 |
| 2 | 6 | 0 | -1.096109 | 0.571233 | 0.761247 |
| 3 | 6 | 0 | -2.385481 | 0.075082 | 0.583270 |
| 4 | 6 | 0 | -3.165081 | 0.361376 | -0.546537 |
| 5 | 6 | 0 | -2.602239 | 1.199023 | -1.552990 |
| 6 | 6 | 0 | -1.318279 | 1.706679 | -1.397846 |
| 7 | 1 | 0 | -0.544378 | 0.299171 | 1.650264 |
| 8 | 6 | 0 | -4.502547 | -0.190641 | -0.674997 |
| 9 | 1 | 0 | -0.886646 | 2.347417 | -2.157397 |
| 10 | 6 | 0 | -4.931459 | -1.027579 | 0.423815 |
| 11 | 6 | 0 | -4.121727 | -1.262068 | 1.486364 |
| 12 | 1 | 0 | -5.918606 | -1.471553 | 0.390912 |
| 13 | 6 | 0 | -4.449316 | -2.102324 | 2.674904 |
| 14 | 1 | 0 | -5.451137 | -2.522448 | 2.583610 |
| 15 | 1 | 0 | -3.726106 | -2.918527 | 2.772844 |
| 16 | 1 | 0 | -4.394153 | -1.502615 | 3.589327 |
| 17 | 8 | 0 | -5.225924 | 0.048750 | -1.675836 |
| 18 | 8 | 0 | -3.318239 | 1.496798 | -2.649620 |
| 19 | 8 | 0 | 0.673469 | 1.939820 | -0.208915 |
| 20 | 8 | 0 | -2.873759 | -0.729488 | 1.579428 |
| 21 | 6 | 0 | 1.472130 | 1.782404 | 0.968092 |

| | | | | | |
|---|---|---|---|---|---|
| 22 | 1 | 0 | 0.887780 | 1.940538 | 1.877822 |
| 23 | 1 | 0 | 2.243871 | 2.561530 | 0.902070 |
| 24 | 1 | 0 | -4.193831 | 1.025933 | -2.536497 |
| 25 | 6 | 0 | 2.175358 | 0.426437 | 1.072331 |
| 26 | 8 | 0 | 2.554720 | 0.017807 | 2.167658 |
| 27 | 7 | 0 | 2.371082 | -0.194712 | -0.125103 |
| 28 | 17 | 0 | 4.233152 | 4.206712 | 0.546924 |
| 29 | 1 | 0 | 1.996868 | 0.299020 | -0.926537 |
| 30 | 6 | 0 | 3.011658 | -1.421558 | -0.401890 |
| 31 | 6 | 0 | 3.042623 | -1.840959 | -1.741820 |
| 32 | 6 | 0 | 3.606103 | -2.219934 | 0.587902 |
| 33 | 6 | 0 | 3.657800 | -3.040938 | -2.088551 |
| 34 | 6 | 0 | 4.217375 | -3.420819 | 0.223312 |
| 35 | 1 | 0 | 3.584209 | -1.895202 | 1.618149 |
| 36 | 6 | 0 | 4.249348 | -3.840524 | -1.107358 |
| 37 | 1 | 0 | 3.673327 | -3.349520 | -3.129534 |
| 38 | 1 | 0 | 4.674868 | -4.032135 | 0.995861 |
| 39 | 1 | 0 | 4.728732 | -4.776158 | -1.377544 |
| 40 | 1 | 0 | 2.583918 | -1.223054 | -2.509182 |

**TS17a**

| Center Number | Atomic Number | Atomic Type | Coordinates (Angstroms) X | Y | Z |
|---|---|---|---|---|---|
| 1 | 6 | 0 | 0.689428 | -1.511595 | -0.406320 |
| 2 | 6 | 0 | -0.399299 | -1.079735 | -1.250975 |
| 3 | 6 | 0 | -1.549970 | -0.585232 | -0.691680 |
| 4 | 6 | 0 | -1.783965 | -0.528844 | 0.706948 |
| 5 | 6 | 0 | -0.742100 | -1.048720 | 1.550134 |
| 6 | 6 | 0 | 0.416086 | -1.559651 | 1.021296 |
| 7 | 1 | 0 | -0.281621 | -1.121235 | -2.327207 |
| 8 | 6 | 0 | -3.009287 | -0.024957 | 1.231796 |
| 9 | 1 | 0 | 1.168703 | -1.963123 | 1.691273 |
| 10 | 6 | 0 | -3.973496 | 0.431867 | 0.239415 |
| 11 | 6 | 0 | -3.706040 | 0.352854 | -1.083799 |
| 12 | 1 | 0 | -4.922590 | 0.832295 | 0.575514 |
| 13 | 6 | 0 | -4.612527 | 0.776584 | -2.193960 |
| 14 | 1 | 0 | -5.547564 | 1.173408 | -1.796412 |
| 15 | 1 | 0 | -4.132660 | 1.546158 | -2.808225 |
| 16 | 1 | 0 | -4.837206 | -0.071177 | -2.850325 |
| 17 | 8 | 0 | -3.264090 | 0.023413 | 2.477290 |
| 18 | 8 | 0 | -0.949874 | -1.053452 | 2.892243 |
| 19 | 8 | 0 | 1.380777 | -2.622519 | -0.959747 |
| 20 | 8 | 0 | -2.530936 | -0.142694 | -1.562848 |
| 21 | 6 | 0 | 2.760247 | -2.630992 | -0.627757 |
| 22 | 1 | 0 | 3.302449 | -3.155298 | -1.420377 |
| 23 | 1 | 0 | 2.958593 | -3.153381 | 0.320945 |
| 24 | 1 | 0 | -1.860700 | -0.652082 | 3.013700 |
| 25 | 6 | 0 | 3.228692 | -1.181457 | -0.503022 |
| 26 | 8 | 0 | 4.422748 | -0.886642 | -0.329806 |
| 27 | 7 | 0 | 2.150072 | -0.396473 | -0.611246 |
| 28 | 6 | 0 | 2.120526 | 0.965732 | -0.292430 |
| 29 | 6 | 0 | 1.346272 | 1.836074 | -1.081571 |
| 30 | 6 | 0 | 2.793697 | 1.492407 | 0.827778 |
| 31 | 6 | 0 | 1.246084 | 3.189658 | -0.758797 |
| 32 | 6 | 0 | 2.699246 | 2.848452 | 1.136418 |
| 33 | 1 | 0 | 3.398486 | 0.831483 | 1.438085 |
| 34 | 6 | 0 | 1.923278 | 3.704863 | 0.349249 |
| 35 | 1 | 0 | 0.642435 | 3.845403 | -1.380516 |
| 36 | 1 | 0 | 3.229885 | 3.238248 | 2.001131 |
| 37 | 1 | 0 | 1.850271 | 4.760149 | 0.595354 |
| 38 | 1 | 0 | 0.830777 | 1.435787 | -1.947610 |

**18a**

| Center Number | Atomic Number | Atomic Type | Coordinates (Angstroms) X | Y | Z |
|---|---|---|---|---|---|
| 1 | 6 | 0 | 0.674195 | -1.287932 | -0.217510 |
| 2 | 6 | 0 | -0.378711 | -0.793864 | -1.130505 |
| 3 | 6 | 0 | -1.587674 | -0.374902 | -0.659614 |
| 4 | 6 | 0 | -1.940251 | -0.337629 | 0.721720 |
| 5 | 6 | 0 | -0.927672 | -0.792398 | 1.643342 |
| 6 | 6 | 0 | 0.294293 | -1.224892 | 1.215510 |
| 7 | 1 | 0 | -0.182805 | -0.816813 | -2.196848 |
| 8 | 6 | 0 | -3.211936 | 0.112443 | 1.150192 |
| 9 | 1 | 0 | 1.027501 | -1.558003 | 1.944895 |
| 10 | 6 | 0 | -4.122854 | 0.529448 | 0.086357 |
| 11 | 6 | 0 | -3.751788 | 0.476779 | -1.211328 |
| 12 | 1 | 0 | -5.111898 | 0.884250 | 0.352001 |
| 13 | 6 | 0 | -4.588634 | 0.871112 | -2.386042 |
| 14 | 1 | 0 | -5.571143 | 1.215733 | -2.060116 |
| 15 | 1 | 0 | -4.102672 | 1.672463 | -2.953608 |
| 16 | 1 | 0 | -4.718990 | 0.023340 | -3.067700 |
| 17 | 8 | 0 | -3.567827 | 0.159963 | 2.376920 |
| 18 | 8 | 0 | -1.234440 | -0.774013 | 2.970773 |
| 19 | 8 | 0 | 1.023654 | -2.682249 | -0.627169 |
| 20 | 8 | 0 | -2.521004 | 0.039124 | -1.603620 |
| 21 | 6 | 0 | 2.420328 | -2.880059 | -0.670212 |
| 22 | 1 | 0 | 2.710182 | -3.405790 | -1.588908 |
| 23 | 1 | 0 | 2.794654 | -3.467801 | 0.183523 |
| 24 | 1 | 0 | -2.172734 | -0.417523 | 3.010168 |
| 25 | 6 | 0 | 3.052052 | -1.500096 | -0.630911 |

| | | | | | |
|---|---|---|---|---|---|
| 26 | 8 | 0 | 4.254195 | -1.257981 | -0.737462 |
| 27 | 7 | 0 | 2.036337 | -0.618285 | -0.452357 |
| 28 | 6 | 0 | 2.255556 | 0.764510 | -0.195649 |
| 29 | 6 | 0 | 1.656590 | 1.739708 | -1.002481 |
| 30 | 6 | 0 | 3.077165 | 1.157952 | 0.869524 |
| 31 | 6 | 0 | 1.868805 | 3.093692 | -0.736114 |
| 32 | 6 | 0 | 3.298310 | 2.511778 | 1.121093 |
| 33 | 1 | 0 | 3.540240 | 0.400037 | 1.491693 |
| 34 | 6 | 0 | 2.691222 | 3.484360 | 0.322446 |
| 35 | 1 | 0 | 1.396966 | 3.843275 | -1.364678 |
| 36 | 1 | 0 | 3.938910 | 2.806499 | 1.947139 |
| 37 | 1 | 0 | 2.859669 | 4.538250 | 0.522941 |
| 38 | 1 | 0 | 1.027166 | 1.431268 | -1.828445 |

**TS18a**

| Center Number | Atomic Number | Atomic Type | Coordinates (Angstroms) X | Y | Z |
|---|---|---|---|---|---|
| 1 | 6 | 0 | -0.519108 | 0.761887 | 0.326939 |
| 2 | 6 | 0 | 0.285497 | 0.287717 | -0.760643 |
| 3 | 6 | 0 | 1.611460 | -0.000270 | -0.541795 |
| 4 | 6 | 0 | 2.218931 | 0.038675 | 0.737934 |
| 5 | 6 | 0 | 1.377089 | 0.394093 | 1.841560 |
| 6 | 6 | 0 | 0.044918 | 0.687587 | 1.649816 |
| 7 | 1 | 0 | -0.125005 | 0.253454 | -1.761934 |
| 8 | 6 | 0 | 3.598372 | -0.302057 | 0.910836 |
| 9 | 1 | 0 | -0.577468 | 0.931849 | 2.504351 |
| 10 | 6 | 0 | 4.308587 | -0.663611 | -0.305767 |
| 11 | 6 | 0 | 3.679103 | -0.685740 | -1.503766 |
| 12 | 1 | 0 | 5.358039 | -0.925162 | -0.242920 |
| 13 | 6 | 0 | 4.296386 | -1.045568 | -2.815959 |
| 14 | 1 | 0 | 5.350183 | -1.296605 | -2.689221 |
| 15 | 1 | 0 | 3.777679 | -1.901669 | -3.260648 |
| 16 | 1 | 0 | 4.211602 | -0.210822 | -3.519945 |
| 17 | 8 | 0 | 4.171337 | -0.296613 | 2.042994 |
| 18 | 8 | 0 | 1.911758 | 0.405193 | 3.086936 |
| 19 | 8 | 0 | -0.736972 | 2.607284 | 0.010528 |
| 20 | 8 | 0 | 2.363133 | -0.368984 | -1.642654 |
| 21 | 6 | 0 | -2.059586 | 2.879927 | 0.309893 |
| 22 | 1 | 0 | -2.549998 | 3.519420 | -0.445440 |
| 23 | 1 | 0 | -2.212023 | 3.379219 | 1.286624 |
| 24 | 1 | 0 | 2.870256 | 0.142270 | 2.960141 |
| 25 | 6 | 0 | -2.836113 | 1.568591 | 0.337835 |
| 26 | 8 | 0 | -4.057425 | 1.480837 | 0.435839 |
| 27 | 7 | 0 | -1.965860 | 0.509490 | 0.215577 |
| 28 | 6 | 0 | -2.433174 | -0.834737 | 0.086663 |
| 29 | 6 | 0 | -3.497321 | -1.121180 | -0.782436 |
| 30 | 6 | 0 | -1.830118 | -1.880385 | 0.799355 |
| 31 | 6 | 0 | -3.954749 | -2.430684 | -0.921509 |
| 32 | 6 | 0 | -2.287750 | -3.189625 | 0.643879 |
| 33 | 1 | 0 | -1.005975 | -1.664256 | 1.468658 |
| 34 | 6 | 0 | -3.353145 | -3.472935 | -0.211986 |
| 35 | 1 | 0 | -4.779648 | -2.636552 | -1.597551 |
| 36 | 1 | 0 | -1.808521 | -3.989155 | 1.201537 |
| 37 | 1 | 0 | -3.708957 | -4.492265 | -0.327347 |
| 38 | 1 | 0 | -3.963877 | -0.318686 | -1.339707 |
| 39 | 8 | 0 | -0.756543 | 2.982280 | -2.674915 |
| 40 | 1 | 0 | -0.661636 | 2.829690 | -1.698613 |
| 41 | 1 | 0 | -1.471605 | 2.382741 | -2.921926 |

**19a**

| Center Number | Atomic Number | Atomic Type | Coordinates (Angstroms) X | Y | Z |
|---|---|---|---|---|---|
| 1 | 6 | 0 | 0.331055 | 0.580321 | -0.309869 |
| 2 | 6 | 0 | -0.322703 | -0.507821 | 0.288556 |
| 3 | 6 | 0 | -1.706922 | -0.565761 | 0.222150 |
| 4 | 6 | 0 | -2.477725 | 0.415368 | -0.425226 |
| 5 | 6 | 0 | -1.791445 | 1.499961 | -1.038748 |
| 6 | 6 | 0 | -0.402762 | 1.562228 | -0.997378 |
| 7 | 1 | 0 | 0.228631 | -1.281161 | 0.807249 |
| 8 | 6 | 0 | -3.924279 | 0.303649 | -0.468559 |
| 9 | 1 | 0 | 0.102696 | 2.375810 | -1.502300 |
| 10 | 6 | 0 | -4.472800 | -0.854093 | 0.203433 |
| 11 | 6 | 0 | -3.668289 | -1.759370 | 0.815120 |
| 12 | 1 | 0 | -5.546557 | -0.994530 | 0.214754 |
| 13 | 6 | 0 | -4.117553 | -2.985101 | 1.538427 |
| 14 | 1 | 0 | -5.204468 | -3.067309 | 1.516572 |
| 15 | 1 | 0 | -3.681414 | -3.878017 | 1.078612 |
| 16 | 1 | 0 | -3.781571 | -2.954545 | 2.580096 |
| 17 | 8 | 0 | -4.641099 | 1.159329 | -1.050376 |
| 18 | 8 | 0 | -2.489267 | 2.451488 | -1.687038 |
| 19 | 8 | 0 | 1.002404 | 2.472553 | 1.763443 |
| 20 | 8 | 0 | -2.315541 | -1.637277 | 0.830664 |
| 21 | 6 | 0 | 1.824457 | 2.906098 | 0.778802 |
| 22 | 1 | 0 | 2.713162 | 3.499662 | 1.105874 |
| 23 | 1 | 0 | 1.329274 | 3.572813 | 0.028042 |
| 24 | 1 | 0 | -3.449232 | 2.187337 | -1.598376 |
| 25 | 6 | 0 | 2.504929 | 1.774627 | -0.035446 |
| 26 | 8 | 0 | 3.691898 | 1.809796 | -0.348648 |
| 27 | 7 | 0 | 1.748662 | 0.621419 | -0.312614 |
| 28 | 6 | 0 | 2.458086 | -0.605555 | -0.557301 |

| | | | | | |
|---|---|---|---|---|---|
| 29 | 6 | 0 | 3.270973 | -1.142496 | 0.448351 |
| 30 | 6 | 0 | 2.321699 | -1.266705 | -1.781338 |
| 31 | 6 | 0 | 3.955286 | -2.335804 | 0.216418 |
| 32 | 6 | 0 | 3.000738 | -2.466666 | -2.001106 |
| 33 | 1 | 0 | 1.687979 | -0.840419 | -2.552951 |
| 34 | 6 | 0 | 3.822349 | -3.001175 | -1.005964 |
| 35 | 1 | 0 | 4.585658 | -2.753578 | 0.996204 |
| 36 | 1 | 0 | 2.892709 | -2.978715 | -2.952738 |
| 37 | 1 | 0 | 4.353187 | -3.932323 | -1.180384 |
| 38 | 1 | 0 | 3.335384 | -0.618261 | 1.398634 |
| 39 | 8 | 0 | 2.367688 | 0.806588 | 3.143027 |
| 40 | 1 | 0 | 1.808502 | 1.494583 | 2.600189 |
| 41 | 1 | 0 | 1.826192 | 0.008683 | 3.098306 |

**TS19a**

| Center | Atomic | Atomic | Coordinates (Angstroms) | | |
|---|---|---|---|---|---|
| Number | Number | Type | X | Y | Z |
| 1 | 6 | 0 | 0.746819 | -0.032989 | 0.019252 |
| 2 | 6 | 0 | 1.894299 | 0.760356 | -0.117899 |
| 3 | 6 | 0 | 3.137509 | 0.136567 | -0.104069 |
| 4 | 6 | 0 | 3.292733 | -1.248466 | 0.044248 |
| 5 | 6 | 0 | 2.116550 | -2.033780 | 0.182000 |
| 6 | 6 | 0 | 0.862703 | -1.429481 | 0.161619 |
| 7 | 1 | 0 | 1.841918 | 1.832761 | -0.237921 |
| 8 | 6 | 0 | 4.618573 | -1.846210 | 0.050825 |
| 9 | 1 | 0 | -0.014068 | -2.051585 | 0.273618 |
| 10 | 6 | 0 | 5.712054 | -0.912942 | -0.099419 |
| 11 | 6 | 0 | 5.486693 | 0.418332 | -0.237629 |
| 12 | 1 | 0 | 6.727367 | -1.289383 | -0.101861 |
| 13 | 6 | 0 | 6.529219 | 1.473280 | -0.399717 |
| 14 | 1 | 0 | 7.526315 | 1.032695 | -0.390725 |
| 15 | 1 | 0 | 6.382238 | 2.008616 | -1.343465 |
| 16 | 1 | 0 | 6.454132 | 2.206919 | 0.409567 |
| 17 | 8 | 0 | 4.784412 | -3.085654 | 0.179285 |
| 18 | 8 | 0 | 2.213346 | -3.366837 | 0.322549 |
| 19 | 8 | 0 | -0.788875 | 1.370689 | 3.241233 |
| 20 | 8 | 0 | 4.234884 | 0.947668 | -0.243613 |
| 21 | 6 | 0 | -0.849360 | 0.100940 | 2.613961 |
| 22 | 1 | 0 | -1.410484 | -0.621507 | 3.224099 |
| 23 | 1 | 0 | 0.165790 | -0.276355 | 2.490008 |
| 24 | 1 | 0 | 3.192041 | -3.570488 | 0.301802 |
| 25 | 6 | 0 | -1.618954 | 0.244044 | 1.283365 |
| 26 | 8 | 0 | -2.574708 | 1.067509 | 1.282212 |
| 27 | 7 | 0 | -0.573530 | 0.541454 | -0.019901 |
| 28 | 6 | 0 | -0.649772 | 1.922921 | -0.474119 |
| 29 | 6 | 0 | -0.433517 | 2.998456 | 0.394578 |
| 30 | 6 | 0 | -0.928955 | 2.162571 | -1.822519 |
| 31 | 6 | 0 | -0.502493 | 4.304841 | -0.089473 |
| 32 | 6 | 0 | -0.995702 | 3.472878 | -2.302212 |
| 33 | 1 | 0 | -1.087146 | 1.328719 | -2.499276 |
| 34 | 6 | 0 | -0.784246 | 4.546748 | -1.436953 |
| 35 | 1 | 0 | -0.335188 | 5.135892 | 0.589130 |
| 36 | 1 | 0 | -1.212124 | 3.649647 | -3.351384 |
| 37 | 1 | 0 | -0.836685 | 5.565490 | -1.808673 |
| 38 | 1 | 0 | -0.225721 | 2.802871 | 1.440645 |
| 39 | 1 | 0 | -1.630295 | 1.791516 | 2.984041 |
| 40 | 6 | 0 | -2.710776 | -1.419778 | -0.197527 |
| 41 | 8 | 0 | -3.728273 | -2.116588 | -0.226619 |
| 42 | 8 | 0 | -2.162149 | -0.883888 | -1.252375 |
| 43 | 8 | 0 | -2.092219 | -1.191758 | 0.989012 |
| 44 | 1 | 0 | -1.326050 | -0.193546 | -0.831770 |
| 45 | 19 | 0 | -4.077027 | -2.035767 | -2.996956 |
| 46 | 19 | 0 | -4.808707 | -0.426407 | 1.936331 |

**12**

| Center | Atomic | Atomic | Coordinates (Angstroms) | | |
|---|---|---|---|---|---|
| Number | Number | Type | X | Y | Z |
| 1 | 6 | 0 | -1.146896 | 0.430925 | 0.282761 |
| 2 | 6 | 0 | -2.487581 | 0.869785 | 0.281197 |
| 3 | 6 | 0 | -3.494413 | -0.081029 | 0.203203 |
| 4 | 6 | 0 | -3.247045 | -1.463213 | 0.131561 |
| 5 | 6 | 0 | -1.886431 | -1.887441 | 0.150061 |
| 6 | 6 | 0 | -0.863069 | -0.956480 | 0.221987 |
| 7 | 1 | 0 | -2.751267 | 1.913960 | 0.369537 |
| 8 | 6 | 0 | -4.340224 | -2.406642 | 0.053289 |
| 9 | 1 | 0 | 0.165582 | -1.296719 | 0.221828 |
| 10 | 6 | 0 | -5.663745 | -1.818723 | 0.060508 |
| 11 | 6 | 0 | -5.834138 | -0.476065 | 0.140169 |
| 12 | 1 | 0 | -6.527909 | -2.469232 | 0.006243 |
| 13 | 6 | 0 | -7.140680 | 0.245215 | 0.163704 |
| 14 | 1 | 0 | -7.970193 | -0.460135 | 0.107437 |
| 15 | 1 | 0 | -7.233019 | 0.833022 | 1.082924 |
| 16 | 1 | 0 | -7.205277 | 0.941394 | -0.678980 |
| 17 | 8 | 0 | -4.146851 | -3.651027 | -0.010850 |
| 18 | 8 | 0 | -1.602603 | -3.202991 | 0.087272 |
| 19 | 8 | 0 | 2.300445 | 1.014862 | -3.880607 |
| 20 | 8 | 0 | -4.785905 | 0.388063 | 0.209356 |
| 21 | 6 | 0 | 2.044988 | -0.052124 | -2.998310 |
| 22 | 1 | 0 | 2.410458 | -0.990178 | -3.431119 |
| 23 | 1 | 0 | 0.968558 | -0.184487 | -2.801681 |
| 24 | 1 | 0 | -2.489587 | -3.664859 | 0.035741 |
| 25 | 6 | 0 | 2.719826 | 0.181368 | -1.657871 |
| 26 | 8 | 0 | 3.229133 | 1.245051 | -1.362263 |
| 27 | 7 | 0 | -0.069420 | 1.286888 | 0.381138 |
| 28 | 6 | 0 | -0.030705 | 2.686224 | 0.262901 |
| 29 | 6 | 0 | -0.858812 | 3.407657 | -0.614613 |
| 30 | 6 | 0 | 0.936919 | 3.384152 | 1.008760 |
| 31 | 6 | 0 | -0.737096 | 4.794086 | -0.711843 |
| 32 | 6 | 0 | 1.060015 | 4.766452 | 0.890751 |
| 33 | 1 | 0 | 1.589291 | 2.830170 | 1.677423 |
| 34 | 6 | 0 | 0.217074 | 5.484333 | 0.038023 |
| 35 | 1 | 0 | -1.386958 | 5.333845 | -1.394640 |
| 36 | 1 | 0 | 1.814270 | 5.285028 | 1.475668 |
| 37 | 1 | 0 | 0.307645 | 6.562686 | -0.045689 |
| 38 | 1 | 0 | -1.575289 | 2.886385 | -1.238334 |
| 39 | 1 | 0 | 2.568809 | 1.761684 | -3.319445 |
| 40 | 6 | 0 | 3.117386 | -0.758094 | 0.548598 |
| 41 | 8 | 0 | 4.074211 | -1.495041 | 0.815578 |
| 42 | 8 | 0 | 2.430023 | -0.000149 | 1.243011 |
| 43 | 8 | 0 | 2.672123 | -0.900787 | -0.865404 |
| 44 | 1 | 0 | 0.811129 | 0.849935 | 0.660908 |
| 45 | 19 | 0 | 3.976795 | -0.599682 | 3.497060 |
| 46 | 19 | 0 | 4.414408 | -3.317522 | -1.181984 |

**3**

| Center | Atomic | Atomic | Coordinates (Angstroms) | | |
|---|---|---|---|---|---|
| Number | Number | Type | X | Y | Z |
| 1 | 6 | 0 | 2.650643 | 0.675492 | 0.380240 |
| 2 | 1 | 0 | 2.831968 | 0.827381 | 1.446178 |
| 3 | 1 | 0 | 2.521258 | 1.644589 | -0.101057 |
| 4 | 6 | 0 | 1.432315 | -0.241947 | 0.201703 |
| 5 | 8 | 0 | 1.524990 | -1.462234 | 0.223959 |
| 6 | 7 | 0 | 0.268933 | 0.465325 | 0.076374 |
| 7 | 17 | 0 | 4.146849 | -0.061560 | -0.305886 |
| 8 | 1 | 0 | 0.365867 | 1.470084 | 0.048311 |
| 9 | 6 | 0 | -1.065004 | -0.008570 | 0.019774 |
| 10 | 6 | 0 | -2.096175 | 0.959022 | -0.036022 |
| 11 | 6 | 0 | -1.373891 | -1.376773 | 0.014273 |
| 12 | 6 | 0 | -3.418194 | 0.508914 | -0.096317 |
| 13 | 6 | 0 | -2.705208 | -1.788731 | -0.044714 |
| 14 | 1 | 0 | -0.571385 | -2.098336 | 0.055717 |
| 15 | 6 | 0 | -3.734015 | -0.850585 | -0.100908 |
| 16 | 1 | 0 | -4.214644 | 1.246865 | -0.139364 |
| 17 | 1 | 0 | -2.930302 | -2.851099 | -0.048213 |
| 18 | 1 | 0 | -4.770790 | -1.168872 | -0.148231 |
| 19 | 6 | 0 | -1.796683 | 2.440565 | -0.030137 |
| 20 | 1 | 0 | -1.258621 | 2.750635 | 0.874672 |
| 21 | 1 | 0 | -1.191565 | 2.744866 | -0.893727 |
| 22 | 1 | 0 | -2.723394 | 3.016689 | -0.067871 |

**TS3**

| Center | Atomic | Atomic | Coordinates (Angstroms) | | |
|---|---|---|---|---|---|
| Number | Number | Type | X | Y | Z |
| 1 | 6 | 0 | -1.005037 | 1.075396 | 0.478019 |
| 2 | 6 | 0 | -1.531827 | -0.220280 | 0.191709 |
| 3 | 6 | 0 | -2.866023 | -0.348013 | -0.142294 |
| 4 | 6 | 0 | -3.755480 | 0.745858 | -0.223195 |
| 5 | 6 | 0 | -3.221962 | 2.042105 | 0.056516 |
| 6 | 6 | 0 | -1.891501 | 2.196186 | 0.400308 |
| 7 | 1 | 0 | -0.882335 | -1.087208 | 0.194916 |
| 8 | 6 | 0 | -5.136448 | 0.549777 | -0.579179 |
| 9 | 1 | 0 | -1.502579 | 3.185004 | 0.619262 |
| 10 | 6 | 0 | -5.521426 | -0.824187 | -0.842170 |
| 11 | 6 | 0 | -4.621141 | -1.833024 | -0.754467 |
| 12 | 1 | 0 | -6.547369 | -1.038309 | -1.115914 |
| 13 | 6 | 0 | -4.895451 | -3.279870 | -1.005220 |
| 14 | 1 | 0 | -5.941996 | -3.432688 | -1.271019 |
| 15 | 1 | 0 | -4.263234 | -3.652243 | -1.818252 |
| 16 | 1 | 0 | -4.663677 | -3.871515 | -0.113336 |
| 17 | 8 | 0 | -5.959833 | 1.506471 | -0.660760 |
| 18 | 8 | 0 | -4.042136 | 3.115626 | -0.009509 |
| 19 | 8 | 0 | 0.240558 | 1.276033 | 0.796104 |
| 20 | 8 | 0 | -3.322409 | -1.622422 | -0.415294 |
| 21 | 6 | 0 | 1.209383 | -0.140158 | 1.991677 |
| 22 | 1 | 0 | 0.255356 | -0.590570 | 2.201687 |
| 23 | 1 | 0 | 1.499966 | 0.747250 | 2.526318 |
| 24 | 1 | 0 | -4.936923 | 2.748107 | -0.265860 |
| 25 | 6 | 0 | 1.940055 | -0.611900 | 0.769396 |
| 26 | 8 | 0 | 1.545174 | -1.580441 | 0.122446 |
| 27 | 7 | 0 | 3.055732 | 0.127605 | 0.476445 |
| 28 | 17 | 0 | 2.252220 | -1.466937 | 3.471716 |
| 29 | 1 | 0 | 3.228748 | 0.905572 | 1.095370 |
| 30 | 6 | 0 | 3.972113 | -0.020448 | -0.586402 |
| 31 | 6 | 0 | 5.008398 | 0.938486 | -0.697221 |
| 32 | 6 | 0 | 3.885558 | -1.074444 | -1.509695 |
| 33 | 6 | 0 | 5.927128 | 0.801226 | -1.741582 |
| 34 | 6 | 0 | 4.820128 | -1.179542 | -2.539457 |
| 35 | 1 | 0 | 3.086185 | -1.793521 | -1.408973 |
| 36 | 6 | 0 | 5.846073 | -0.244427 | -2.662857 |
| 37 | 1 | 0 | 6.723287 | 1.535837 | -1.829514 |

| | | | | | |
|---|---|---|---|---|---|
| 38 | 1 | 0 | 4.738682 | -2.001023 | -3.245347 |
| 39 | 1 | 0 | 6.574788 | -0.323735 | -3.463657 |
| 40 | 6 | 0 | 5.130204 | 2.087685 | 0.276133 |
| 41 | 1 | 0 | 4.252869 | 2.746090 | 0.248513 |
| 42 | 1 | 0 | 5.250556 | 1.743988 | 1.311469 |
| 43 | 1 | 0 | 6.002507 | 2.698954 | 0.036050 |

**8**

| Center Number | Atomic Number | Atomic Type | Coordinates (Angstroms) X | Y | Z |
|---|---|---|---|---|---|
| 1 | 6 | 0 | 0.644964 | -1.365016 | -0.277642 |
| 2 | 6 | 0 | 1.180812 | -0.635511 | 0.789665 |
| 3 | 6 | 0 | 2.459543 | -0.107594 | 0.626807 |
| 4 | 6 | 0 | 3.211807 | -0.275415 | -0.544555 |
| 5 | 6 | 0 | 2.630876 | -1.020152 | -1.611919 |
| 6 | 6 | 0 | 1.357260 | -1.557977 | -1.472808 |
| 7 | 1 | 0 | 0.649330 | -0.456431 | 1.713822 |
| 8 | 6 | 0 | 4.540333 | 0.301999 | -0.653344 |
| 9 | 1 | 0 | 0.913156 | -2.131083 | -2.277822 |
| 10 | 6 | 0 | 4.989916 | 1.035640 | 0.509195 |
| 11 | 6 | 0 | 4.205540 | 1.159273 | 1.608850 |
| 12 | 1 | 0 | 5.971431 | 1.492873 | 0.493818 |
| 13 | 6 | 0 | 4.554537 | 1.888752 | 2.862659 |
| 14 | 1 | 0 | 5.549670 | 2.327471 | 2.787377 |
| 15 | 1 | 0 | 3.825963 | 2.683481 | 3.053386 |
| 16 | 1 | 0 | 4.527711 | 1.205790 | 3.718023 |
| 17 | 8 | 0 | 5.239323 | 0.167357 | -1.690468 |
| 18 | 8 | 0 | 3.320331 | -1.202451 | -2.749880 |
| 19 | 8 | 0 | -0.596338 | -1.935713 | -0.258242 |
| 20 | 8 | 0 | 2.965620 | 0.605214 | 1.682076 |
| 21 | 6 | 0 | -1.368247 | -1.901799 | 0.946949 |
| 22 | 1 | 0 | -0.755127 | -2.116969 | 1.825618 |
| 23 | 1 | 0 | -2.112304 | -2.702787 | 0.836977 |
| 24 | 1 | 0 | 4.194384 | -0.734601 | -2.613707 |
| 25 | 6 | 0 | -2.106748 | -0.581142 | 1.182744 |
| 26 | 8 | 0 | -2.497550 | -0.298346 | 2.313201 |
| 27 | 7 | 0 | -2.310507 | 0.152690 | 0.051479 |
| 28 | 17 | 0 | -3.733514 | -4.707453 | 0.470814 |
| 29 | 1 | 0 | -1.918955 | -0.260386 | -0.784700 |
| 30 | 6 | 0 | -2.966641 | 1.393991 | -0.110905 |
| 31 | 6 | 0 | -2.964298 | 1.971088 | -1.402972 |
| 32 | 6 | 0 | -3.603043 | 2.046059 | 0.955921 |
| 33 | 6 | 0 | -3.609628 | 3.198815 | -1.577265 |
| 34 | 6 | 0 | -4.235922 | 3.271066 | 0.745182 |
| 35 | 1 | 0 | -3.592715 | 1.586418 | 1.933612 |
| 36 | 6 | 0 | -4.243644 | 3.854395 | -0.520530 |
| 37 | 1 | 0 | -3.610954 | 3.647126 | -2.567196 |
| 38 | 1 | 0 | -4.724595 | 3.764988 | 1.579916 |
| 39 | 1 | 0 | -4.735809 | 4.807567 | -0.687353 |
| 40 | 6 | 0 | -2.287628 | 1.291890 | -2.571053 |
| 41 | 1 | 0 | -1.219024 | 1.122748 | -2.388649 |
| 42 | 1 | 0 | -2.738359 | 0.317697 | -2.800016 |
| 43 | 1 | 0 | -2.370337 | 1.905315 | -3.470668 |

**TS17b**

| Center Number | Atomic Number | Atomic Type | Coordinates (Angstroms) X | Y | Z |
|---|---|---|---|---|---|
| 1 | 6 | 0 | 0.562986 | -1.501804 | -0.628126 |
| 2 | 6 | 0 | -0.464606 | -0.842750 | -1.400524 |
| 3 | 6 | 0 | -1.619275 | -0.422436 | -0.792787 |
| 4 | 6 | 0 | -1.925865 | -0.671353 | 0.571193 |
| 5 | 6 | 0 | -0.956504 | -1.427015 | 1.315485 |
| 6 | 6 | 0 | 0.208143 | -1.863239 | 0.734512 |
| 7 | 1 | 0 | -0.293385 | -0.655774 | -2.453458 |
| 8 | 6 | 0 | -3.156286 | -0.237038 | 1.143549 |
| 9 | 1 | 0 | 0.903208 | -2.452470 | 1.324143 |
| 10 | 6 | 0 | -4.045706 | 0.483387 | 0.241666 |
| 11 | 6 | 0 | -3.711148 | 0.691572 | -1.051980 |
| 12 | 1 | 0 | -4.994464 | 0.846023 | 0.619063 |
| 13 | 6 | 0 | -4.538153 | 1.402804 | -2.073710 |
| 14 | 1 | 0 | -5.479154 | 1.742049 | -1.638671 |
| 15 | 1 | 0 | -3.997012 | 2.269217 | -2.469192 |
| 16 | 1 | 0 | -4.755743 | 0.740797 | -2.918872 |
| 17 | 8 | 0 | -3.477028 | -0.461521 | 2.354144 |
| 18 | 8 | 0 | -1.239368 | -1.730514 | 2.608412 |
| 19 | 8 | 0 | 1.247767 | -2.484003 | -1.391679 |
| 20 | 8 | 0 | -2.533617 | 0.256641 | -1.581179 |
| 21 | 6 | 0 | 2.608478 | -2.626190 | -1.014087 |
| 22 | 1 | 0 | 3.172558 | -2.970970 | -1.885989 |
| 23 | 1 | 0 | 2.744073 | -3.364642 | -0.208919 |
| 24 | 1 | 0 | -2.137213 | -1.317635 | 2.779708 |
| 25 | 6 | 0 | 3.111686 | -1.267236 | -0.527228 |
| 26 | 8 | 0 | 4.297993 | -1.075867 | -0.212222 |
| 27 | 7 | 0 | 2.070120 | -0.429534 | -0.510215 |
| 28 | 6 | 0 | 2.082795 | 0.810366 | 0.151764 |
| 29 | 6 | 0 | 1.891119 | 2.008359 | -0.576056 |
| 30 | 6 | 0 | 2.232562 | 0.865705 | 1.548433 |
| 31 | 6 | 0 | 1.851767 | 3.217917 | 0.127900 |
| 32 | 6 | 0 | 2.189005 | 2.082007 | 2.229498 |
| 33 | 1 | 0 | 2.383182 | -0.062020 | 2.090390 |

| | | | | | |
|---|---|---|---|---|---|
| 34 | 6 | 0 | 1.993733 | 3.265509 | 1.516955 |
| 35 | 1 | 0 | 1.711955 | 4.142313 | -0.427594 |
| 36 | 1 | 0 | 2.305707 | 2.102984 | 3.309520 |
| 37 | 1 | 0 | 1.957652 | 4.219968 | 2.034385 |
| 38 | 6 | 0 | 1.759843 | 1.993687 | -2.080214 |
| 39 | 1 | 0 | 2.571439 | 1.424205 | -2.544571 |
| 40 | 1 | 0 | 0.824445 | 1.520579 | -2.394071 |
| 41 | 1 | 0 | 1.778492 | 3.010717 | -2.481641 |

**18b**

| Center Number | Atomic Number | Atomic Type | Coordinates (Angstroms) X | Y | Z |
|---|---|---|---|---|---|
| 1 | 6 | 0 | 0.504824 | -1.245887 | -0.499691 |
| 2 | 6 | 0 | -0.345182 | -0.260577 | -1.201204 |
| 3 | 6 | 0 | -1.607957 | 0.019610 | -0.771476 |
| 4 | 6 | 0 | -2.211934 | -0.566002 | 0.380856 |
| 5 | 6 | 0 | -1.399765 | -1.510981 | 1.107254 |
| 6 | 6 | 0 | -0.126955 | -1.815205 | 0.718328 |
| 7 | 1 | 0 | 0.046634 | 0.211414 | -2.095348 |
| 8 | 6 | 0 | -3.528993 | -0.233318 | 0.777704 |
| 9 | 1 | 0 | 0.448708 | -2.534491 | 1.294575 |
| 10 | 6 | 0 | -4.213403 | 0.742258 | -0.067811 |
| 11 | 6 | 0 | -3.606469 | 1.275441 | -1.150258 |
| 12 | 1 | 0 | -5.224848 | 1.037996 | 0.185513 |
| 13 | 6 | 0 | -4.196520 | 2.278948 | -2.088599 |
| 14 | 1 | 0 | -5.211187 | 2.539946 | -1.784215 |
| 15 | 1 | 0 | -3.588086 | 3.189850 | -2.110421 |
| 16 | 1 | 0 | -4.223987 | 1.882291 | -3.109537 |
| 17 | 8 | 0 | -4.106243 | -0.736416 | 1.801409 |
| 18 | 8 | 0 | -1.948083 | -2.091293 | 2.211125 |
| 19 | 8 | 0 | 0.871578 | -2.333659 | -1.457623 |
| 20 | 8 | 0 | -2.336254 | 0.940175 | -1.517031 |
| 21 | 6 | 0 | 2.222316 | -2.710472 | -1.290071 |
| 22 | 1 | 0 | 2.690383 | -2.898236 | -2.263700 |
| 23 | 1 | 0 | 2.344765 | -3.618290 | -0.676905 |
| 24 | 1 | 0 | -2.869188 | -1.694686 | 2.275487 |
| 25 | 6 | 0 | 2.900866 | -1.540047 | -0.595804 |
| 26 | 8 | 0 | 4.112517 | -1.412550 | -0.412678 |
| 27 | 7 | 0 | 1.908987 | -0.696612 | -0.222393 |
| 28 | 6 | 0 | 2.137181 | 0.537389 | 0.455189 |
| 29 | 6 | 0 | 2.784247 | 1.599392 | -0.212318 |
| 30 | 6 | 0 | 1.708275 | 0.689493 | 1.779436 |
| 31 | 6 | 0 | 2.987387 | 2.791877 | 0.495212 |
| 32 | 6 | 0 | 1.909879 | 1.891608 | 2.456906 |
| 33 | 1 | 0 | 1.211055 | -0.142407 | 2.264228 |
| 34 | 6 | 0 | 2.556321 | 2.946765 | 1.813140 |
| 35 | 1 | 0 | 3.487074 | 3.616749 | -0.006149 |
| 36 | 1 | 0 | 1.571257 | 1.996648 | 3.483379 |
| 37 | 1 | 0 | 2.724113 | 3.886418 | 2.331412 |
| 38 | 6 | 0 | 3.252424 | 1.472017 | -1.640260 |
| 39 | 1 | 0 | 4.096348 | 0.778364 | -1.713319 |
| 40 | 1 | 0 | 2.460317 | 1.078691 | -2.285534 |
| 41 | 1 | 0 | 3.566368 | 2.442124 | -2.033534 |

**TS18b**

| Center Number | Atomic Number | Atomic Type | Coordinates (Angstroms) X | Y | Z |
|---|---|---|---|---|---|
| 1 | 6 | 0 | -0.337393 | -0.901228 | -0.165735 |
| 2 | 6 | 0 | 0.395210 | -0.068673 | 0.746889 |
| 3 | 6 | 0 | 1.733319 | 0.158841 | 0.531284 |
| 4 | 6 | 0 | 2.430559 | -0.298022 | -0.614603 |
| 5 | 6 | 0 | 1.664738 | -1.019285 | -1.588432 |
| 6 | 6 | 0 | 0.322846 | -1.258504 | -1.398669 |
| 7 | 1 | 0 | -0.090720 | 0.318240 | 1.633140 |
| 8 | 6 | 0 | 3.817921 | -0.003856 | -0.801069 |
| 9 | 1 | 0 | -0.237062 | -1.787731 | -2.162892 |
| 10 | 6 | 0 | 4.441080 | 0.765985 | 0.264655 |
| 11 | 6 | 0 | 3.727818 | 1.181602 | 1.336989 |
| 12 | 1 | 0 | 5.492584 | 1.014966 | 0.186649 |
| 13 | 6 | 0 | 4.249761 | 1.982919 | 2.485141 |
| 14 | 1 | 0 | 5.308927 | 2.204906 | 2.349176 |
| 15 | 1 | 0 | 3.697763 | 2.924523 | 2.576429 |
| 16 | 1 | 0 | 4.118945 | 1.435324 | 3.424692 |
| 17 | 8 | 0 | 4.469796 | -0.377685 | -1.824026 |
| 18 | 8 | 0 | 2.285016 | -1.433764 | -2.720724 |
| 19 | 8 | 0 | -0.617744 | -2.519197 | 0.717837 |
| 20 | 8 | 0 | 2.404706 | 0.900204 | 1.487046 |
| 21 | 6 | 0 | -1.880491 | -2.927326 | 0.310314 |
| 22 | 1 | 0 | -2.473923 | -3.376126 | 1.124807 |
| 23 | 1 | 0 | -1.868696 | -3.668400 | -0.511912 |
| 24 | 1 | 0 | 3.232909 | -1.123540 | -2.625644 |
| 25 | 6 | 0 | -2.645073 | -1.699041 | -0.172421 |
| 26 | 8 | 0 | -3.848077 | -1.661846 | -0.425020 |
| 27 | 7 | 0 | -1.783351 | -0.639110 | -0.255455 |
| 28 | 6 | 0 | -2.245978 | 0.686151 | -0.537757 |
| 29 | 6 | 0 | -3.098969 | 1.342836 | 0.374655 |
| 30 | 6 | 0 | -1.825002 | 1.328138 | -1.708574 |
| 31 | 6 | 0 | -3.516261 | 2.642789 | 0.053722 |
| 32 | 6 | 0 | -2.245691 | 2.624903 | -1.997215 |
| 33 | 1 | 0 | -1.160261 | 0.800187 | -2.383669 |

| | | | | | |
|---|---|---|---|---|---|
| 34 | 6 | 0 | -3.100854 | 3.284178 | -1.112997 |
| 35 | 1 | 0 | -4.173404 | 3.163096 | 0.745880 |
| 36 | 1 | 0 | -1.911705 | 3.111823 | -2.908563 |
| 37 | 1 | 0 | -3.438589 | 4.294210 | -1.326061 |
| 38 | 6 | 0 | -3.563885 | 0.691865 | 1.653779 |
| 39 | 1 | 0 | -4.385007 | -0.004940 | 1.452212 |
| 40 | 1 | 0 | -2.770038 | 0.108995 | 2.132416 |
| 41 | 1 | 0 | -3.921261 | 1.446073 | 2.360145 |
| 42 | 8 | 0 | -1.144269 | -1.696405 | 3.251223 |
| 43 | 1 | 0 | -0.884271 | -2.002186 | 2.342264 |
| 44 | 1 | 0 | -0.390089 | -1.161734 | 3.528696 |

**19b**

| Center Number | Atomic Number | Atomic Type | Coordinates (Angstroms) X | Y | Z |
|---|---|---|---|---|---|
| 1 | 6 | 0 | -0.251884 | -0.582083 | -0.286630 |
| 2 | 6 | 0 | 0.408399 | 0.448424 | 0.398053 |
| 3 | 6 | 0 | 1.788472 | 0.541167 | 0.281389 |
| 4 | 6 | 0 | 2.543118 | -0.348926 | -0.501798 |
| 5 | 6 | 0 | 1.846996 | -1.375493 | -1.200409 |
| 6 | 6 | 0 | 0.463588 | -1.470908 | -1.105617 |
| 7 | 1 | 0 | -0.136565 | 1.162178 | 1.002829 |
| 8 | 6 | 0 | 3.984461 | -0.202741 | -0.595479 |
| 9 | 1 | 0 | -0.053723 | -2.244193 | -1.660473 |
| 10 | 6 | 0 | 4.543764 | 0.891256 | 0.168282 |
| 11 | 6 | 0 | 3.753657 | 1.711652 | 0.905406 |
| 12 | 1 | 0 | 5.614205 | 1.053815 | 0.144756 |
| 13 | 6 | 0 | 4.214497 | 2.868874 | 1.727572 |
| 14 | 1 | 0 | 5.296914 | 2.980724 | 1.660768 |
| 15 | 1 | 0 | 3.737112 | 3.792191 | 1.383299 |
| 16 | 1 | 0 | 3.933434 | 2.725046 | 2.775994 |
| 17 | 8 | 0 | 4.688631 | -0.977052 | -1.294792 |
| 18 | 8 | 0 | 2.531411 | -2.240164 | -1.974377 |
| 19 | 8 | 0 | -0.666276 | -2.778774 | 1.348268 |
| 20 | 8 | 0 | 2.405814 | 1.556848 | 0.970856 |
| 21 | 6 | 0 | -1.749087 | -3.008678 | 0.578673 |
| 22 | 1 | 0 | -2.627720 | -3.476485 | 1.090152 |
| 23 | 1 | 0 | -1.561874 | -3.707310 | -0.280015 |
| 24 | 1 | 0 | 3.489582 | -1.967245 | -1.898657 |
| 25 | 6 | 0 | -2.426204 | -1.788353 | -0.096661 |
| 26 | 8 | 0 | -3.610596 | -1.810764 | -0.429712 |
| 27 | 7 | 0 | -1.675892 | -0.618070 | -0.255742 |
| 28 | 6 | 0 | -2.364146 | 0.603246 | -0.589798 |
| 29 | 6 | 0 | -3.288223 | 1.181870 | 0.303889 |
| 30 | 6 | 0 | -2.074301 | 1.218355 | -1.813941 |
| 31 | 6 | 0 | -3.900223 | 2.382580 | -0.086512 |
| 32 | 6 | 0 | -2.691206 | 2.414132 | -2.174126 |
| 33 | 1 | 0 | -1.357308 | 0.748154 | -2.479916 |
| 34 | 6 | 0 | -3.612912 | 2.998855 | -1.303985 |
| 35 | 1 | 0 | -4.613112 | 2.844522 | 0.591763 |
| 36 | 1 | 0 | -2.456937 | 2.879996 | -3.126529 |
| 37 | 1 | 0 | -4.102841 | 3.931282 | -1.568677 |
| 38 | 6 | 0 | -3.640947 | 0.549637 | 1.626152 |
| 39 | 1 | 0 | -4.401617 | -0.226533 | 1.482470 |
| 40 | 1 | 0 | -2.783034 | 0.063205 | 2.103333 |
| 41 | 1 | 0 | -4.048870 | 1.298313 | 2.311379 |
| 42 | 8 | 0 | -1.094635 | -1.183031 | 3.294825 |
| 43 | 1 | 0 | -0.952858 | -1.841730 | 2.508048 |
| 44 | 1 | 0 | -0.565252 | -0.423150 | 3.022367 |

**TS19b**

| Center Number | Atomic Number | Atomic Type | Coordinates (Angstroms) X | Y | Z |
|---|---|---|---|---|---|
| 1 | 6 | 0 | 0.637717 | 0.143051 | -0.177650 |
| 2 | 6 | 0 | 1.835454 | 0.645472 | 0.341353 |
| 3 | 6 | 0 | 3.003837 | -0.072244 | 0.106109 |
| 4 | 6 | 0 | 3.020875 | -1.268039 | -0.626703 |
| 5 | 6 | 0 | 1.791964 | -1.749436 | -1.150941 |
| 6 | 6 | 0 | 0.611722 | -1.040968 | -0.927241 |
| 7 | 1 | 0 | 1.874733 | 1.567550 | 0.905693 |
| 8 | 6 | 0 | 4.273566 | -1.980115 | -0.843209 |
| 9 | 1 | 0 | -0.309983 | -1.409906 | -1.354924 |
| 10 | 6 | 0 | 5.437897 | -1.367313 | -0.247346 |
| 11 | 6 | 0 | 5.343324 | -0.207999 | 0.452602 |
| 12 | 1 | 0 | 6.401764 | -1.846441 | -0.365238 |
| 13 | 6 | 0 | 6.470206 | 0.514206 | 1.110942 |
| 14 | 1 | 0 | 7.409574 | -0.016365 | 0.954952 |
| 15 | 1 | 0 | 6.558369 | 1.527304 | 0.705375 |
| 16 | 1 | 0 | 6.283713 | 0.605252 | 2.185958 |
| 17 | 8 | 0 | 4.317829 | -3.049211 | -1.501214 |
| 18 | 8 | 0 | 1.763905 | -2.882949 | -1.869704 |
| 19 | 8 | 0 | -1.438097 | -0.615425 | 3.572673 |
| 20 | 8 | 0 | 4.161814 | 0.439331 | 0.631529 |
| 21 | 6 | 0 | -0.576416 | -0.262330 | 2.493435 |
| 22 | 1 | 0 | -0.015510 | -1.149729 | 2.201266 |
| 23 | 1 | 0 | 0.132948 | 0.519651 | 2.795194 |
| 24 | 1 | 0 | 2.709701 | -3.207396 | -1.901295 |
| 25 | 6 | 0 | -1.471141 | 0.275936 | 1.361646 |
| 26 | 8 | 0 | -2.412056 | 1.026889 | 1.685361 |
| 27 | 7 | 0 | -0.618557 | 0.839085 | 0.071048 |
| 28 | 6 | 0 | -0.499640 | 2.301267 | 0.013871 |
| 29 | 6 | 0 | -0.429729 | 2.917989 | -1.253348 |
| 30 | 6 | 0 | -0.425351 | 3.070879 | 1.179681 |
| 31 | 6 | 0 | -0.287601 | 4.312296 | -1.295474 |
| 32 | 6 | 0 | -0.288831 | 4.456725 | 1.107589 |
| 33 | 1 | 0 | -0.497915 | 2.595619 | 2.148018 |
| 34 | 6 | 0 | -0.220179 | 5.082331 | -0.135693 |
| 35 | 1 | 0 | -0.113180 | 6.160523 | -0.204769 |
| 36 | 1 | 0 | -2.088791 | 0.110760 | 3.590953 |
| 37 | 6 | 0 | -2.813956 | -1.030486 | -0.329796 |
| 38 | 8 | 0 | -3.767738 | -1.815723 | -0.438862 |
| 39 | 8 | 0 | -2.514005 | -0.112538 | -1.208250 |
| 40 | 8 | 0 | -1.995057 | -1.123104 | 0.730517 |
| 41 | 1 | 0 | -1.525845 | 0.448876 | -0.731073 |
| 42 | 19 | 0 | -4.676361 | -0.809755 | -2.842269 |
| 43 | 19 | 0 | -3.483792 | -2.540838 | 2.467835 |
| 44 | 1 | 0 | -0.235670 | 5.037821 | 2.022856 |
| 45 | 1 | 0 | -0.228744 | 4.797464 | -2.265774 |
| 46 | 6 | 0 | -0.484148 | 2.140363 | -2.548344 |
| 47 | 1 | 0 | -0.388609 | 2.819260 | -3.398603 |
| 48 | 1 | 0 | -1.426705 | 1.596312 | -2.659971 |
| 49 | 1 | 0 | 0.322772 | 1.403433 | -2.617009 |

**13**

| Center Number | Atomic Number | Atomic Type | Coordinates (Angstroms) X | Y | Z |
|---|---|---|---|---|---|
| 1 | 6 | 0 | -1.175336 | 0.221899 | 0.473671 |
| 2 | 6 | 0 | -2.457245 | 0.791098 | 0.318710 |
| 3 | 6 | 0 | -3.534800 | -0.055134 | 0.108562 |
| 4 | 6 | 0 | -3.415606 | -1.455223 | 0.048272 |
| 5 | 6 | 0 | -2.114140 | -2.010719 | 0.221765 |
| 6 | 6 | 0 | -1.019834 | -1.185779 | 0.426756 |
| 7 | 1 | 0 | -2.622017 | 1.857811 | 0.380891 |
| 8 | 6 | 0 | -4.577532 | -2.286595 | -0.172840 |
| 9 | 1 | 0 | -0.035264 | -1.621629 | 0.551684 |
| 10 | 6 | 0 | -5.829297 | -1.572019 | -0.316029 |
| 11 | 6 | 0 | -5.877246 | -0.219300 | -0.239912 |
| 12 | 1 | 0 | -6.739727 | -2.134731 | -0.481523 |
| 13 | 6 | 0 | -7.101333 | 0.625973 | -0.362481 |
| 14 | 1 | 0 | -7.981727 | 0.005099 | -0.530411 |
| 15 | 1 | 0 | -7.248080 | 1.215350 | 0.548652 |
| 16 | 1 | 0 | -6.995658 | 1.329507 | -1.194862 |
| 17 | 8 | 0 | -4.500120 | -3.544008 | -0.231167 |
| 18 | 8 | 0 | -1.954625 | -3.347845 | 0.175522 |
| 19 | 8 | 0 | 1.984569 | 2.381262 | -2.902852 |
| 20 | 8 | 0 | -4.766298 | 0.538277 | -0.034732 |
| 21 | 6 | 0 | 1.902902 | 1.010366 | -2.603023 |
| 22 | 1 | 0 | 2.344991 | 0.456326 | -3.437187 |
| 23 | 1 | 0 | 0.867609 | 0.652707 | -2.495138 |
| 24 | 1 | 0 | -2.869935 | -3.719964 | 0.012808 |
| 25 | 6 | 0 | 2.644433 | 0.647480 | -1.322746 |
| 26 | 8 | 0 | 3.192300 | 1.446056 | -0.595539 |
| 27 | 7 | 0 | -0.049083 | 0.978724 | 0.718345 |
| 28 | 6 | 0 | 0.114275 | 2.372046 | 0.554356 |
| 29 | 6 | 0 | 0.910234 | 3.076791 | 1.486354 |
| 30 | 6 | 0 | -0.438875 | 3.049347 | -0.545952 |
| 31 | 6 | 0 | 1.115032 | 4.445803 | 1.282386 |
| 32 | 6 | 0 | -0.228475 | 4.418955 | -0.717613 |
| 33 | 1 | 0 | -1.030548 | 2.498660 | -1.269370 |
| 34 | 6 | 0 | 0.553860 | 5.123034 | 0.198512 |
| 35 | 1 | 0 | 0.725431 | 6.187449 | 0.072479 |
| 36 | 1 | 0 | 1.602428 | 2.866311 | -2.154739 |
| 37 | 6 | 0 | 3.003563 | -1.179121 | 0.231510 |
| 38 | 8 | 0 | 3.946091 | -1.982254 | 0.178670 |
| 39 | 8 | 0 | 2.311995 | -0.785679 | 1.182293 |
| 40 | 8 | 0 | 2.596564 | -0.686683 | -1.098465 |
| 41 | 1 | 0 | 0.786131 | 0.448596 | 0.972507 |
| 42 | 19 | 0 | 3.793054 | -2.352147 | 2.965445 |
| 43 | 19 | 0 | 4.464783 | -2.628755 | -2.394723 |
| 44 | 1 | 0 | -0.669486 | 4.925758 | -1.570697 |
| 45 | 1 | 0 | 1.724543 | 4.991632 | 1.997836 |
| 46 | 6 | 0 | 1.529902 | 2.369617 | 2.666064 |
| 47 | 1 | 0 | 2.005882 | 3.085759 | 3.340243 |
| 48 | 1 | 0 | 2.290800 | 1.649294 | 2.344390 |
| 49 | 1 | 0 | 0.778595 | 1.808017 | 3.232534 |

**4**

| Center Number | Atomic Number | Atomic Type | Coordinates (Angstroms) X | Y | Z |
|---|---|---|---|---|---|
| 1 | 6 | 0 | -2.938480 | 0.238267 | -0.770211 |
| 2 | 1 | 0 | -3.640082 | -0.301258 | -1.402873 |
| 3 | 1 | 0 | -2.702332 | 1.208456 | -1.205906 |
| 4 | 6 | 0 | -1.715515 | -0.643788 | -0.522269 |
| 5 | 8 | 0 | -1.826619 | -1.864908 | -0.451410 |
| 6 | 7 | 0 | -0.552121 | 0.051777 | -0.388503 |
| 7 | 17 | 0 | -3.800293 | 0.564669 | 0.803172 |
| 8 | 1 | 0 | -0.593129 | 1.060491 | -0.463136 |
| 9 | 6 | 0 | 0.743144 | -0.440879 | -0.127726 |
| 10 | 6 | 0 | 1.765576 | 0.535393 | -0.026255 |

| | | | | | |
|---|---|---|---|---|---|
| 11 | 6 | 0 | 1.064215 | -1.791508 | 0.030364 |
| 12 | 6 | 0 | 3.079438 | 0.148614 | 0.230292 |
| 13 | 6 | 0 | 2.385917 | -2.169686 | 0.287302 |
| 14 | 1 | 0 | 0.277270 | -2.527506 | -0.049208 |
| 15 | 6 | 0 | 3.386837 | -1.207902 | 0.386862 |
| 16 | 1 | 0 | 3.862884 | 0.892302 | 0.309415 |
| 17 | 1 | 0 | 2.622883 | -3.221888 | 0.408281 |
| 18 | 1 | 0 | 4.413094 | -1.500010 | 0.586166 |
| 19 | 8 | 0 | 1.345617 | 1.825386 | -0.196851 |
| 20 | 6 | 0 | 2.306903 | 2.876607 | -0.102489 |
| 21 | 1 | 0 | 1.754320 | 3.801534 | -0.265776 |
| 22 | 1 | 0 | 2.772451 | 2.898267 | 0.888664 |
| 23 | 1 | 0 | 3.082276 | 2.773264 | -0.869158 |

**TS4**

| Center Number | Atomic Number | Atomic Type | Coordinates (Angstroms) X | Y | Z |
|---|---|---|---|---|---|
| 1 | 6 | 0 | -1.200656 | 1.033665 | 0.477276 |
| 2 | 6 | 0 | -1.793621 | -0.239746 | 0.220132 |
| 3 | 6 | 0 | -3.131620 | -0.306705 | -0.115430 |
| 4 | 6 | 0 | -3.963669 | 0.829222 | -0.225600 |
| 5 | 6 | 0 | -3.364126 | 2.102750 | 0.024166 |
| 6 | 6 | 0 | -2.028328 | 2.196650 | 0.368671 |
| 7 | 1 | 0 | -1.190628 | -1.139065 | 0.247254 |
| 8 | 6 | 0 | -5.351829 | 0.695817 | -0.581772 |
| 9 | 1 | 0 | -1.588879 | 3.169063 | 0.564724 |
| 10 | 6 | 0 | -5.806044 | -0.662669 | -0.814227 |
| 11 | 6 | 0 | -4.959068 | -1.713887 | -0.699258 |
| 12 | 1 | 0 | -6.840601 | -0.830644 | -1.087801 |
| 13 | 6 | 0 | -5.306115 | -3.150092 | -0.918837 |
| 14 | 1 | 0 | -6.357834 | -3.255087 | -1.187474 |
| 15 | 1 | 0 | -4.689704 | -3.572412 | -1.719504 |
| 16 | 1 | 0 | -5.109445 | -3.732711 | -0.012671 |
| 17 | 8 | 0 | -6.125408 | 1.691024 | -0.688294 |
| 18 | 8 | 0 | -4.127853 | 3.215145 | -0.072003 |
| 19 | 8 | 0 | 0.051851 | 1.178023 | 0.794671 |
| 20 | 8 | 0 | -3.652438 | -1.562458 | -0.358895 |
| 21 | 6 | 0 | 0.975106 | -0.280705 | 1.983638 |
| 22 | 1 | 0 | 0.004175 | -0.684071 | 2.211105 |
| 23 | 1 | 0 | 1.315733 | 0.596491 | 2.505404 |
| 24 | 1 | 0 | -5.039750 | 2.887525 | -0.321936 |
| 25 | 6 | 0 | 1.665038 | -0.796728 | 0.756203 |
| 26 | 8 | 0 | 1.214921 | -1.747431 | 0.116603 |
| 27 | 7 | 0 | 2.812260 | -0.123310 | 0.445441 |
| 28 | 17 | 0 | 1.975863 | -1.640350 | 3.465753 |
| 29 | 1 | 0 | 3.070772 | 0.654491 | 1.037817 |
| 30 | 6 | 0 | 3.693164 | -0.336008 | -0.629018 |
| 31 | 6 | 0 | 4.796559 | 0.551421 | -0.712507 |
| 32 | 6 | 0 | 3.553067 | -1.341970 | -1.589962 |
| 33 | 6 | 0 | 5.727656 | 0.420085 | -1.740326 |
| 34 | 6 | 0 | 4.493131 | -1.465631 | -2.618350 |
| 35 | 1 | 0 | 2.709389 | -2.013110 | -1.519444 |
| 36 | 6 | 0 | 5.573428 | -0.592213 | -2.695206 |
| 37 | 1 | 0 | 6.570513 | 1.097425 | -1.804170 |
| 38 | 1 | 0 | 4.370393 | -2.251538 | -3.357043 |
| 39 | 1 | 0 | 6.303732 | -0.686628 | -3.492780 |
| 40 | 8 | 0 | 4.845692 | 1.500017 | 0.272796 |
| 41 | 6 | 0 | 5.923182 | 2.434852 | 0.266477 |
| 42 | 1 | 0 | 5.760744 | 3.084135 | 1.126593 |
| 43 | 1 | 0 | 6.888587 | 1.927223 | 0.368689 |
| 44 | 1 | 0 | 5.921668 | 3.034822 | -0.650099 |

**9**

| Center Number | Atomic Number | Atomic Type | Coordinates (Angstroms) X | Y | Z |
|---|---|---|---|---|---|
| 1 | 6 | 0 | 0.790748 | 1.388369 | 0.360658 |
| 2 | 6 | 0 | 1.357472 | 0.794188 | -0.772837 |
| 3 | 6 | 0 | 2.615488 | 0.213538 | -0.630514 |
| 4 | 6 | 0 | 3.318196 | 0.199729 | 0.582986 |
| 5 | 6 | 0 | 2.707225 | 0.810765 | 1.716573 |
| 6 | 6 | 0 | 1.453980 | 1.398570 | 1.598875 |
| 7 | 1 | 0 | 0.864135 | 0.756387 | -1.734001 |
| 8 | 6 | 0 | 4.626878 | -0.424530 | 0.666644 |
| 9 | 1 | 0 | 0.986480 | 1.869259 | 2.455402 |
| 10 | 6 | 0 | 5.112464 | -1.004812 | -0.565985 |
| 11 | 6 | 0 | 4.376382 | -0.954961 | -1.704113 |
| 12 | 1 | 0 | 6.082397 | -1.486367 | -0.572481 |
| 13 | 6 | 0 | 4.766712 | -1.513759 | -3.031287 |
| 14 | 1 | 0 | 5.749268 | -1.982823 | -2.976597 |
| 15 | 1 | 0 | 4.032699 | -2.256643 | -3.360211 |
| 16 | 1 | 0 | 4.790317 | -0.719235 | -3.784272 |
| 17 | 8 | 0 | 5.281523 | -0.452533 | 1.740525 |
| 18 | 8 | 0 | 3.349239 | 0.818410 | 2.896493 |
| 19 | 8 | 0 | -0.433603 | 1.992588 | 0.367276 |
| 20 | 8 | 0 | 3.152991 | -0.362832 | -1.751812 |
| 21 | 6 | 0 | -1.150413 | 2.137510 | -0.863156 |
| 22 | 1 | 0 | -0.495777 | 2.460843 | -1.676470 |
| 23 | 1 | 0 | -1.891942 | 2.926155 | -0.676497 |
| 24 | 1 | 0 | 4.216866 | 0.347122 | 2.734450 |
| 25 | 6 | 0 | -1.891102 | 0.874436 | -1.309828 |
| 26 | 8 | 0 | -2.210512 | 0.738451 | -2.489984 |
| 27 | 7 | 0 | -2.185761 | 0.013275 | -0.298690 |
| 28 | 17 | 0 | -3.679136 | 4.780653 | -0.185241 |
| 29 | 1 | 0 | -1.857376 | 0.274843 | 0.623734 |
| 30 | 6 | 0 | -2.879952 | -1.210908 | -0.351641 |
| 31 | 6 | 0 | -3.000273 | -1.911622 | 0.874121 |
| 32 | 6 | 0 | -3.440034 | -1.754655 | -1.510435 |
| 33 | 6 | 0 | -3.671716 | -3.131945 | 0.920274 |
| 34 | 6 | 0 | -4.110890 | -2.980750 | -1.454854 |
| 35 | 1 | 0 | -3.343300 | -1.210485 | -2.439095 |
| 36 | 6 | 0 | -4.226918 | -3.665491 | -0.248909 |
| 37 | 1 | 0 | -3.764870 | -3.669225 | 1.856153 |
| 38 | 1 | 0 | -4.541232 | -3.392157 | -2.362449 |
| 39 | 1 | 0 | -4.747891 | -4.616754 | -0.203427 |
| 40 | 8 | 0 | -2.418106 | -1.294676 | 1.946977 |
| 41 | 6 | 0 | -2.500111 | -1.925214 | 3.224319 |
| 42 | 1 | 0 | -1.980823 | -1.263949 | 3.917637 |
| 43 | 1 | 0 | -3.541917 | -2.042876 | 3.541813 |
| 44 | 1 | 0 | -2.008449 | -2.904137 | 3.215069 |

**TS17c**

| Center Number | Atomic Number | Atomic Type | Coordinates (Angstroms) X | Y | Z |
|---|---|---|---|---|---|
| 1 | 6 | 0 | 0.160850 | -0.793596 | -0.702021 |
| 2 | 6 | 0 | -0.563495 | 0.405861 | -0.929281 |
| 3 | 6 | 0 | -1.862654 | 0.478574 | -0.472275 |
| 4 | 6 | 0 | -2.473366 | -0.541996 | 0.288626 |
| 5 | 6 | 0 | -1.683708 | -1.690378 | 0.597596 |
| 6 | 6 | 0 | -0.371655 | -1.786761 | 0.164146 |
| 7 | 1 | 0 | -0.135742 | 1.221294 | -1.499247 |
| 8 | 6 | 0 | -3.826487 | -0.402638 | 0.758594 |
| 9 | 1 | 0 | 0.199754 | -2.669885 | 0.429022 |
| 10 | 6 | 0 | -4.488766 | 0.833137 | 0.384281 |
| 11 | 6 | 0 | -3.848019 | 1.778007 | -0.344151 |
| 12 | 1 | 0 | -5.510763 | 0.998589 | 0.702442 |
| 13 | 6 | 0 | -4.415005 | 3.087437 | -0.783064 |
| 14 | 1 | 0 | -5.441718 | 3.198194 | -0.432961 |
| 15 | 1 | 0 | -3.812836 | 3.913499 | -0.390324 |
| 16 | 1 | 0 | -4.400188 | 3.161483 | -1.875535 |
| 17 | 8 | 0 | -4.396240 | -1.292430 | 1.452178 |
| 18 | 8 | 0 | -2.221171 | -2.673296 | 1.343977 |
| 19 | 8 | 0 | 0.467558 | -1.543626 | -2.446470 |
| 20 | 8 | 0 | -2.563620 | 1.624186 | -0.770723 |
| 21 | 6 | 0 | 1.480687 | -2.444955 | -2.258523 |
| 22 | 1 | 0 | 2.175765 | -2.518730 | -3.117288 |
| 23 | 1 | 0 | 1.183399 | -3.488056 | -2.025242 |
| 24 | 1 | 0 | -3.155387 | -2.368927 | 1.548399 |
| 25 | 6 | 0 | 2.327471 | -1.980951 | -1.084922 |
| 26 | 8 | 0 | 3.271089 | -2.418361 | -0.507826 |
| 27 | 7 | 0 | 1.687004 | -0.618476 | -0.665300 |
| 28 | 1 | 0 | 1.869055 | -0.006658 | -1.475461 |
| 29 | 6 | 0 | 2.259753 | 0.037839 | 0.504563 |
| 30 | 6 | 0 | 2.813678 | 1.312770 | 0.285377 |
| 31 | 6 | 0 | 2.273134 | -0.549395 | 1.763407 |
| 32 | 6 | 0 | 3.383382 | 2.000321 | 1.359237 |
| 33 | 6 | 0 | 2.841604 | 0.144316 | 2.833450 |
| 34 | 1 | 0 | 1.842281 | -1.531632 | 1.911315 |
| 35 | 6 | 0 | 3.391175 | 1.408975 | 2.625027 |
| 36 | 1 | 0 | 3.813805 | 2.983487 | 1.216655 |
| 37 | 1 | 0 | 2.854720 | -0.305690 | 3.819736 |
| 38 | 1 | 0 | 3.834782 | 1.950108 | 3.454646 |
| 39 | 8 | 0 | 2.734343 | 1.765818 | -0.995559 |
| 40 | 6 | 0 | 3.304309 | 3.041051 | -1.317207 |
| 41 | 1 | 0 | 3.133104 | 3.178891 | -2.383677 |
| 42 | 1 | 0 | 2.809367 | 3.839951 | -0.757108 |
| 43 | 1 | 0 | 4.378579 | 3.049376 | -1.110050 |

**18c**

| Center Number | Atomic Number | Atomic Type | Coordinates (Angstroms) X | Y | Z |
|---|---|---|---|---|---|
| 1 | 6 | 0 | -0.079635 | -0.745609 | -0.147888 |
| 2 | 6 | 0 | 0.479953 | 0.457062 | 0.296413 |
| 3 | 6 | 0 | 1.856341 | 0.613084 | 0.194913 |
| 4 | 6 | 0 | 2.693627 | -0.388284 | -0.324791 |
| 5 | 6 | 0 | 2.097140 | -1.597786 | -0.772163 |
| 6 | 6 | 0 | 0.713487 | -1.760782 | -0.698147 |
| 7 | 1 | 0 | -0.131966 | 1.240048 | 0.725243 |
| 8 | 6 | 0 | 4.131033 | -0.173525 | -0.406845 |
| 9 | 1 | 0 | 0.267005 | -2.667906 | -1.087114 |
| 10 | 6 | 0 | 4.591195 | 1.104329 | 0.086898 |
| 11 | 6 | 0 | 3.722438 | 2.022086 | 0.581856 |
| 12 | 1 | 0 | 5.650591 | 1.326880 | 0.058805 |
| 13 | 6 | 0 | 4.077814 | 3.367789 | 1.119010 |
| 14 | 1 | 0 | 5.153257 | 3.534930 | 1.057239 |
| 15 | 1 | 0 | 3.560207 | 4.149345 | 0.553367 |
| 16 | 1 | 0 | 3.761218 | 3.453565 | 2.163661 |
| 17 | 8 | 0 | 4.903792 | -1.047269 | -0.875120 |
| 18 | 8 | 0 | 2.860354 | -2.573011 | -1.290160 |
| 19 | 8 | 0 | -0.474913 | -2.224164 | 2.292273 |
| 20 | 8 | 0 | 2.383408 | 1.798690 | 0.640080 |
| 21 | 6 | 0 | -1.386524 | -2.890541 | 1.438885 |

| | | | | | |
|---|---|---|---|---|---|
| 22 | 1 | 0 | -2.160549 | -3.434104 | 1.993171 |
| 23 | 1 | 0 | -0.809115 | -3.627437 | 0.872831 |
| 24 | 1 | 0 | 3.799261 | -2.228933 | -1.254571 |
| 25 | 6 | 0 | -2.158692 | -1.980916 | 0.471206 |
| 26 | 8 | 0 | -3.347363 | -2.190014 | 0.257000 |
| 27 | 7 | 0 | -1.499888 | -0.902207 | -0.098982 |
| 28 | 1 | 0 | -0.983153 | -1.625601 | 2.857036 |
| 29 | 6 | 0 | -2.298207 | 0.106840 | -0.745250 |
| 30 | 6 | 0 | -3.078888 | 0.984421 | 0.037330 |
| 31 | 6 | 0 | -2.285510 | 0.230954 | -2.131895 |
| 32 | 6 | 0 | -3.848632 | 1.964720 | -0.599667 |
| 33 | 6 | 0 | -3.044326 | 1.217857 | -2.764989 |
| 34 | 1 | 0 | -1.674935 | -0.455588 | -2.710135 |
| 35 | 6 | 0 | -3.825910 | 2.075186 | -1.992877 |
| 36 | 1 | 0 | -4.458369 | 2.646022 | -0.019321 |
| 37 | 1 | 0 | -3.028071 | 1.307289 | -3.846019 |
| 38 | 1 | 0 | -4.426136 | 2.844098 | -2.469803 |
| 39 | 8 | 0 | -3.008255 | 0.811011 | 1.385079 |
| 40 | 6 | 0 | -3.791133 | 1.665199 | 2.220048 |
| 41 | 1 | 0 | -3.583044 | 1.351843 | 3.242970 |
| 42 | 1 | 0 | -3.505660 | 2.715556 | 2.095881 |
| 43 | 1 | 0 | -4.860924 | 1.551001 | 2.014134 |

---------------------------------------------------------------------

**TS18c**

| Center Number | Atomic Number | Atomic Type | Coordinates (Angstroms) X | Y | Z |
|---|---|---|---|---|---|
| 1 | 6 | 0 | 0.152311 | 0.598758 | -0.626227 |
| 2 | 6 | 0 | -0.485396 | 0.176183 | 0.565519 |
| 3 | 6 | 0 | -1.812553 | -0.186303 | 0.509270 |
| 4 | 6 | 0 | -2.558112 | -0.227575 | -0.695246 |
| 5 | 6 | 0 | -1.871104 | 0.132978 | -1.895852 |
| 6 | 6 | 0 | -0.539723 | 0.505736 | -1.859584 |
| 7 | 1 | 0 | 0.044171 | 0.206664 | 1.509325 |
| 8 | 6 | 0 | -3.936733 | -0.638807 | -0.693227 |
| 9 | 1 | 0 | -0.041187 | 0.788215 | -2.779636 |
| 10 | 6 | 0 | -4.483755 | -0.977554 | 0.614836 |
| 11 | 6 | 0 | -3.720993 | -0.912037 | 1.732237 |
| 12 | 1 | 0 | -5.521214 | -1.284964 | 0.677633 |
| 13 | 6 | 0 | -4.171725 | -1.231792 | 3.123876 |
| 14 | 1 | 0 | -5.221521 | -1.532384 | 3.127700 |
| 15 | 1 | 0 | -3.566479 | -2.042632 | 3.545394 |
| 16 | 1 | 0 | -4.047613 | -0.359054 | 3.775058 |
| 17 | 8 | 0 | -4.637758 | -0.709068 | -1.742304 |
| 18 | 8 | 0 | -2.532730 | 0.087083 | -3.077716 |
| 19 | 8 | 0 | 0.223800 | 2.787813 | -0.394292 |
| 20 | 8 | 0 | -2.419985 | -0.535147 | 1.708852 |
| 21 | 6 | 0 | 1.542970 | 3.056595 | -0.615274 |
| 22 | 1 | 0 | 2.038889 | 3.637906 | 0.192254 |
| 23 | 1 | 0 | 1.744542 | 3.622771 | -1.552358 |
| 24 | 1 | 0 | -3.458593 | -0.209852 | -2.866885 |
| 25 | 6 | 0 | 2.371162 | 1.773968 | -0.725668 |
| 26 | 8 | 0 | 3.591926 | 1.739244 | -0.859469 |
| 27 | 7 | 0 | 1.601699 | 0.620779 | -0.643149 |
| 28 | 6 | 0 | 2.266614 | -0.640075 | -0.550452 |
| 29 | 6 | 0 | 3.011268 | -0.959560 | 0.608815 |
| 30 | 6 | 0 | 2.192371 | -1.570401 | -1.585225 |
| 31 | 6 | 0 | 3.689776 | -2.182117 | 0.684942 |
| 32 | 6 | 0 | 2.850316 | -2.802584 | -1.502084 |
| 33 | 1 | 0 | 1.609188 | -1.315271 | -2.463805 |
| 34 | 6 | 0 | 3.605678 | -3.098821 | -0.369533 |
| 35 | 1 | 0 | 4.273324 | -2.431398 | 1.563764 |
| 36 | 1 | 0 | 2.779103 | -3.512611 | -2.320764 |
| 37 | 1 | 0 | 4.133462 | -4.046029 | -0.293038 |
| 38 | 8 | 0 | 2.992533 | -0.035423 | 1.607840 |
| 39 | 6 | 0 | 3.855801 | -0.207893 | 2.717291 |
| 40 | 1 | 0 | 3.719695 | 0.679190 | 3.337730 |
| 41 | 1 | 0 | 3.593654 | -1.101617 | 3.300891 |
| 42 | 1 | 0 | 4.904194 | -0.273995 | 2.398513 |
| 43 | 8 | 0 | -0.724244 | 3.525799 | 1.962860 |
| 44 | 1 | 0 | -1.548794 | 3.028406 | 2.010152 |
| 45 | 1 | 0 | -0.344103 | 3.266606 | 1.063465 |

---------------------------------------------------------------------

**19c**

| Center Number | Atomic Number | Atomic Type | Coordinates (Angstroms) X | Y | Z |
|---|---|---|---|---|---|
| 1 | 6 | 0 | -0.002958 | 0.680760 | -0.325448 |
| 2 | 6 | 0 | -0.568717 | -0.463980 | 0.253438 |
| 3 | 6 | 0 | -1.948797 | -0.605318 | 0.226094 |
| 4 | 6 | 0 | -2.796164 | 0.348790 | -0.363292 |
| 5 | 6 | 0 | -2.195964 | 1.496995 | -0.952577 |
| 6 | 6 | 0 | -0.813986 | 1.643633 | -0.947750 |
| 7 | 1 | 0 | 0.046692 | -1.218248 | 0.726651 |
| 8 | 6 | 0 | -4.233856 | 0.149200 | -0.369237 |
| 9 | 1 | 0 | -0.372040 | 2.511304 | -1.421962 |
| 10 | 6 | 0 | -4.689810 | -1.066073 | 0.269760 |
| 11 | 6 | 0 | -3.813856 | -1.941941 | 0.823296 |
| 12 | 1 | 0 | -5.752101 | -1.274174 | 0.303123 |
| 13 | 6 | 0 | -4.165162 | -3.221787 | 1.506150 |
| 14 | 1 | 0 | -5.244962 | -3.372307 | 1.508429 |
| 15 | 1 | 0 | -3.687185 | -4.065941 | 0.998272 |
| 16 | 1 | 0 | -3.802768 | -3.211965 | 2.539318 |
| 17 | 8 | 0 | -5.020088 | 0.980060 | -0.894909 |
| 18 | 8 | 0 | -2.971395 | 2.430726 | -1.538402 |
| 19 | 8 | 0 | 0.510571 | 2.725618 | 1.533550 |
| 20 | 8 | 0 | -2.471788 | -1.734601 | 0.809304 |
| 21 | 6 | 0 | 1.490517 | 3.077328 | 0.680150 |
| 22 | 1 | 0 | 2.395370 | 3.556636 | 1.133532 |
| 23 | 1 | 0 | 1.180960 | 3.823903 | -0.101843 |
| 24 | 1 | 0 | -3.909639 | 2.104275 | -1.432707 |
| 25 | 6 | 0 | 2.153132 | 1.946869 | -0.151588 |
| 26 | 8 | 0 | 3.313676 | 2.024365 | -0.548392 |
| 27 | 7 | 0 | 1.414894 | 0.778899 | -0.395370 |
| 28 | 6 | 0 | 2.124401 | -0.361060 | -0.905760 |
| 29 | 6 | 0 | 3.039673 | -1.054457 | -0.083696 |
| 30 | 6 | 0 | 1.910283 | -0.797180 | -2.212067 |
| 31 | 6 | 0 | 3.730435 | -2.157603 | -0.596094 |
| 32 | 6 | 0 | 2.588369 | -1.907566 | -2.720250 |
| 33 | 1 | 0 | 1.202220 | -0.252398 | -2.829065 |
| 34 | 6 | 0 | 3.501149 | -2.578640 | -1.909273 |
| 35 | 1 | 0 | 4.438267 | -2.695520 | 0.022569 |
| 36 | 1 | 0 | 2.409995 | -2.234099 | -3.739580 |
| 37 | 1 | 0 | 4.042461 | -3.439417 | -2.290135 |
| 38 | 8 | 0 | 3.178018 | -0.591182 | 1.190639 |
| 39 | 6 | 0 | 4.134271 | -1.207344 | 2.053549 |
| 40 | 1 | 0 | 4.066386 | -0.658032 | 2.992432 |
| 41 | 1 | 0 | 3.896826 | -2.262570 | 2.227700 |
| 42 | 1 | 0 | 5.147567 | -1.121712 | 1.647059 |
| 43 | 8 | 0 | 1.516877 | 0.972375 | 3.167052 |
| 44 | 1 | 0 | 1.825544 | 0.327927 | 2.516265 |
| 45 | 1 | 0 | 1.107411 | 1.687408 | 2.564367 |

---------------------------------------------------------------------

**TS19c**

| Center Number | Atomic Number | Atomic Type | Coordinates (Angstroms) X | Y | Z |
|---|---|---|---|---|---|
| 1 | 6 | 0 | -0.581859 | -0.003261 | -0.040627 |
| 2 | 6 | 0 | -1.866281 | 0.350327 | 0.429982 |
| 3 | 6 | 0 | -2.896989 | -0.571670 | 0.345213 |
| 4 | 6 | 0 | -2.732072 | -1.861611 | -0.187304 |
| 5 | 6 | 0 | -1.431950 | -2.213456 | -0.641257 |
| 6 | 6 | 0 | -0.384083 | -1.305448 | -0.562782 |
| 7 | 1 | 0 | -2.064133 | 1.319924 | 0.864881 |
| 8 | 6 | 0 | -3.844187 | -2.786380 | -0.246981 |
| 9 | 1 | 0 | 0.593101 | -1.619994 | -0.900614 |
| 10 | 6 | 0 | -5.097000 | -2.275149 | 0.267870 |
| 11 | 6 | 0 | -5.188791 | -1.018506 | 0.770367 |
| 12 | 1 | 0 | -5.971643 | -2.913624 | 0.252111 |
| 13 | 6 | 0 | -6.418867 | -0.385797 | 1.331364 |
| 14 | 1 | 0 | -7.260848 | -1.077007 | 1.286921 |
| 15 | 1 | 0 | -6.252528 | -0.091096 | 2.372738 |
| 16 | 1 | 0 | -6.669811 | 0.520249 | 0.770040 |
| 17 | 8 | 0 | -3.720369 | -3.950754 | -0.712432 |
| 18 | 8 | 0 | -1.215178 | -3.445176 | -1.145712 |
| 19 | 8 | 0 | 1.057555 | 1.904601 | -3.084924 |
| 20 | 8 | 0 | -4.126031 | -0.172405 | 0.817345 |
| 21 | 6 | 0 | 1.480729 | 0.689635 | -2.514630 |
| 22 | 1 | 0 | 2.332635 | 0.331288 | -3.102590 |
| 23 | 1 | 0 | 0.705703 | -0.082499 | -2.534453 |
| 24 | 1 | 0 | -2.098589 | -3.911507 | -1.099384 |
| 25 | 6 | 0 | 2.004970 | 0.895033 | -1.091779 |
| 26 | 8 | 0 | 2.731155 | 1.791300 | -0.733241 |
| 27 | 7 | 0 | 0.519324 | 0.863719 | 0.083953 |
| 28 | 6 | 0 | 0.284420 | 2.202218 | 0.531774 |
| 29 | 6 | 0 | -0.489593 | 3.131920 | -0.206240 |
| 30 | 6 | 0 | 0.895100 | 2.648631 | 1.708378 |
| 31 | 6 | 0 | -0.669977 | 4.435228 | 0.261996 |
| 32 | 6 | 0 | 0.732163 | 3.955660 | 2.171964 |
| 33 | 1 | 0 | 1.503095 | 1.950006 | 2.272979 |
| 34 | 6 | 0 | -0.058642 | 4.844805 | 1.449988 |
| 35 | 1 | 0 | -0.204174 | 5.862486 | 1.798777 |
| 36 | 1 | 0 | 0.268299 | 2.190855 | -2.588460 |
| 37 | 6 | 0 | 2.972121 | -0.940184 | 0.456121 |
| 38 | 8 | 0 | 3.892467 | -1.736646 | 0.677296 |
| 39 | 8 | 0 | 2.278196 | -0.406149 | 1.449375 |
| 40 | 8 | 0 | 2.620942 | -0.618422 | -0.778937 |
| 41 | 1 | 0 | 1.492583 | 0.152629 | 1.021926 |
| 42 | 19 | 0 | 3.724256 | -1.794494 | 3.448529 |
| 43 | 19 | 0 | 4.642155 | -2.258488 | -1.933137 |
| 44 | 1 | 0 | 1.215117 | 4.267962 | 3.092360 |
| 45 | 1 | 0 | -1.273046 | 5.138916 | -0.298351 |
| 46 | 8 | 0 | -1.033904 | 2.676122 | -1.383294 |
| 47 | 6 | 0 | -2.031477 | 3.475366 | -2.033608 |
| 48 | 1 | 0 | -2.388984 | 2.875884 | -2.870351 |
| 49 | 1 | 0 | -2.859214 | 3.689554 | -1.351231 |
| 50 | 1 | 0 | -1.609715 | 4.412648 | -2.410080 |

---------------------------------------------------------------------

**14**

| Center Number | Atomic Number | Atomic Type | Coordinates (Angstroms) X | Y | Z |
|---|---|---|---|---|---|
| 1 | 6 | 0 | 0.925369 | 0.054563 | -0.522743 |
| 2 | 6 | 0 | 2.278348 | 0.448129 | -0.494698 |

| | | | | | |
|---|---|---|---|---|---|
| 3 | 6 | 0 | 3.250943 | -0.536205 | -0.419385 |
| 4 | 6 | 0 | 2.954974 | -1.911102 | -0.377272 |
| 5 | 6 | 0 | 1.580676 | -2.288205 | -0.420976 |
| 6 | 6 | 0 | 0.589779 | -1.322253 | -0.492245 |
| 7 | 1 | 0 | 2.570594 | 1.488242 | -0.540714 |
| 8 | 6 | 0 | 4.013778 | -2.892106 | -0.301090 |
| 9 | 1 | 0 | -0.450189 | -1.625655 | -0.526054 |
| 10 | 6 | 0 | 5.356931 | -2.350106 | -0.274905 |
| 11 | 6 | 0 | 5.574638 | -1.012952 | -0.323077 |
| 12 | 1 | 0 | 6.197235 | -3.031075 | -0.219209 |
| 13 | 6 | 0 | 6.905535 | -0.337161 | -0.307946 |
| 14 | 1 | 0 | 7.709048 | -1.072068 | -0.252326 |
| 15 | 1 | 0 | 7.035081 | 0.266290 | -1.212431 |
| 16 | 1 | 0 | 6.978835 | 0.338443 | 0.550664 |
| 17 | 8 | 0 | 3.778866 | -4.130703 | -0.263255 |
| 18 | 8 | 0 | 1.251983 | -3.594909 | -0.389247 |
| 19 | 8 | 0 | -0.902239 | 1.194411 | 3.280891 |
| 20 | 8 | 0 | 4.558275 | -0.111948 | -0.393038 |
| 21 | 6 | 0 | -1.391665 | 0.017928 | 2.694058 |
| 22 | 1 | 0 | -1.899538 | -0.567495 | 3.469155 |
| 23 | 1 | 0 | -0.598791 | -0.621649 | 2.279398 |
| 24 | 1 | 0 | 2.122124 | -4.087125 | -0.334804 |
| 25 | 6 | 0 | -2.387524 | 0.296151 | 1.573784 |
| 26 | 8 | 0 | -2.803780 | 1.388777 | 1.263137 |
| 27 | 7 | 0 | -0.107966 | 0.965101 | -0.583863 |
| 28 | 6 | 0 | 0.030683 | 2.370608 | -0.689369 |
| 29 | 6 | 0 | 0.586058 | 3.139963 | 0.359193 |
| 30 | 6 | 0 | -0.452171 | 3.031166 | -1.823747 |
| 31 | 6 | 0 | 0.697554 | 4.526641 | 0.225466 |
| 32 | 6 | 0 | -0.372969 | 4.419390 | -1.943087 |
| 33 | 1 | 0 | -0.881478 | 2.433790 | -2.622733 |
| 34 | 6 | 0 | 0.216807 | 5.162067 | -0.922368 |
| 35 | 1 | 0 | 0.302222 | 6.240937 | -1.006374 |
| 36 | 1 | 0 | -0.385068 | 1.659146 | 2.597572 |
| 37 | 6 | 0 | -3.403120 | -0.772084 | -0.356060 |
| 38 | 8 | 0 | -4.488051 | -1.371970 | -0.399982 |
| 39 | 8 | 0 | -2.753031 | -0.187980 | -1.235769 |
| 40 | 8 | 0 | -2.751334 | -0.860784 | 0.959274 |
| 41 | 1 | 0 | -1.018162 | 0.589826 | -0.861183 |
| 42 | 19 | 0 | -4.768054 | -0.617957 | -3.099219 |
| 43 | 19 | 0 | -4.744574 | -2.848809 | 1.850192 |
| 44 | 1 | 0 | -0.751530 | 4.909296 | -2.834322 |
| 45 | 1 | 0 | 1.135996 | 5.118384 | 1.019620 |
| 46 | 8 | 0 | 0.973814 | 2.451949 | 1.483170 |
| 47 | 6 | 0 | 1.795412 | 3.126846 | 2.445609 |
| 48 | 1 | 0 | 2.058749 | 2.371854 | 3.185872 |
| 49 | 1 | 0 | 2.702110 | 3.517961 | 1.974680 |
| 50 | 1 | 0 | 1.252212 | 3.941351 | 2.935437 |

**5**

| Center Number | Atomic Number | Atomic Type | Coordinates (Angstroms) X | Y | Z |
|---|---|---|---|---|---|
| 1 | 6 | 0 | -4.025234 | 0.607741 | 0.000039 |
| 2 | 1 | 0 | -4.479029 | 1.047315 | -0.887864 |
| 3 | 1 | 0 | -4.478772 | 1.047468 | 0.887997 |
| 4 | 6 | 0 | -2.533847 | 0.955436 | -0.000167 |
| 5 | 8 | 0 | -2.256228 | 2.151977 | -0.000310 |
| 6 | 7 | 0 | -1.651140 | -0.074373 | -0.000189 |
| 7 | 17 | 0 | -4.466114 | -1.156652 | 0.000237 |
| 8 | 1 | 0 | -2.056660 | -1.002106 | -0.000012 |
| 9 | 6 | 0 | -0.238095 | -0.031427 | -0.000129 |
| 10 | 6 | 0 | 0.422783 | -1.266153 | -0.000175 |
| 11 | 6 | 0 | 1.819084 | -1.299904 | -0.000087 |
| 12 | 6 | 0 | 1.883242 | 1.106799 | 0.000093 |
| 13 | 6 | 0 | 2.568919 | -0.114990 | 0.000052 |
| 14 | 1 | 0 | 3.648179 | -0.144172 | 0.000115 |
| 15 | 1 | 0 | -0.127213 | -2.201186 | -0.000280 |
| 16 | 8 | 0 | 2.507084 | 2.320216 | 0.000199 |
| 17 | 8 | 0 | 2.371928 | -2.546671 | -0.000143 |
| 18 | 6 | 0 | 3.932537 | 2.356525 | 0.000119 |
| 19 | 1 | 0 | 4.203921 | 3.412474 | 0.000122 |
| 20 | 1 | 0 | 4.346897 | 1.876553 | 0.894113 |
| 21 | 1 | 0 | 4.346813 | 1.876562 | -0.893919 |
| 22 | 6 | 0 | 3.793303 | -2.663594 | 0.000056 |
| 23 | 1 | 0 | 4.233621 | -2.207990 | 0.894255 |
| 24 | 1 | 0 | 4.004106 | -3.733242 | 0.000003 |
| 25 | 1 | 0 | 4.233879 | -2.207834 | -0.893941 |
| 26 | 6 | 0 | 0.484024 | 1.165324 | -0.000009 |
| 27 | 1 | 0 | -0.016388 | 2.121144 | -0.000017 |

**TS5**

| Center Number | Atomic Number | Atomic Type | Coordinates (Angstroms) X | Y | Z |
|---|---|---|---|---|---|
| 1 | 6 | 0 | 1.906290 | -1.095969 | -0.748381 |
| 2 | 6 | 0 | 2.378873 | -0.220751 | 0.275885 |
| 3 | 6 | 0 | 3.635701 | 0.340152 | 0.157732 |
| 4 | 6 | 0 | 4.494823 | 0.100232 | -0.937090 |
| 5 | 6 | 0 | 4.013103 | -0.767929 | -1.965480 |
| 6 | 6 | 0 | 2.762000 | -1.348329 | -1.867476 |
| 7 | 1 | 0 | 1.743300 | 0.033932 | 1.115085 |
| 8 | 6 | 0 | 5.794453 | 0.715471 | -1.004460 |
| 9 | 1 | 0 | 2.412873 | -2.013118 | -2.650510 |
| 10 | 6 | 0 | 6.132579 | 1.577659 | 0.112622 |
| 11 | 6 | 0 | 5.263773 | 1.774474 | 1.133510 |
| 12 | 1 | 0 | 7.096917 | 2.070943 | 0.121809 |
| 13 | 6 | 0 | 5.495175 | 2.637385 | 2.330679 |
| 14 | 1 | 0 | 6.477823 | 3.107759 | 2.282007 |
| 15 | 1 | 0 | 4.728965 | 3.417448 | 2.391665 |
| 16 | 1 | 0 | 5.429630 | 2.042233 | 3.247673 |
| 17 | 8 | 0 | 6.589028 | 0.522018 | -1.969458 |
| 18 | 8 | 0 | 4.805336 | -1.021564 | -3.032115 |
| 19 | 8 | 0 | 0.736090 | -1.662500 | -0.710955 |
| 20 | 8 | 0 | 4.041946 | 1.181588 | 1.174458 |
| 21 | 6 | 0 | -0.057418 | -2.244692 | 1.134540 |
| 22 | 1 | 0 | 0.906564 | -2.088820 | 1.586089 |
| 23 | 1 | 0 | -0.239651 | -3.151424 | 0.584739 |
| 24 | 1 | 0 | 5.644011 | -0.501379 | -2.866934 |
| 25 | 6 | 0 | -0.979612 | -1.063751 | 1.057807 |
| 26 | 8 | 0 | -0.710249 | -0.001829 | 1.615432 |
| 27 | 7 | 0 | -2.116594 | -1.299413 | 0.331334 |
| 28 | 17 | 0 | -0.893802 | -3.132434 | 3.018023 |
| 29 | 1 | 0 | -2.194962 | -2.218352 | -0.081167 |
| 30 | 6 | 0 | -3.193126 | -0.433610 | 0.058699 |
| 31 | 6 | 0 | -4.232250 | -0.951666 | -0.727853 |
| 32 | 6 | 0 | -3.254847 | 0.879968 | 0.537207 |
| 33 | 6 | 0 | -5.334755 | -0.151339 | -1.032472 |
| 34 | 6 | 0 | -4.370755 | 1.663090 | 0.219248 |
| 35 | 1 | 0 | -2.460989 | 1.290017 | 1.141494 |
| 36 | 6 | 0 | -5.421478 | 1.167460 | -0.563724 |
| 37 | 1 | 0 | -6.275443 | 1.784162 | -0.800074 |
| 38 | 1 | 0 | -4.199692 | -1.968121 | -1.105999 |
| 39 | 8 | 0 | -4.346228 | 2.929705 | 0.729465 |
| 40 | 8 | 0 | -6.292759 | -0.745922 | -1.802112 |
| 41 | 6 | 0 | -5.445826 | 3.795602 | 0.460361 |
| 42 | 1 | 0 | -5.214791 | 4.736274 | 0.961051 |
| 43 | 1 | 0 | -5.561541 | 3.977119 | -0.614425 |
| 44 | 1 | 0 | -6.382746 | 3.392712 | 0.862194 |
| 45 | 6 | 0 | -7.453625 | 0.006095 | -2.147225 |
| 46 | 1 | 0 | -7.197320 | 0.895243 | -2.734621 |
| 47 | 1 | 0 | -8.070917 | -0.658726 | -2.752033 |
| 48 | 1 | 0 | -8.015003 | 0.310089 | -1.256285 |

**10**

| Center Number | Atomic Number | Atomic Type | Coordinates (Angstroms) X | Y | Z |
|---|---|---|---|---|---|
| 1 | 6 | 0 | 1.867355 | 1.375475 | 0.379730 |
| 2 | 6 | 0 | 2.124754 | 0.566717 | -0.733004 |
| 3 | 6 | 0 | 3.131657 | -0.388170 | -0.611501 |
| 4 | 6 | 0 | 3.877824 | -0.566523 | 0.562351 |
| 5 | 6 | 0 | 3.583525 | 0.272534 | 1.676295 |
| 6 | 6 | 0 | 2.585553 | 1.234141 | 1.578402 |
| 7 | 1 | 0 | 1.576223 | 0.640995 | -1.661564 |
| 8 | 6 | 0 | 4.913999 | -1.582458 | 0.626843 |
| 9 | 1 | 0 | 2.361327 | 1.879344 | 2.419360 |
| 10 | 6 | 0 | 5.092630 | -2.357331 | -0.581150 |
| 11 | 6 | 0 | 4.331515 | -2.130380 | -1.680626 |
| 12 | 1 | 0 | 5.849059 | -3.131993 | -0.600666 |
| 13 | 6 | 0 | 4.419992 | -2.860502 | -2.978666 |
| 14 | 1 | 0 | 5.195970 | -3.625046 | -2.936364 |
| 15 | 1 | 0 | 3.461369 | -3.335121 | -3.212300 |
| 16 | 1 | 0 | 4.648170 | -2.162709 | -3.790964 |
| 17 | 8 | 0 | 5.599572 | -1.766009 | 1.665445 |
| 18 | 8 | 0 | 4.276569 | 0.132260 | 2.818207 |
| 19 | 8 | 0 | 0.908832 | 2.348486 | 0.403883 |
| 20 | 8 | 0 | 3.368039 | -1.171062 | -1.711282 |
| 21 | 6 | 0 | 0.198798 | 2.667634 | -0.797362 |
| 22 | 1 | 0 | 0.864554 | 2.705100 | -1.663186 |
| 23 | 1 | 0 | -0.214797 | 3.671950 | -0.629373 |
| 24 | 1 | 0 | 4.929101 | -0.607134 | 2.648775 |
| 25 | 6 | 0 | -0.957770 | 1.715791 | -1.114475 |
| 26 | 8 | 0 | -1.401732 | 1.655879 | -2.258594 |
| 27 | 7 | 0 | -1.437592 | 1.041447 | -0.030864 |
| 28 | 17 | 0 | -1.065450 | 6.038338 | -0.015443 |
| 29 | 1 | 0 | -0.949698 | 1.229797 | 0.836421 |
| 30 | 6 | 0 | -2.506799 | 0.124970 | 0.052608 |
| 31 | 6 | 0 | -2.765417 | -0.426304 | 1.314332 |
| 32 | 6 | 0 | -3.284267 | -0.231930 | -1.053794 |
| 33 | 6 | 0 | -3.810384 | -1.340060 | 1.466700 |
| 34 | 6 | 0 | -4.325842 | -1.150726 | -0.877057 |
| 35 | 1 | 0 | -3.093120 | 0.189025 | -2.028781 |
| 36 | 6 | 0 | -4.605188 | -1.715724 | 0.374283 |
| 37 | 1 | 0 | -5.412861 | -2.421779 | 0.495376 |
| 38 | 1 | 0 | -2.169586 | -0.157359 | 2.180060 |
| 39 | 8 | 0 | -5.032257 | -1.442018 | -2.008388 |
| 40 | 8 | 0 | -3.985151 | -1.821051 | 2.731630 |
| 41 | 6 | 0 | -6.114526 | -2.365458 | -1.919453 |
| 42 | 1 | 0 | -6.524515 | -2.442667 | -2.926816 |
| 43 | 1 | 0 | -5.773876 | -3.354170 | -1.590969 |
| 44 | 1 | 0 | -6.894466 | -2.006326 | -1.238181 |
| 45 | 6 | 0 | -5.033313 | -2.758696 | 2.966313 |
| 46 | 1 | 0 | -4.892205 | -3.674108 | 2.380362 |
| 47 | 1 | 0 | -4.985100 | -3.000185 | 4.028422 |
| 48 | 1 | 0 | -6.015306 | -2.329345 | 2.737150 |

**TS17d**

| Center Number | Atomic Number | Atomic Type | Coordinates (Angstroms) X | Y | Z |
|---|---|---|---|---|---|
| 1 | 6 | 0 | 0.687526 | 2.134905 | -0.458617 |
| 2 | 6 | 0 | 1.577521 | 1.251533 | -1.175885 |
| 3 | 6 | 0 | 2.318942 | 0.319342 | -0.496123 |
| 4 | 6 | 0 | 2.325809 | 0.197897 | 0.917961 |
| 5 | 6 | 0 | 1.515008 | 1.137918 | 1.643120 |
| 6 | 6 | 0 | 0.770097 | 2.088729 | 0.993517 |
| 7 | 1 | 0 | 1.628966 | 1.317321 | -2.256146 |
| 8 | 6 | 0 | 3.134113 | -0.775692 | 1.572710 |
| 9 | 1 | 0 | 0.189500 | 2.794294 | 1.579299 |
| 10 | 6 | 0 | 3.923120 | -1.627453 | 0.692141 |
| 11 | 6 | 0 | 3.886421 | -1.469983 | -0.650438 |
| 12 | 1 | 0 | 4.555020 | -2.391651 | 1.128955 |
| 13 | 6 | 0 | 4.652832 | -2.270287 | -1.653317 |
| 14 | 1 | 0 | 5.272125 | -3.017755 | -1.155726 |
| 15 | 1 | 0 | 3.969193 | -2.777322 | -2.342889 |
| 16 | 1 | 0 | 5.296557 | -1.618038 | -2.253374 |
| 17 | 8 | 0 | 3.182531 | -0.902203 | 2.837894 |
| 18 | 8 | 0 | 1.532799 | 1.082240 | 3.000192 |
| 19 | 8 | 0 | 0.641080 | 3.426521 | -1.051511 |
| 20 | 8 | 0 | 3.112822 | -0.524967 | -1.254002 |
| 21 | 6 | 0 | -0.620432 | 4.055505 | -0.896528 |
| 22 | 1 | 0 | -0.766381 | 4.748208 | -1.730792 |
| 23 | 1 | 0 | -0.688466 | 4.632430 | 0.038845 |
| 24 | 1 | 0 | 2.155453 | 0.327368 | 3.219025 |
| 25 | 6 | 0 | -1.694802 | 2.969086 | -0.886452 |
| 26 | 8 | 0 | -2.906087 | 3.241708 | -0.880444 |
| 27 | 7 | 0 | -1.072080 | 1.783254 | -0.882569 |
| 28 | 6 | 0 | -1.692680 | 0.558390 | -0.608335 |
| 29 | 6 | 0 | -1.251452 | -0.586525 | -1.289347 |
| 30 | 6 | 0 | -2.700357 | 0.439884 | 0.363874 |
| 31 | 6 | 0 | -1.811879 | -1.832592 | -0.995420 |
| 32 | 6 | 0 | -3.260622 | -0.810021 | 0.634615 |
| 33 | 1 | 0 | -3.063537 | 1.312328 | 0.891033 |
| 34 | 6 | 0 | -2.824160 | -1.963774 | -0.034340 |
| 35 | 1 | 0 | -3.260289 | -2.927517 | 0.182675 |
| 36 | 1 | 0 | -0.477053 | -0.512447 | -2.041996 |
| 37 | 8 | 0 | -1.309937 | -2.885880 | -1.712419 |
| 38 | 8 | 0 | -4.243371 | -0.819177 | 1.589167 |
| 39 | 6 | 0 | -1.835648 | -4.186384 | -1.468958 |
| 40 | 1 | 0 | -1.295180 | -4.858527 | -2.136566 |
| 41 | 1 | 0 | -1.671983 | -4.500819 | -0.431378 |
| 42 | 1 | 0 | -2.907233 | -4.238668 | -1.695711 |
| 43 | 6 | 0 | -4.844406 | -2.062620 | 1.934717 |
| 44 | 1 | 0 | -4.107737 | -2.770960 | 2.332102 |
| 45 | 1 | 0 | -5.578471 | -1.836469 | 2.709069 |
| 46 | 1 | 0 | -5.354339 | -2.517637 | 1.077170 |

**18d**

| Center Number | Atomic Number | Atomic Type | Coordinates (Angstroms) X | Y | Z |
|---|---|---|---|---|---|
| 1 | 6 | 0 | 0.49446800 | 1.99964300 | -0.33292800 |
| 2 | 6 | 0 | 1.23097500 | 0.94928700 | -1.13233200 |
| 3 | 6 | 0 | 2.11526000 | 0.09648400 | -0.52399900 |
| 4 | 6 | 0 | 2.35329500 | 0.13438800 | 0.86940000 |
| 5 | 6 | 0 | 1.66660300 | 1.09161400 | 1.70995300 |
| 6 | 6 | 0 | 0.78314700 | 1.96674500 | 1.14445000 |
| 7 | 1 | 0 | 1.12761800 | 0.96967400 | -2.21076700 |
| 8 | 6 | 0 | 3.31940100 | -0.82149700 | 1.45361300 |
| 9 | 1 | 0 | 0.25477600 | 2.68505100 | 1.76081000 |
| 10 | 6 | 0 | 3.93987000 | -1.71178000 | 0.51383100 |
| 11 | 6 | 0 | 3.65534600 | -1.66918900 | -0.82047300 |
| 12 | 1 | 0 | 4.65646500 | -2.43420800 | 0.88301700 |
| 13 | 6 | 0 | 4.24581300 | -2.54192900 | -1.86908300 |
| 14 | 1 | 0 | 4.96113000 | -3.23821300 | -1.43315800 |
| 15 | 1 | 0 | 3.45361700 | -3.10329400 | -2.37524600 |
| 16 | 1 | 0 | 4.74835700 | -1.92862400 | -2.62406600 |
| 17 | 8 | 0 | 3.54993000 | -0.82494800 | 2.68026900 |
| 18 | 8 | 0 | 1.89934500 | 1.11377800 | 3.02694800 |
| 19 | 8 | 0 | 0.85647600 | 3.28412600 | -0.86341000 |
| 20 | 8 | 0 | 2.76700800 | -0.78801600 | -1.33515600 |
| 21 | 6 | 0 | -0.31384800 | 4.01432300 | -1.25311800 |
| 22 | 1 | 0 | -0.20574800 | 4.35280400 | -2.28710700 |
| 23 | 1 | 0 | -0.46234300 | 4.88024300 | -0.59896100 |
| 24 | 1 | 0 | 2.56769400 | 0.40056700 | 3.21877000 |
| 25 | 6 | 0 | -1.47757900 | 3.04632500 | -1.12778400 |
| 26 | 8 | 0 | -2.63916700 | 3.22846500 | -1.43517800 |
| 27 | 7 | 0 | -0.94977400 | 1.89435800 | -0.58604900 |
| 28 | 6 | 0 | -1.65094100 | 0.66617100 | -0.36831800 |
| 29 | 6 | 0 | -1.37848800 | -0.41382500 | -1.21888100 |
| 30 | 6 | 0 | -2.54485800 | 0.55213900 | 0.68097600 |
| 31 | 6 | 0 | -1.98955900 | -1.66027200 | -0.95663400 |
| 32 | 6 | 0 | -3.18254200 | -0.69076800 | 0.90274300 |
| 33 | 1 | 0 | -2.74533600 | 1.38042600 | 1.34883500 |
| 34 | 6 | 0 | -2.89500600 | -1.80329900 | 0.10136100 |
| 35 | 1 | 0 | -3.37324100 | -2.75285600 | 0.28960200 |
| 36 | 1 | 0 | -0.81273100 | -0.29562700 | -2.13292500 |
| 37 | 8 | 0 | -1.65634500 | -2.64740800 | -1.81049100 |
| 38 | 8 | 0 | -4.03864000 | -0.70698200 | 1.94064200 |
| 39 | 6 | 0 | -2.26020000 | -3.93809100 | -1.65524600 |
| 40 | 1 | 0 | -1.84374300 | -4.55337800 | -2.45136500 |
| 41 | 1 | 0 | -2.00882900 | -4.37385300 | -0.68321600 |
| 42 | 1 | 0 | -3.34723800 | -3.87705600 | -1.76632300 |
| 43 | 6 | 0 | -4.70740600 | -1.92873700 | 2.28069500 |
| 44 | 1 | 0 | -3.98647600 | -2.70684800 | 2.54965900 |
| 45 | 1 | 0 | -5.32978300 | -1.69099500 | 3.14196500 |
| 46 | 1 | 0 | -5.33713900 | -2.27283900 | 1.45447100 |

**TS18d**

| Center Number | Atomic Number | Atomic Type | Coordinates (Angstroms) X | Y | Z |
|---|---|---|---|---|---|
| 1 | 6 | 0 | 0.456487 | 1.188545 | 0.263666 |
| 2 | 6 | 0 | 1.328835 | 0.674295 | -0.757235 |
| 3 | 6 | 0 | 2.372968 | -0.147149 | -0.401054 |
| 4 | 6 | 0 | 2.573994 | -0.620214 | 0.918617 |
| 5 | 6 | 0 | 1.618663 | -0.209190 | 1.904957 |
| 6 | 6 | 0 | 0.569215 | 0.620080 | 1.576735 |
| 7 | 1 | 0 | 1.201161 | 0.983615 | -1.788179 |
| 8 | 6 | 0 | 3.658275 | -1.502592 | 1.227975 |
| 9 | 1 | 0 | -0.149480 | 0.903212 | 2.337689 |
| 10 | 6 | 0 | 4.519935 | -1.848514 | 0.108046 |
| 11 | 6 | 0 | 4.282015 | -1.367725 | -1.134330 |
| 12 | 1 | 0 | 5.361441 | -2.509132 | 0.278771 |
| 13 | 6 | 0 | 5.086772 | -1.654896 | -2.359818 |
| 14 | 1 | 0 | 5.918115 | -2.321205 | -2.126537 |
| 15 | 1 | 0 | 4.460656 | -2.122285 | -3.127382 |
| 16 | 1 | 0 | 5.483471 | -0.725815 | -2.782963 |
| 17 | 8 | 0 | 3.859991 | -1.961953 | 2.392882 |
| 18 | 8 | 0 | 1.753998 | -0.676387 | 3.169441 |
| 19 | 8 | 0 | 0.970430 | 2.995265 | 0.482719 |
| 20 | 8 | 0 | 3.239712 | -0.535078 | -1.407108 |
| 21 | 6 | 0 | 0.070240 | 3.730160 | -0.264338 |
| 22 | 1 | 0 | 0.472838 | 4.093138 | -1.230676 |
| 23 | 1 | 0 | -0.313734 | 4.623506 | 0.262275 |
| 24 | 1 | 0 | 2.569717 | -1.257373 | 3.146933 |
| 25 | 6 | 0 | -1.140522 | 2.857488 | -0.569206 |
| 26 | 8 | 0 | -2.177729 | 3.260864 | -1.088206 |
| 27 | 7 | 0 | -0.900835 | 1.564962 | -0.160759 |
| 28 | 6 | 0 | -1.919484 | 0.562705 | -0.201712 |
| 29 | 6 | 0 | -1.635309 | -0.716241 | -0.688836 |
| 30 | 6 | 0 | -3.199146 | 0.868278 | 0.273028 |
| 31 | 6 | 0 | -2.641101 | -1.688206 | -0.698142 |
| 32 | 6 | 0 | -4.196774 | -0.109929 | 0.240884 |

| | | | | | |
|---|---|---|---|---|---|
| 33 | 1 | 0 | -3.435702 | 1.851959 | 0.654510 |
| 34 | 6 | 0 | -3.933254 | -1.400353 | -0.239797 |
| 35 | 1 | 0 | -4.706708 | -2.153881 | -0.254612 |
| 36 | 1 | 0 | -0.649249 | -0.969369 | -1.055059 |
| 37 | 8 | 0 | -2.266583 | -2.907930 | -1.188074 |
| 38 | 8 | 0 | -5.415579 | 0.284549 | 0.717459 |
| 39 | 6 | 0 | -3.239329 | -3.947862 | -1.239817 |
| 40 | 1 | 0 | -2.728633 | -4.814668 | -1.660360 |
| 41 | 1 | 0 | -3.613805 | -4.198785 | -0.240555 |
| 42 | 1 | 0 | -4.083646 | -3.677732 | -1.884760 |
| 43 | 6 | 0 | -6.478362 | -0.664314 | 0.736993 |
| 44 | 1 | 0 | -6.237361 | -1.526120 | 1.370206 |
| 45 | 1 | 0 | -7.339012 | -0.140883 | 1.154546 |
| 46 | 1 | 0 | -6.723817 | -1.014884 | -0.272284 |
| 47 | 8 | 0 | 3.289773 | 3.359514 | -0.856874 |
| 48 | 1 | 0 | 3.375736 | 2.517190 | -1.320585 |
| 49 | 1 | 0 | 2.487054 | 3.207812 | -0.293278 |

**19d**

| Center | Atomic | Atomic | Coordinates (Angstroms) | | |
|---|---|---|---|---|---|
| Number | Number | Type | X | Y | Z |
| 1 | 6 | 0 | 0.394350 | 0.580921 | 0.189401 |
| 2 | 6 | 0 | 1.453925 | 0.548521 | -0.725969 |
| 3 | 6 | 0 | 2.572780 | -0.206004 | -0.407898 |
| 4 | 6 | 0 | 2.657284 | -0.982479 | 0.761261 |
| 5 | 6 | 0 | 1.546281 | -0.970610 | 1.650059 |
| 6 | 6 | 0 | 0.427390 | -0.198077 | 1.356732 |
| 7 | 1 | 0 | 1.418637 | 1.113970 | -1.648502 |
| 8 | 6 | 0 | 3.846514 | -1.768656 | 1.035541 |
| 9 | 1 | 0 | -0.408488 | -0.187415 | 2.046244 |
| 10 | 6 | 0 | 4.889300 | -1.679437 | 0.036026 |
| 11 | 6 | 0 | 4.741533 | -0.907592 | -1.069443 |
| 12 | 1 | 0 | 5.803123 | -2.242356 | 0.180499 |
| 13 | 6 | 0 | 5.743468 | -0.735757 | -2.161956 |
| 14 | 1 | 0 | 6.634529 | -1.331671 | -1.963272 |
| 15 | 1 | 0 | 5.314164 | -1.041837 | -3.121564 |
| 16 | 1 | 0 | 6.029858 | 0.317363 | -2.251015 |
| 17 | 8 | 0 | 3.953241 | -2.475575 | 2.071176 |
| 18 | 8 | 0 | 1.583633 | -1.703932 | 2.778697 |
| 19 | 8 | 0 | 1.156210 | 3.104837 | 0.868998 |
| 20 | 8 | 0 | 3.617229 | -0.179853 | -1.302800 |
| 21 | 6 | 0 | 0.407031 | 3.507592 | -0.178524 |
| 22 | 1 | 0 | 0.959761 | 3.590620 | -1.153461 |
| 23 | 1 | 0 | -0.069112 | 4.515337 | -0.076879 |
| 24 | 1 | 0 | 2.473866 | -2.157704 | 2.774699 |
| 25 | 6 | 0 | -0.824774 | 2.635652 | -0.524810 |
| 26 | 8 | 0 | -1.816691 | 3.104259 | -1.078983 |
| 27 | 7 | 0 | -0.802340 | 1.291470 | -0.126847 |
| 28 | 6 | 0 | -2.014397 | 0.522177 | -0.189250 |
| 29 | 6 | 0 | -2.042405 | -0.655677 | -0.938366 |
| 30 | 6 | 0 | -3.142564 | 0.945557 | 0.515292 |
| 31 | 6 | 0 | -3.215299 | -1.417662 | -0.976694 |
| 32 | 6 | 0 | -4.313440 | 0.183404 | 0.454222 |
| 33 | 1 | 0 | -3.127771 | 1.855747 | 1.101215 |
| 34 | 6 | 0 | -4.363581 | -1.006850 | -0.286239 |
| 35 | 1 | 0 | -5.268139 | -1.595493 | -0.321755 |
| 36 | 1 | 0 | -1.171273 | -0.991263 | -1.488355 |
| 37 | 8 | 0 | -3.149507 | -2.555592 | -1.728360 |
| 38 | 8 | 0 | -5.369482 | 0.674328 | 1.167220 |
| 39 | 6 | 0 | -4.311277 | -3.375884 | -1.826355 |
| 40 | 1 | 0 | -4.032666 | -4.213044 | -2.466948 |
| 41 | 1 | 0 | -4.617835 | -3.757515 | -0.845642 |
| 42 | 1 | 0 | -5.148388 | -2.834593 | -2.282124 |
| 43 | 6 | 0 | -6.590170 | -0.061804 | 1.172782 |
| 44 | 1 | 0 | -6.454092 | -1.063407 | 1.596866 |
| 45 | 1 | 0 | -7.277625 | 0.504729 | 1.801487 |
| 46 | 1 | 0 | -7.011682 | -0.149709 | 0.164748 |
| 47 | 8 | 0 | 3.551598 | 3.194089 | -0.046940 |
| 48 | 1 | 0 | 3.580450 | 2.453085 | -0.663331 |
| 49 | 1 | 0 | 2.607518 | 3.130359 | 0.358076 |

**TS19d**

| Center | Atomic | Atomic | Coordinates (Angstroms) | | |
|---|---|---|---|---|---|
| Number | Number | Type | X | Y | Z |
| 1 | 6 | 0 | -0.919761 | 0.559829 | 0.069921 |
| 2 | 6 | 0 | -1.948101 | -0.392512 | 0.063635 |
| 3 | 6 | 0 | -3.259447 | 0.048611 | -0.078358 |
| 4 | 6 | 0 | -3.597464 | 1.402549 | -0.210583 |
| 5 | 6 | 0 | -2.539634 | 2.351005 | -0.200926 |
| 6 | 6 | 0 | -1.219633 | 1.928741 | -0.070375 |
| 7 | 1 | 0 | -1.751187 | -1.450084 | 0.163296 |
| 8 | 6 | 0 | -4.987199 | 1.805841 | -0.355652 |
| 9 | 1 | 0 | -0.436435 | 2.673353 | -0.061802 |
| 10 | 6 | 0 | -5.943637 | 0.722120 | -0.346997 |
| 11 | 6 | 0 | -5.543640 | -0.568051 | -0.213629 |
| 12 | 1 | 0 | -6.997282 | 0.950185 | -0.449315 |
| 13 | 6 | 0 | -6.433961 | -1.765065 | -0.190160 |
| 14 | 1 | 0 | -7.477761 | -1.469361 | -0.297166 |
| 15 | 1 | 0 | -6.169740 | -2.449089 | -1.003198 |
| 16 | 1 | 0 | -6.310299 | -2.309994 | 0.751403 |
| 17 | 8 | 0 | -5.315915 | 3.013042 | -0.478074 |
| 18 | 8 | 0 | -2.812405 | 3.660905 | -0.328697 |
| 19 | 8 | 0 | 0.578778 | -0.020873 | 3.539013 |
| 20 | 8 | 0 | -4.236138 | -0.914796 | -0.082765 |
| 21 | 6 | 0 | 0.521792 | 1.113773 | 2.691134 |
| 22 | 1 | 0 | 0.951550 | 2.000832 | 3.178072 |
| 23 | 1 | 0 | -0.522498 | 1.325833 | 2.461380 |
| 24 | 1 | 0 | -3.806755 | 3.722572 | -0.413208 |
| 25 | 6 | 0 | 1.382358 | 0.840230 | 1.439957 |
| 26 | 8 | 0 | 2.438091 | 0.176901 | 1.625381 |
| 27 | 7 | 0 | 0.463636 | 0.172611 | 0.181468 |
| 28 | 6 | 0 | 0.737428 | -1.245713 | -0.010465 |
| 29 | 6 | 0 | 0.604991 | -2.160560 | 1.033492 |
| 30 | 6 | 0 | 1.108530 | -1.663810 | -1.287851 |
| 31 | 6 | 0 | 0.860250 | -3.514441 | 0.788063 |
| 32 | 6 | 0 | 1.355711 | -3.022887 | -1.521590 |
| 33 | 1 | 0 | 1.201074 | -0.963032 | -2.109288 |
| 34 | 6 | 0 | 1.235620 | -3.960206 | -0.488028 |
| 35 | 1 | 0 | 1.429570 | -5.006866 | -0.669884 |
| 36 | 1 | 0 | 0.328363 | -1.833732 | 2.028049 |
| 37 | 1 | 0 | 1.480429 | -0.365433 | 3.400611 |
| 38 | 6 | 0 | 2.333699 | 2.347168 | -0.287462 |
| 39 | 8 | 0 | 3.253331 | 3.160020 | -0.412537 |
| 40 | 8 | 0 | 1.924318 | 1.556778 | -1.241295 |
| 41 | 8 | 0 | 1.678793 | 2.255704 | 0.897151 |
| 42 | 1 | 0 | 1.162470 | 0.844154 | -0.727591 |
| 43 | 19 | 0 | 3.751213 | 2.601193 | -3.117904 |
| 44 | 19 | 0 | 4.409779 | 2.064862 | 2.068529 |
| 45 | 8 | 0 | 1.711898 | -3.341660 | -2.798724 |
| 46 | 8 | 0 | 0.718186 | -4.340071 | 1.864360 |
| 47 | 6 | 0 | 0.949158 | -5.736675 | 1.690178 |
| 48 | 1 | 0 | 0.777428 | -6.191229 | 2.666050 |
| 49 | 1 | 0 | 0.254793 | -6.170412 | 0.961573 |
| 50 | 1 | 0 | 1.979273 | -5.936683 | 1.373866 |
| 51 | 6 | 0 | 1.966662 | -4.709447 | -3.114904 |
| 52 | 1 | 0 | 1.081017 | -5.331552 | -2.942946 |
| 53 | 1 | 0 | 2.220354 | -4.728142 | -4.174893 |
| 54 | 1 | 0 | 2.807504 | -5.105264 | -2.534067 |

**15**

| Center | Atomic | Atomic | Coordinates (Angstroms) | | |
|---|---|---|---|---|---|
| Number | Number | Type | X | Y | Z |
| 1 | 6 | 0 | 1.251261 | -0.197826 | -0.296933 |
| 2 | 6 | 0 | 2.537716 | 0.374563 | -0.376950 |
| 3 | 6 | 0 | 3.639555 | -0.466734 | -0.331884 |
| 4 | 6 | 0 | 3.539650 | -1.864218 | -0.218479 |
| 5 | 6 | 0 | 2.230564 | -2.424954 | -0.155704 |
| 6 | 6 | 0 | 1.114449 | -1.604760 | -0.191554 |
| 7 | 1 | 0 | 2.686812 | 1.437167 | -0.502551 |
| 8 | 6 | 0 | 4.726929 | -2.689262 | -0.179402 |
| 9 | 1 | 0 | 0.127109 | -2.047209 | -0.128759 |
| 10 | 6 | 0 | 5.980691 | -1.969823 | -0.267617 |
| 11 | 6 | 0 | 6.007706 | -0.619088 | -0.382626 |
| 12 | 1 | 0 | 6.909101 | -2.527008 | -0.244744 |
| 13 | 6 | 0 | 7.230294 | 0.230744 | -0.486564 |
| 14 | 1 | 0 | 8.129711 | -0.384713 | -0.455242 |
| 15 | 1 | 0 | 7.218706 | 0.799782 | -1.422029 |
| 16 | 1 | 0 | 7.261697 | 0.952497 | 0.336264 |
| 17 | 8 | 0 | 4.666329 | -3.944698 | -0.079946 |
| 18 | 8 | 0 | 2.086950 | -3.760416 | -0.050945 |
| 19 | 8 | 0 | -2.255895 | -0.013776 | 3.990674 |
| 20 | 8 | 0 | 4.873897 | 0.131451 | -0.415426 |
| 21 | 6 | 0 | -1.911764 | -1.044197 | 3.095034 |
| 22 | 1 | 0 | -2.200512 | -2.014255 | 3.514601 |
| 23 | 1 | 0 | -0.827644 | -1.085474 | 2.900160 |
| 24 | 1 | 0 | 3.017970 | -4.128344 | -0.037146 |
| 25 | 6 | 0 | -2.600537 | -0.848678 | 1.755642 |
| 26 | 8 | 0 | -3.187817 | 0.176778 | 1.468945 |
| 27 | 7 | 0 | 0.086343 | 0.539556 | -0.357773 |
| 28 | 6 | 0 | -0.102263 | 1.926381 | -0.256060 |
| 29 | 6 | 0 | 0.711328 | 2.750135 | 0.534195 |
| 30 | 6 | 0 | -1.204963 | 2.475558 | -0.928536 |
| 31 | 6 | 0 | 0.429492 | 4.118101 | 0.620067 |
| 32 | 6 | 0 | -1.480612 | 3.839698 | -0.815299 |
| 33 | 1 | 0 | -1.851046 | 1.850042 | -1.534089 |
| 34 | 6 | 0 | -0.662877 | 4.684556 | -0.050258 |
| 35 | 1 | 0 | -0.872387 | 5.740827 | 0.024457 |
| 36 | 1 | 0 | 1.533105 | 2.353567 | 1.115085 |
| 37 | 1 | 0 | -2.569316 | 0.721475 | 3.437787 |
| 38 | 6 | 0 | -2.934754 | -1.785994 | -0.457409 |
| 39 | 8 | 0 | -3.863987 | -2.558105 | -0.721756 |
| 40 | 8 | 0 | -2.284540 | -0.996949 | -1.152924 |
| 41 | 8 | 0 | -2.471247 | -1.915744 | 0.952184 |
| 42 | 1 | 0 | -0.752545 | 0.007420 | -0.596408 |
| 43 | 19 | 0 | -3.823192 | -1.617017 | -3.397156 |
| 44 | 19 | 0 | -4.191549 | -4.365313 | 1.283609 |
| 45 | 8 | 0 | -2.577433 | 4.272836 | -1.504452 |
| 46 | 8 | 0 | 1.282775 | 4.833751 | 1.410392 |
| 47 | 6 | 0 | 1.046377 | 6.228949 | 1.582613 |
| 48 | 1 | 0 | 1.832258 | 6.584810 | 2.249423 |
| 49 | 1 | 0 | 1.109192 | 6.767875 | 0.630265 |
| 50 | 1 | 0 | 0.069159 | 6.417664 | 2.041755 |

| | | | | | |
|---|---|---|---|---|---|
| 51 | 6 | 0 | -2.918873 | 5.655897 | -1.444502 |
| 52 | 1 | 0 | -2.119900 | 6.285472 | -1.852665 |
| 53 | 1 | 0 | -3.814016 | 5.769354 | -2.056547 |
| 54 | 1 | 0 | -3.140225 | 5.971111 | -0.418409 |

**6**

| Center Number | Atomic Number | Atomic Type | Coordinates (Angstroms) X | Y | Z |
|---|---|---|---|---|---|
| 1 | 6 | 0 | -3.211930 | 0.799379 | -0.131296 |
| 2 | 1 | 0 | -3.673617 | 0.921518 | -1.110869 |
| 3 | 1 | 0 | -3.774747 | 1.375707 | 0.602658 |
| 4 | 6 | 0 | -1.792303 | 1.366998 | -0.198124 |
| 5 | 8 | 0 | -1.689926 | 2.548385 | -0.509627 |
| 6 | 7 | 0 | -0.765187 | 0.519938 | 0.088595 |
| 7 | 17 | 0 | -3.382326 | -0.950468 | 0.324244 |
| 8 | 1 | 0 | -1.030883 | -0.428069 | 0.324521 |
| 9 | 6 | 0 | 0.616773 | 0.811754 | 0.093855 |
| 10 | 6 | 0 | 1.549368 | -0.250587 | 0.040363 |
| 11 | 6 | 0 | 1.094416 | 2.126837 | 0.180775 |
| 12 | 6 | 0 | 2.918967 | 0.018960 | 0.080735 |
| 13 | 6 | 0 | 2.465564 | 2.375508 | 0.213754 |
| 14 | 1 | 0 | 0.386469 | 2.941470 | 0.216420 |
| 15 | 6 | 0 | 3.384686 | 1.328519 | 0.168879 |
| 16 | 1 | 0 | 3.617904 | -0.807515 | 0.038778 |
| 17 | 1 | 0 | 2.811964 | 3.401972 | 0.283059 |
| 18 | 1 | 0 | 4.450847 | 1.525080 | 0.199299 |
| 19 | 6 | 0 | 1.093551 | -1.674721 | -0.119870 |
| 20 | 9 | 0 | 2.105066 | -2.557376 | -0.045150 |
| 21 | 9 | 0 | 0.469467 | -1.878099 | -1.305550 |
| 22 | 9 | 0 | 0.189108 | -2.032829 | 0.841410 |

**TS6**

| Center Number | Atomic Number | Atomic Type | Coordinates (Angstroms) X | Y | Z |
|---|---|---|---|---|---|
| 1 | 6 | 0 | 1.547606 | -1.055658 | 0.155253 |
| 2 | 6 | 0 | 2.186517 | 0.215868 | 0.261278 |
| 3 | 6 | 0 | 3.538383 | 0.316013 | -0.005897 |
| 4 | 6 | 0 | 4.338670 | -0.783247 | -0.386157 |
| 5 | 6 | 0 | 3.692398 | -2.053267 | -0.497941 |
| 6 | 6 | 0 | 2.341691 | -2.181243 | -0.230005 |
| 7 | 1 | 0 | 1.611315 | 1.099641 | 0.508495 |
| 8 | 6 | 0 | 5.743413 | -0.615765 | -0.654735 |
| 9 | 1 | 0 | 1.865567 | -3.152770 | -0.309798 |
| 10 | 6 | 0 | 6.246758 | 0.737254 | -0.509549 |
| 11 | 6 | 0 | 5.427994 | 1.754102 | -0.146898 |
| 12 | 1 | 0 | 7.295627 | 0.930438 | -0.698920 |
| 13 | 6 | 0 | 5.826612 | 3.182891 | 0.029041 |
| 14 | 1 | 0 | 6.890035 | 3.313191 | -0.174275 |
| 15 | 1 | 0 | 5.252848 | 3.824311 | -0.648240 |
| 16 | 1 | 0 | 5.615322 | 3.514453 | 1.051147 |
| 17 | 8 | 0 | 6.489323 | -1.578249 | -0.996203 |
| 18 | 8 | 0 | 4.424694 | -3.130831 | -0.860104 |
| 19 | 8 | 0 | 0.277702 | -1.230172 | 0.384069 |
| 20 | 8 | 0 | 4.105385 | 1.569998 | 0.104003 |
| 21 | 6 | 0 | -0.636081 | -0.132211 | 1.900744 |
| 22 | 1 | 0 | 0.331157 | 0.179974 | 2.253824 |
| 23 | 1 | 0 | -1.003491 | -1.107893 | 2.166152 |
| 24 | 1 | 0 | 5.355365 | -2.786123 | -0.987864 |
| 25 | 6 | 0 | -1.276171 | 0.699919 | 0.831002 |
| 26 | 8 | 0 | -0.803666 | 1.777985 | 0.483659 |
| 27 | 7 | 0 | -2.422490 | 0.140910 | 0.315299 |
| 28 | 17 | 0 | -1.681258 | 0.817208 | 3.643359 |
| 29 | 1 | 0 | -2.662190 | -0.768627 | 0.681896 |
| 30 | 6 | 0 | -3.260013 | 0.639780 | -0.696797 |
| 31 | 6 | 0 | -4.515628 | 0.023577 | -0.926358 |
| 32 | 6 | 0 | -2.887371 | 1.722393 | -1.510306 |
| 33 | 6 | 0 | -5.349871 | 0.487405 | -1.945950 |
| 34 | 6 | 0 | -3.737494 | 2.172010 | -2.518505 |
| 35 | 1 | 0 | -1.934349 | 2.199347 | -1.337948 |
| 36 | 6 | 0 | -4.968607 | 1.559376 | -2.748472 |
| 37 | 1 | 0 | -6.305282 | 0.001578 | -2.103062 |
| 38 | 1 | 0 | -3.423291 | 3.009693 | -3.133739 |
| 39 | 1 | 0 | -5.625466 | 1.910032 | -3.537082 |
| 40 | 6 | 0 | -5.004391 | -1.092477 | -0.046484 |
| 41 | 9 | 0 | -6.153485 | -1.638735 | -0.483323 |
| 42 | 9 | 0 | -5.216125 | -0.681521 | 1.228607 |
| 43 | 9 | 0 | -4.096874 | -2.112668 | 0.041631 |

**11**

| Center Number | Atomic Number | Atomic Type | Coordinates (Angstroms) X | Y | Z |
|---|---|---|---|---|---|
| 1 | 6 | 0 | 0.812474 | 1.467690 | 0.423586 |
| 2 | 6 | 0 | 1.384131 | 1.037832 | -0.778949 |
| 3 | 6 | 0 | 2.633676 | 0.425691 | -0.711088 |
| 4 | 6 | 0 | 3.322416 | 0.227610 | 0.494102 |
| 5 | 6 | 0 | 2.706171 | 0.675543 | 1.698647 |
| 6 | 6 | 0 | 1.460693 | 1.289727 | 1.656381 |
| 7 | 1 | 0 | 0.901742 | 1.146541 | -1.740263 |
| 8 | 6 | 0 | 4.622669 | -0.420122 | 0.498714 |
| 9 | 1 | 0 | 0.988557 | 1.635476 | 2.568075 |
| 10 | 6 | 0 | 5.114290 | -0.823258 | -0.800200 |
| 11 | 6 | 0 | 4.391468 | -0.599889 | -1.925859 |
| 12 | 1 | 0 | 6.077972 | -1.312535 | -0.867798 |
| 13 | 6 | 0 | 4.789454 | -0.968426 | -3.315676 |
| 14 | 1 | 0 | 5.764908 | -1.455259 | -3.319173 |
| 15 | 1 | 0 | 4.049429 | -1.645572 | -3.754586 |
| 16 | 1 | 0 | 4.832788 | -0.074874 | -3.946951 |
| 17 | 8 | 0 | 5.264872 | -0.611634 | 1.563218 |
| 18 | 8 | 0 | 3.334799 | 0.503324 | 2.872909 |
| 19 | 8 | 0 | -0.406675 | 2.081315 | 0.506619 |
| 20 | 8 | 0 | 3.176180 | 0.010083 | -1.898948 |
| 21 | 6 | 0 | -1.122523 | 2.389263 | -0.693589 |
| 22 | 1 | 0 | -0.468981 | 2.810387 | -1.461421 |
| 23 | 1 | 0 | -1.859987 | 3.152940 | -0.406335 |
| 24 | 1 | 0 | 4.198417 | 0.048580 | 2.652637 |
| 25 | 6 | 0 | -1.868549 | 1.194775 | -1.291227 |
| 26 | 8 | 0 | -2.207263 | 1.203211 | -2.469869 |
| 27 | 7 | 0 | -2.137173 | 0.206794 | -0.382549 |
| 28 | 17 | 0 | -3.488078 | 4.987156 | 0.305374 |
| 29 | 1 | 0 | -1.789116 | 0.395341 | 0.549060 |
| 30 | 6 | 0 | -2.789033 | -1.023586 | -0.588014 |
| 31 | 6 | 0 | -2.663900 | -2.043692 | 0.385067 |
| 32 | 6 | 0 | -3.574178 | -1.266601 | -1.724458 |
| 33 | 6 | 0 | -3.322038 | -3.262605 | 0.210752 |
| 34 | 6 | 0 | -4.217664 | -2.492798 | -1.883175 |
| 35 | 1 | 0 | -3.665525 | -0.491536 | -2.471033 |
| 36 | 6 | 0 | -4.101755 | -3.494362 | -0.919961 |
| 37 | 1 | 0 | -3.214485 | -4.030886 | 0.966956 |
| 38 | 1 | 0 | -4.821597 | -2.658198 | -2.770073 |
| 39 | 1 | 0 | -4.608156 | -4.445249 | -1.045349 |
| 40 | 6 | 0 | -1.788268 | -1.848689 | 1.591102 |
| 41 | 9 | 0 | -1.828935 | -2.888273 | 2.442657 |
| 42 | 9 | 0 | -2.145603 | -0.739526 | 2.305199 |
| 43 | 9 | 0 | -0.487786 | -1.658798 | 1.250408 |

**TS17e**

| Center Number | Atomic Number | Atomic Type | Coordinates (Angstroms) X | Y | Z |
|---|---|---|---|---|---|
| 1 | 6 | 0 | -0.017654 | -1.408682 | -1.028839 |
| 2 | 6 | 0 | -0.693391 | -0.205312 | -1.461337 |
| 3 | 6 | 0 | -1.875411 | 0.167718 | -0.877778 |
| 4 | 6 | 0 | -2.553017 | -0.607443 | 0.100819 |
| 5 | 6 | 0 | -1.938137 | -1.854364 | 0.462400 |
| 6 | 6 | 0 | -0.753206 | -2.256612 | -0.101128 |
| 7 | 1 | 0 | -0.221538 | 0.422272 | -2.205461 |
| 8 | 6 | 0 | -3.795179 | -0.181912 | 0.652843 |
| 9 | 1 | 0 | -0.333049 | -3.217058 | 0.180696 |
| 10 | 6 | 0 | -4.296174 | 1.093114 | 0.155738 |
| 11 | 6 | 0 | -3.616249 | 1.794633 | -0.779365 |
| 12 | 1 | 0 | -5.234260 | 1.474678 | 0.541073 |
| 13 | 6 | 0 | -4.027447 | 3.103537 | -1.372191 |
| 14 | 1 | 0 | -4.974182 | 3.438579 | -0.946381 |
| 15 | 1 | 0 | -3.263833 | 3.866063 | -1.184150 |
| 16 | 1 | 0 | -4.138339 | 3.014462 | -2.458349 |
| 17 | 8 | 0 | -4.440484 | -0.858881 | 1.515976 |
| 18 | 8 | 0 | -2.579376 | -2.645929 | 1.360480 |
| 19 | 8 | 0 | 0.619998 | -2.078708 | -2.115348 |
| 20 | 8 | 0 | -2.434159 | 1.362900 | -1.300815 |
| 21 | 6 | 0 | 1.814776 | -2.738755 | -1.727896 |
| 22 | 1 | 0 | 2.481737 | -2.781967 | -2.594060 |
| 23 | 1 | 0 | 1.634089 | -3.768965 | -1.384893 |
| 24 | 1 | 0 | -3.410738 | -2.139230 | 1.602140 |
| 25 | 6 | 0 | 2.443257 | -1.937119 | -0.589171 |
| 26 | 8 | 0 | 3.552999 | -2.203699 | -0.107664 |
| 27 | 7 | 0 | 1.566333 | -0.984427 | -0.250212 |
| 28 | 6 | 0 | 1.688264 | -0.159960 | 0.874286 |
| 29 | 6 | 0 | 2.213295 | 1.149744 | 0.775252 |
| 30 | 6 | 0 | 1.230829 | -0.598731 | 2.127987 |
| 31 | 6 | 0 | 2.267412 | 1.975006 | 1.902112 |
| 32 | 6 | 0 | 1.281093 | 0.233639 | 3.245120 |
| 33 | 1 | 0 | 0.830638 | -1.602911 | 2.205633 |
| 34 | 6 | 0 | 1.798688 | 1.524639 | 3.135671 |
| 35 | 1 | 0 | 2.679868 | 2.972804 | 1.810796 |
| 36 | 1 | 0 | 0.919475 | -0.129824 | 4.202572 |
| 37 | 1 | 0 | 1.843878 | 2.176234 | 4.002284 |
| 38 | 6 | 0 | 2.741408 | 1.667129 | -0.536659 |
| 39 | 9 | 0 | 1.777537 | 1.790248 | -1.482717 |
| 40 | 9 | 0 | 3.300022 | 2.896671 | -0.406484 |
| 41 | 9 | 0 | 3.699239 | 0.868499 | -1.065091 |

**18e**

| Center Number | Atomic Number | Atomic Type | Coordinates (Angstroms) X | Y | Z |
|---|---|---|---|---|---|
| 1 | 6 | 0 | -0.012000 | -1.214370 | -0.834221 |
| 2 | 6 | 0 | -0.673874 | 0.063850 | -1.155478 |
| 3 | 6 | 0 | -1.928895 | 0.343231 | -0.702659 |
| 4 | 6 | 0 | -2.701054 | -0.528121 | 0.120658 |
| 5 | 6 | 0 | -2.075953 | -1.778324 | 0.470902 |

| | | | | | |
|---|---|---|---|---|---|
| 6 | 6 | 0 | -0.816988 | -2.097370 | 0.047057 |
| 7 | 1 | 0 | -0.144832 | 0.773493 | -1.776912 |
| 8 | 6 | 0 | -4.000475 | -0.177566 | 0.560266 |
| 9 | 1 | 0 | -0.391109 | -3.056022 | 0.331300 |
| 10 | 6 | 0 | -4.483841 | 1.126864 | 0.114124 |
| 11 | 6 | 0 | -3.721180 | 1.924555 | -0.665030 |
| 12 | 1 | 0 | -5.471948 | 1.452592 | 0.417600 |
| 13 | 6 | 0 | -4.099946 | 3.274451 | -1.184470 |
| 14 | 1 | 0 | -5.101840 | 3.548128 | -0.850410 |
| 15 | 1 | 0 | -3.390796 | 4.033663 | -0.836437 |
| 16 | 1 | 0 | -4.076284 | 3.285814 | -2.279756 |
| 17 | 8 | 0 | -4.722701 | -0.931995 | 1.296610 |
| 18 | 8 | 0 | -2.786340 | -2.640017 | 1.250087 |
| 19 | 8 | 0 | 0.352875 | -1.905877 | -2.101338 |
| 20 | 8 | 0 | -2.471598 | 1.567124 | -1.077941 |
| 21 | 6 | 0 | 1.566175 | -2.611475 | -1.937462 |
| 22 | 1 | 0 | 2.163054 | -2.553635 | -2.854743 |
| 23 | 1 | 0 | 1.419043 | -3.676138 | -1.695183 |
| 24 | 1 | 0 | -3.656687 | -2.168381 | 1.422555 |
| 25 | 6 | 0 | 2.295844 | -1.934060 | -0.786028 |
| 26 | 8 | 0 | 3.429955 | -2.194809 | -0.394057 |
| 27 | 7 | 0 | 1.426013 | -1.014225 | -0.288001 |
| 28 | 6 | 0 | 1.710510 | -0.225082 | 0.859324 |
| 29 | 6 | 0 | 2.458329 | 0.968247 | 0.762444 |
| 30 | 6 | 0 | 1.245304 | -0.630594 | 2.115223 |
| 31 | 6 | 0 | 2.720505 | 1.718248 | 1.913153 |
| 32 | 6 | 0 | 1.501413 | 0.131232 | 3.255208 |
| 33 | 1 | 0 | 0.673258 | -1.547699 | 2.181117 |
| 34 | 6 | 0 | 2.242619 | 1.307008 | 3.156422 |
| 35 | 1 | 0 | 3.299564 | 2.629596 | 1.830830 |
| 36 | 1 | 0 | 1.128627 | -0.202308 | 4.218736 |
| 37 | 1 | 0 | 2.452685 | 1.902408 | 4.038925 |
| 38 | 6 | 0 | 2.984011 | 1.474206 | -0.559399 |
| 39 | 9 | 0 | 1.993700 | 1.750390 | -1.443160 |
| 40 | 9 | 0 | 3.688064 | 2.622022 | -0.411331 |
| 41 | 9 | 0 | 3.810974 | 0.595708 | -1.169811 |

**TS18e**

| Center Number | Atomic Number | Atomic Type | Coordinates (Angstroms) X | Y | Z |
|---|---|---|---|---|---|
| 1 | 6 | 0 | 0.153047 | 0.839297 | -0.601438 |
| 2 | 6 | 0 | 0.748756 | -0.460276 | -0.498424 |
| 3 | 6 | 0 | 2.084421 | -0.567806 | -0.190506 |
| 4 | 6 | 0 | 2.905679 | 0.537937 | 0.138709 |
| 5 | 6 | 0 | 2.276737 | 1.824869 | 0.148618 |
| 6 | 6 | 0 | 0.940433 | 1.961426 | -0.153804 |
| 7 | 1 | 0 | 0.171606 | -1.341591 | -0.737626 |
| 8 | 6 | 0 | 4.283092 | 0.359543 | 0.484393 |
| 9 | 1 | 0 | 0.489091 | 2.948083 | -0.125941 |
| 10 | 6 | 0 | 4.760046 | -1.014063 | 0.454189 |
| 11 | 6 | 0 | 3.931686 | -2.035455 | 0.133619 |
| 12 | 1 | 0 | 5.795688 | -1.215947 | 0.699893 |
| 13 | 6 | 0 | 4.300707 | -3.482068 | 0.072591 |
| 14 | 1 | 0 | 5.351042 | -3.622279 | 0.330809 |
| 15 | 1 | 0 | 3.683027 | -4.063940 | 0.765042 |
| 16 | 1 | 0 | 4.125614 | -3.878539 | -0.933272 |
| 17 | 8 | 0 | 5.042967 | 1.324885 | 0.801524 |
| 18 | 8 | 0 | 3.018413 | 2.905868 | 0.492762 |
| 19 | 8 | 0 | -0.126175 | 1.145758 | -2.427999 |
| 20 | 8 | 0 | 2.622324 | -1.842036 | -0.181704 |
| 21 | 6 | 0 | -1.307242 | 1.868605 | -2.496449 |
| 22 | 1 | 0 | -1.977827 | 1.515343 | -3.298917 |
| 23 | 1 | 0 | -1.167703 | 2.954475 | -2.656394 |
| 24 | 1 | 0 | 3.933034 | 2.543330 | 0.681922 |
| 25 | 6 | 0 | -2.064887 | 1.685009 | -1.185181 |
| 26 | 8 | 0 | -3.181829 | 2.120407 | -0.931255 |
| 27 | 7 | 0 | -1.298986 | 0.914548 | -0.337510 |
| 28 | 6 | 0 | -1.796869 | 0.553293 | 0.954155 |
| 29 | 6 | 0 | -2.693638 | -0.524390 | 1.112996 |
| 30 | 6 | 0 | -1.404208 | 1.279053 | 2.082273 |
| 31 | 6 | 0 | -3.177910 | -0.840888 | 2.386453 |
| 32 | 6 | 0 | -1.883885 | 0.948935 | 3.349481 |
| 33 | 1 | 0 | -0.711815 | 2.102554 | 1.954089 |
| 34 | 6 | 0 | -2.777832 | -0.109050 | 3.502990 |
| 35 | 1 | 0 | -3.866493 | -1.668772 | 2.501091 |
| 36 | 1 | 0 | -1.564648 | 1.526393 | 4.211424 |
| 37 | 1 | 0 | -3.161913 | -0.368728 | 4.483907 |
| 38 | 6 | 0 | -3.112884 | -1.394786 | -0.046381 |
| 39 | 9 | 0 | -4.013898 | -2.330234 | 0.323434 |
| 40 | 9 | 0 | -3.666072 | -0.709888 | -1.070783 |
| 41 | 9 | 0 | -2.057242 | -2.078460 | -0.579653 |
| 42 | 8 | 0 | -1.021182 | -1.211989 | -3.457024 |
| 43 | 1 | 0 | -1.598487 | -1.491904 | -2.735866 |
| 44 | 1 | 0 | -0.600040 | -0.391334 | -3.094696 |

**19e**

| Center Number | Atomic Number | Atomic Type | Coordinates (Angstroms) X | Y | Z |
|---|---|---|---|---|---|
| 1 | 6 | 0 | 0.227941 | -0.692006 | -0.341903 |
| 2 | 6 | 0 | 0.782433 | 0.425108 | 0.294678 |
| 3 | 6 | 0 | 2.160851 | 0.586102 | 0.256139 |
| 4 | 6 | 0 | 3.012019 | -0.316713 | -0.403698 |
| 5 | 6 | 0 | 2.419992 | -1.433106 | -1.058712 |
| 6 | 6 | 0 | 1.039999 | -1.599572 | -1.041502 |
| 7 | 1 | 0 | 0.163712 | 1.135763 | 0.824791 |
| 8 | 6 | 0 | 4.447564 | -0.098298 | -0.414335 |
| 9 | 1 | 0 | 0.601497 | -2.443944 | -1.559996 |
| 10 | 6 | 0 | 4.896856 | 1.078515 | 0.296408 |
| 11 | 6 | 0 | 4.017133 | 1.906493 | 0.914194 |
| 12 | 1 | 0 | 5.956935 | 1.297205 | 0.331388 |
| 13 | 6 | 0 | 4.361873 | 3.144485 | 1.673074 |
| 14 | 1 | 0 | 5.439782 | 3.307956 | 1.671597 |
| 15 | 1 | 0 | 3.867326 | 4.013182 | 1.226136 |
| 16 | 1 | 0 | 4.013017 | 3.064132 | 2.707803 |
| 17 | 8 | 0 | 5.236260 | -0.883217 | -1.002623 |
| 18 | 8 | 0 | 3.198696 | -2.315727 | -1.713940 |
| 19 | 8 | 0 | -0.233668 | -2.656131 | 1.465600 |
| 20 | 8 | 0 | 2.677628 | 1.683417 | 0.902199 |
| 21 | 6 | 0 | -1.253492 | -3.065720 | 0.687074 |
| 22 | 1 | 0 | -2.161508 | -3.432975 | 1.228567 |
| 23 | 1 | 0 | -1.009688 | -3.915908 | -0.002841 |
| 24 | 1 | 0 | 4.134365 | -1.984222 | -1.599846 |
| 25 | 6 | 0 | -1.888866 | -2.015682 | -0.259053 |
| 26 | 8 | 0 | -2.980066 | -2.181873 | -0.795182 |
| 27 | 7 | 0 | -1.198094 | -0.801248 | -0.417620 |
| 28 | 6 | 0 | -1.874956 | 0.258998 | -1.116347 |
| 29 | 6 | 0 | -2.825131 | 1.077466 | -0.474587 |
| 30 | 6 | 0 | -1.582373 | 0.485526 | -2.463521 |
| 31 | 6 | 0 | -3.470811 | 2.086006 | -1.197080 |
| 32 | 6 | 0 | -2.223945 | 1.498540 | -3.174938 |
| 33 | 1 | 0 | -0.844451 | -0.144814 | -2.948431 |
| 34 | 6 | 0 | -3.176142 | 2.297111 | -2.542894 |
| 35 | 1 | 0 | -4.199729 | 2.712329 | -0.697771 |
| 36 | 1 | 0 | -1.984100 | 1.655791 | -4.221817 |
| 37 | 1 | 0 | -3.684426 | 3.084876 | -3.088900 |
| 38 | 6 | 0 | -3.138605 | 0.926183 | 0.992248 |
| 39 | 9 | 0 | -2.027555 | 1.088604 | 1.769444 |
| 40 | 9 | 0 | -4.031754 | 1.845788 | 1.416985 |
| 41 | 9 | 0 | -3.646039 | -0.283699 | 1.313440 |
| 42 | 8 | 0 | -1.348043 | -1.544990 | 3.513220 |
| 43 | 1 | 0 | -1.684656 | -0.725418 | 3.131655 |
| 44 | 1 | 0 | -0.873777 | -1.979903 | 2.716496 |

**TS19e**

| Center Number | Atomic Number | Atomic Type | Coordinates (Angstroms) X | Y | Z |
|---|---|---|---|---|---|
| 1 | 6 | 0 | 0.820869 | -0.092050 | 0.136436 |
| 2 | 6 | 0 | 2.052369 | 0.519331 | 0.462238 |
| 3 | 6 | 0 | 3.234948 | -0.078223 | 0.048880 |
| 4 | 6 | 0 | 3.273927 | -1.279930 | -0.676357 |
| 5 | 6 | 0 | 2.025899 | -1.883703 | -1.001182 |
| 6 | 6 | 0 | 0.831790 | -1.297330 | -0.604072 |
| 7 | 1 | 0 | 2.102366 | 1.444399 | 1.016384 |
| 8 | 6 | 0 | 4.542199 | -1.862620 | -1.072798 |
| 9 | 1 | 0 | -0.097021 | -1.783822 | -0.870529 |
| 10 | 6 | 0 | 5.714737 | -1.119464 | -0.663685 |
| 11 | 6 | 0 | 5.604738 | 0.038831 | 0.033329 |
| 12 | 1 | 0 | 6.695091 | -1.501584 | -0.920257 |
| 13 | 6 | 0 | 6.736087 | 0.889722 | 0.506195 |
| 14 | 1 | 0 | 7.692359 | 0.449986 | 0.221870 |
| 15 | 1 | 0 | 6.661694 | 1.893700 | 0.075466 |
| 16 | 1 | 0 | 6.700783 | 0.996150 | 1.595332 |
| 17 | 8 | 0 | 4.612598 | -2.950875 | -1.703049 |
| 18 | 8 | 0 | 2.002946 | -3.027640 | -1.711503 |
| 19 | 8 | 0 | -1.620944 | -2.470008 | 3.154668 |
| 20 | 8 | 0 | 4.402406 | 0.562244 | 0.389682 |
| 21 | 6 | 0 | -0.763384 | -1.620551 | 2.388972 |
| 22 | 1 | 0 | -0.146993 | -2.262164 | 1.763956 |
| 23 | 1 | 0 | -0.117665 | -1.016935 | 3.036247 |
| 24 | 1 | 0 | 2.960871 | -3.235788 | -1.907828 |
| 25 | 6 | 0 | -1.644932 | -0.706392 | 1.548003 |
| 26 | 8 | 0 | -2.511743 | 0.004720 | 2.020732 |
| 27 | 7 | 0 | -0.437314 | 0.444086 | 0.496440 |
| 28 | 6 | 0 | -0.441852 | 1.763840 | 1.065904 |
| 29 | 6 | 0 | -0.611977 | 2.942236 | 0.295573 |
| 30 | 6 | 0 | -0.257177 | 1.917907 | 2.447933 |
| 31 | 6 | 0 | -0.579941 | 4.199182 | 0.914357 |
| 32 | 6 | 0 | -0.238714 | 3.170536 | 3.056573 |
| 33 | 1 | 0 | -0.111743 | 1.034392 | 3.055831 |
| 34 | 6 | 0 | -0.396582 | 4.321175 | 2.288014 |
| 35 | 1 | 0 | -0.379008 | 5.303045 | 2.749008 |
| 36 | 1 | 0 | -2.287779 | -1.892257 | 3.559610 |
| 37 | 6 | 0 | -2.780958 | -1.168979 | -0.616262 |
| 38 | 8 | 0 | -3.814686 | -1.708305 | -1.017609 |
| 39 | 8 | 0 | -2.215245 | -0.133257 | -1.194500 |
| 40 | 8 | 0 | -2.104432 | -1.661056 | 0.440517 |
| 41 | 1 | 0 | -1.392385 | 0.207067 | -0.561997 |
| 42 | 19 | 0 | -4.164950 | 0.271295 | -3.079520 |
| 43 | 19 | 0 | -3.426479 | -3.902349 | 1.267663 |
| 44 | 1 | 0 | -0.089275 | 3.242777 | 4.129393 |
| 45 | 1 | 0 | -0.705333 | 5.086965 | 0.306957 |
| 46 | 6 | 0 | -0.904878 | 2.934327 | -1.184804 |

| | | | | | |
|---|---|---|---|---|---|
| 47 | 9 | 0 | -0.184281 | 2.023379 | -1.877340 |
| 48 | 9 | 0 | -2.218628 | 2.670465 | -1.432108 |
| 49 | 9 | 0 | -0.652405 | 4.135247 | -1.753913 |

**16**

| Center | Atomic | Atomic | Coordinates (Angstroms) | | |
|---|---|---|---|---|---|
| Number | Number | Type | X | Y | Z |
| 1 | 6 | 0 | 1.184370 | 0.261432 | -0.266982 |
| 2 | 6 | 0 | 2.437694 | 0.827406 | 0.044525 |
| 3 | 6 | 0 | 3.578051 | 0.073741 | -0.187757 |
| 4 | 6 | 0 | 3.546353 | -1.224060 | -0.727683 |
| 5 | 6 | 0 | 2.270115 | -1.770371 | -1.049490 |
| 6 | 6 | 0 | 1.113902 | -1.039662 | -0.820814 |
| 7 | 1 | 0 | 2.532735 | 1.828302 | 0.442456 |
| 8 | 6 | 0 | 4.770729 | -1.962286 | -0.947866 |
| 9 | 1 | 0 | 0.149927 | -1.468271 | -1.067601 |
| 10 | 6 | 0 | 5.985511 | -1.265747 | -0.579007 |
| 11 | 6 | 0 | 5.946892 | -0.010524 | -0.068055 |
| 12 | 1 | 0 | 6.938345 | -1.761113 | -0.718937 |
| 13 | 6 | 0 | 7.125063 | 0.808225 | 0.343325 |
| 14 | 1 | 0 | 8.051594 | 0.257794 | 0.178082 |
| 15 | 1 | 0 | 7.156731 | 1.741585 | -0.228353 |
| 16 | 1 | 0 | 7.051156 | 1.074407 | 1.402898 |
| 17 | 8 | 0 | 4.772140 | -3.127268 | -1.429518 |
| 18 | 8 | 0 | 2.193283 | -3.009095 | -1.571884 |
| 19 | 8 | 0 | -0.477422 | -0.916518 | 3.743553 |
| 20 | 8 | 0 | 4.779655 | 0.659215 | 0.130438 |
| 21 | 6 | 0 | -0.761697 | -1.868814 | 2.743604 |
| 22 | 1 | 0 | -1.137977 | -2.807450 | 3.176062 |
| 23 | 1 | 0 | 0.141066 | -2.116149 | 2.169627 |
| 24 | 1 | 0 | 3.139106 | -3.331291 | -1.635418 |
| 25 | 6 | 0 | -1.802340 | -1.332563 | 1.776524 |
| 26 | 8 | 0 | -2.335013 | -0.249249 | 1.938741 |
| 27 | 7 | 0 | -0.001707 | 0.948695 | -0.093106 |
| 28 | 6 | 0 | -0.184917 | 2.125526 | 0.658469 |
| 29 | 6 | 0 | -0.975252 | 3.186254 | 0.160857 |
| 30 | 6 | 0 | 0.372075 | 2.253421 | 1.940626 |
| 31 | 6 | 0 | -1.187821 | 4.329474 | 0.938421 |
| 32 | 6 | 0 | 0.171360 | 3.405141 | 2.696767 |
| 33 | 1 | 0 | 0.932078 | 1.421578 | 2.352540 |
| 34 | 6 | 0 | -0.609962 | 4.449667 | 2.199342 |
| 35 | 1 | 0 | -0.774707 | 5.345355 | 2.788497 |
| 36 | 1 | 0 | -1.089665 | -0.178185 | 3.579729 |
| 37 | 6 | 0 | -2.833688 | -1.616778 | -0.393372 |
| 38 | 8 | 0 | -3.919907 | -2.177481 | -0.553798 |
| 39 | 8 | 0 | -2.236380 | -0.742619 | -1.032568 |
| 40 | 8 | 0 | -2.055722 | -2.168559 | 0.763081 |
| 41 | 1 | 0 | -0.837031 | 0.492797 | -0.462391 |
| 42 | 19 | 0 | -4.283884 | 0.170167 | -2.535499 |
| 43 | 19 | 0 | -4.041210 | -4.429856 | 0.941696 |
| 44 | 1 | 0 | 0.610155 | 3.475400 | 3.687251 |
| 45 | 1 | 0 | -1.799546 | 5.131125 | 0.542416 |
| 46 | 6 | 0 | -1.612512 | 3.103313 | -1.198219 |
| 47 | 9 | 0 | -0.737841 | 2.793671 | -2.178833 |
| 48 | 9 | 0 | -2.583246 | 2.135016 | -1.237088 |
| 49 | 9 | 0 | -2.217118 | 4.253888 | -1.557022 |

**23-24 TS**

| Center | Atomic | Atomic | Coordinates (Angstroms) | | |
|---|---|---|---|---|---|
| Number | Number | Type | X | Y | Z |
| 1 | 6 | 0 | -0.135438 | -0.940014 | 0.171385 |
| 2 | 6 | 0 | -0.698608 | 0.340899 | 0.039494 |
| 3 | 6 | 0 | -2.079591 | 0.458944 | -0.017581 |
| 4 | 6 | 0 | -2.944442 | -0.644148 | 0.053189 |
| 5 | 6 | 0 | -2.355240 | -1.929062 | 0.193780 |
| 6 | 6 | 0 | -0.973490 | -2.070059 | 0.259117 |
| 7 | 1 | 0 | -0.085792 | 1.226379 | -0.033012 |
| 8 | 6 | 0 | -4.383159 | -0.459602 | -0.013740 |
| 9 | 1 | 0 | -0.557100 | -3.060156 | 0.364577 |
| 10 | 6 | 0 | -4.819550 | 0.911383 | -0.162169 |
| 11 | 6 | 0 | -3.926234 | 1.931091 | -0.227435 |
| 12 | 1 | 0 | -5.880246 | 1.121445 | -0.222013 |
| 13 | 6 | 0 | -4.258015 | 3.378157 | -0.381540 |
| 14 | 1 | 0 | -5.337493 | 3.521042 | -0.435718 |
| 15 | 1 | 0 | -3.862766 | 3.949844 | 0.464518 |
| 16 | 1 | 0 | -3.797886 | 3.777463 | -1.291355 |
| 17 | 8 | 0 | -5.182173 | -1.429354 | 0.052539 |
| 18 | 8 | 0 | -3.142018 | -3.020312 | 0.278309 |
| 19 | 8 | 0 | 3.662425 | -1.164281 | 1.053174 |
| 20 | 8 | 0 | -2.585500 | 1.730229 | -0.155918 |
| 21 | 6 | 0 | 3.374491 | -2.271707 | 0.324763 |
| 22 | 1 | 0 | 3.446695 | -3.239125 | 0.879244 |
| 23 | 1 | 0 | 3.991984 | -2.431433 | -0.589233 |
| 24 | 1 | 0 | -4.078821 | -2.681150 | 0.215371 |
| 25 | 6 | 0 | 1.928032 | -2.239021 | -0.164899 |
| 26 | 8 | 0 | 1.400814 | -3.127112 | -0.826739 |
| 27 | 7 | 0 | 1.272161 | -1.088925 | 0.270863 |
| 28 | 6 | 0 | 2.050554 | 0.018448 | 0.845756 |
| 29 | 6 | 0 | 2.497982 | 1.083655 | -0.001935 |

| | | | | | |
|---|---|---|---|---|---|
| 30 | 6 | 0 | 1.749338 | 0.366558 | 2.194188 |
| 31 | 6 | 0 | 2.843301 | 2.324725 | 0.550017 |
| 32 | 6 | 0 | 2.087778 | 1.601853 | 2.713850 |
| 33 | 1 | 0 | 1.290802 | -0.391770 | 2.821134 |
| 34 | 6 | 0 | 2.661396 | 2.596356 | 1.902216 |
| 35 | 1 | 0 | 3.239773 | 3.092323 | -0.105739 |
| 36 | 1 | 0 | 1.887582 | 1.807982 | 3.762751 |
| 37 | 1 | 0 | 2.922068 | 3.567625 | 2.309380 |
| 38 | 6 | 0 | 2.655017 | 0.875274 | -1.471085 |
| 39 | 9 | 0 | 1.471869 | 0.622206 | -2.109527 |
| 40 | 9 | 0 | 3.185071 | 1.956671 | -2.097319 |
| 41 | 9 | 0 | 3.458282 | -0.176767 | -1.797276 |

**24**

| Center | Atomic | Atomic | Coordinates (Angstroms) | | |
|---|---|---|---|---|---|
| Number | Number | Type | X | Y | Z |
| 1 | 6 | 0 | -0.102130 | -1.018119 | 0.278964 |
| 2 | 6 | 0 | -0.640707 | 0.284853 | 0.259887 |
| 3 | 6 | 0 | -2.012629 | 0.431415 | 0.121821 |
| 4 | 6 | 0 | -2.898193 | -0.651755 | 0.001115 |
| 5 | 6 | 0 | -2.335731 | -1.955186 | 0.026659 |
| 6 | 6 | 0 | -0.964360 | -2.132743 | 0.166757 |
| 7 | 1 | 0 | -0.028649 | 1.169945 | 0.341815 |
| 8 | 6 | 0 | -4.324551 | -0.429774 | -0.143637 |
| 9 | 1 | 0 | -0.561970 | -3.133660 | 0.172384 |
| 10 | 6 | 0 | -4.731727 | 0.958382 | -0.154861 |
| 11 | 6 | 0 | -3.822002 | 1.958453 | -0.034505 |
| 12 | 1 | 0 | -5.782676 | 1.197078 | -0.261553 |
| 13 | 6 | 0 | -4.123402 | 3.420651 | -0.033523 |
| 14 | 1 | 0 | -5.195028 | 3.592535 | -0.137590 |
| 15 | 1 | 0 | -3.775856 | 3.878599 | 0.898327 |
| 16 | 1 | 0 | -3.599762 | 3.916154 | -0.857703 |
| 17 | 8 | 0 | -5.140122 | -1.382824 | -0.251635 |
| 18 | 8 | 0 | -3.140346 | -3.032822 | -0.080873 |
| 19 | 8 | 0 | 3.479907 | -0.853226 | 1.100733 |
| 20 | 8 | 0 | -2.493016 | 1.721993 | 0.102893 |
| 21 | 6 | 0 | 3.417244 | -2.103288 | 0.456945 |
| 22 | 1 | 0 | 3.796220 | -2.896516 | 1.113301 |
| 23 | 1 | 0 | 3.995043 | -2.127640 | -0.478925 |
| 24 | 1 | 0 | -4.065250 | -2.667863 | -0.168271 |
| 25 | 6 | 0 | 1.957722 | -2.355730 | 0.142087 |
| 26 | 8 | 0 | 1.527674 | -3.415807 | -0.310014 |
| 27 | 7 | 0 | 1.279714 | -1.207072 | 0.439216 |
| 28 | 6 | 0 | 2.248485 | -0.037385 | 0.887954 |
| 29 | 6 | 0 | 2.444196 | 1.018235 | -0.137281 |
| 30 | 6 | 0 | 1.837270 | 0.517616 | 2.189705 |
| 31 | 6 | 0 | 2.213439 | 2.370193 | 0.144961 |
| 32 | 6 | 0 | 1.623938 | 1.854552 | 2.408463 |
| 33 | 1 | 0 | 1.719018 | -0.207375 | 2.990593 |
| 34 | 6 | 0 | 1.777764 | 2.820768 | 1.385740 |
| 35 | 1 | 0 | 2.384432 | 3.086607 | -0.653667 |
| 36 | 1 | 0 | 1.324938 | 2.173264 | 3.406332 |
| 37 | 1 | 0 | 1.600307 | 3.874466 | 1.571264 |
| 38 | 6 | 0 | 2.990063 | 0.648982 | -1.458025 |
| 39 | 9 | 0 | 2.354902 | -0.418838 | -2.048739 |
| 40 | 9 | 0 | 2.929164 | 1.660102 | -2.367215 |
| 41 | 9 | 0 | 4.315430 | 0.265395 | -1.444860 |

**24-20-H$^+$ TS**

| Center | Atomic | Atomic | Coordinates (Angstroms) | | |
|---|---|---|---|---|---|
| Number | Number | Type | X | Y | Z |
| 1 | 6 | 0 | -0.168245 | -1.022675 | 0.295169 |
| 2 | 6 | 0 | -0.736657 | 0.267984 | 0.214909 |
| 3 | 6 | 0 | -2.105205 | 0.382176 | 0.033372 |
| 4 | 6 | 0 | -2.966253 | -0.723967 | -0.074842 |
| 5 | 6 | 0 | -2.377673 | -2.014867 | 0.009476 |
| 6 | 6 | 0 | -1.008495 | -2.159038 | 0.193006 |
| 7 | 1 | 0 | -0.141643 | 1.166569 | 0.282332 |
| 8 | 6 | 0 | -4.390130 | -0.537418 | -0.267609 |
| 9 | 1 | 0 | -0.580263 | -3.148939 | 0.238927 |
| 10 | 6 | 0 | -4.826309 | 0.840989 | -0.337015 |
| 11 | 6 | 0 | -3.941778 | 1.863980 | -0.225738 |
| 12 | 1 | 0 | -5.878675 | 1.052495 | -0.481025 |
| 13 | 6 | 0 | -4.273770 | 3.318671 | -0.282601 |
| 14 | 1 | 0 | -5.345043 | 3.463461 | -0.424921 |
| 15 | 1 | 0 | -3.965385 | 3.814575 | 0.643645 |
| 16 | 1 | 0 | -3.735373 | 3.798791 | -1.106399 |
| 17 | 8 | 0 | -5.185080 | -1.509864 | -0.367586 |
| 18 | 8 | 0 | -3.159749 | -3.111253 | -0.087771 |
| 19 | 8 | 0 | 3.407642 | -0.686593 | 1.245732 |
| 20 | 8 | 0 | -2.612511 | 1.661430 | -0.043983 |
| 21 | 6 | 0 | 3.368451 | -1.963522 | 0.642126 |
| 22 | 1 | 0 | 3.760821 | -2.707995 | 1.343867 |
| 23 | 1 | 0 | 3.970334 | -2.006498 | -0.276378 |
| 24 | 1 | 0 | -4.088010 | -2.766047 | -0.213350 |
| 25 | 6 | 0 | 1.923315 | -2.281742 | 0.305085 |
| 26 | 8 | 0 | 1.571479 | -3.397274 | -0.097524 |
| 27 | 7 | 0 | 1.201153 | -1.156987 | 0.501011 |
| 28 | 6 | 0 | 2.268940 | 0.166317 | 0.969021 |
| 29 | 6 | 0 | 2.459131 | 1.132110 | -0.115752 |

| | | | | | |
|---|---|---|---|---|---|
| 30 | 6 | 0 | 1.783389 | 0.769398 | 2.200404 |
| 31 | 6 | 0 | 2.041821 | 2.462611 | 0.011554 |
| 32 | 6 | 0 | 1.396612 | 2.087070 | 2.287247 |
| 33 | 1 | 0 | 1.732306 | 0.113746 | 3.064923 |
| 34 | 6 | 0 | 1.483953 | 2.962324 | 1.183762 |
| 35 | 1 | 0 | 2.181313 | 3.121880 | -0.839717 |
| 36 | 1 | 0 | 1.027478 | 2.458990 | 3.241553 |
| 37 | 1 | 0 | 1.176416 | 3.999587 | 1.259498 |
| 38 | 6 | 0 | 3.126479 | 0.704222 | -1.367637 |
| 39 | 9 | 0 | 2.563213 | -0.406764 | -1.945130 |
| 40 | 9 | 0 | 3.117332 | 1.662361 | -2.331840 |
| 41 | 9 | 0 | 4.447097 | 0.362292 | -1.209297 |

---------------------------------------------------------------------

**20-H$^+$**

| Center Number | Atomic Number | Atomic Type | Coordinates (Angstroms) X | Y | Z |
|---|---|---|---|---|---|
| 1 | 6 | 0 | -0.722419 | -0.989176 | 0.366409 |
| 2 | 6 | 0 | -1.479563 | 0.186477 | 0.637119 |
| 3 | 6 | 0 | -2.798750 | 0.269827 | 0.244774 |
| 4 | 6 | 0 | -3.463350 | -0.778484 | -0.428645 |
| 5 | 6 | 0 | -2.706356 | -1.955467 | -0.699127 |
| 6 | 6 | 0 | -1.377262 | -2.058402 | -0.315074 |
| 7 | 1 | 0 | -1.007028 | 1.014880 | 1.151391 |
| 8 | 6 | 0 | -4.845170 | -0.644641 | -0.818902 |
| 9 | 1 | 0 | -0.822476 | -2.959370 | -0.531375 |
| 10 | 6 | 0 | -5.467414 | 0.618850 | -0.471201 |
| 11 | 6 | 0 | -4.775166 | 1.588308 | 0.176088 |
| 12 | 1 | 0 | -6.504533 | 0.783635 | -0.737046 |
| 13 | 6 | 0 | -5.308224 | 2.922988 | 0.583566 |
| 14 | 1 | 0 | -6.353376 | 3.026594 | 0.290066 |
| 15 | 1 | 0 | -5.228119 | 3.049588 | 1.668369 |
| 16 | 1 | 0 | -4.725474 | 3.724112 | 0.116736 |
| 17 | 8 | 0 | -5.471731 | -1.561354 | -1.421892 |
| 18 | 8 | 0 | -3.304035 | -2.985941 | -1.343109 |
| 19 | 8 | 0 | 3.180097 | -0.537411 | 1.786848 |
| 20 | 8 | 0 | -3.474338 | 1.439951 | 0.535125 |
| 21 | 6 | 0 | 2.843072 | -1.791942 | 1.165489 |
| 22 | 1 | 0 | 2.968675 | -2.515187 | 1.977087 |
| 23 | 1 | 0 | 3.561461 | -2.057164 | 0.390064 |
| 24 | 1 | 0 | -4.240603 | -2.684063 | -1.513920 |
| 25 | 6 | 0 | 1.423987 | -1.978897 | 0.595528 |
| 26 | 8 | 0 | 1.269535 | -3.091065 | 0.035131 |
| 27 | 7 | 0 | 0.580704 | -0.964438 | 0.804112 |
| 28 | 6 | 0 | 3.067042 | 0.680135 | 1.186591 |
| 29 | 6 | 0 | 3.520647 | 1.047174 | -0.102335 |
| 30 | 6 | 0 | 2.548181 | 1.685203 | 2.020207 |
| 31 | 6 | 0 | 3.389304 | 2.381739 | -0.521461 |
| 32 | 6 | 0 | 2.441650 | 3.002493 | 1.595631 |
| 33 | 1 | 0 | 2.221401 | 1.387394 | 3.010520 |
| 34 | 6 | 0 | 2.852019 | 3.358198 | 0.307541 |
| 35 | 1 | 0 | 3.736016 | 2.649236 | -1.512388 |
| 36 | 1 | 0 | 2.029304 | 3.750208 | 2.266675 |
| 37 | 1 | 0 | 2.766251 | 4.381776 | -0.041214 |
| 38 | 6 | 0 | 4.176016 | 0.085649 | -1.051945 |
| 39 | 9 | 0 | 3.301685 | -0.792061 | -1.613633 |
| 40 | 9 | 0 | 4.781860 | 0.717707 | -2.083382 |
| 41 | 9 | 0 | 5.136414 | -0.661301 | -0.447282 |

---------------------------------------------------------------------
